# Supplementary material for: How mono- and diphosphine ligands alter regioselectivity of the Rh-catalyzed annulative cleavage of bicyclo[1.1.0]butanes
Source: Nat Commun. 2022 Nov 26;13:7292. doi: 10.1038/s41467-022-34837-x (PMC9701199; doi:10.1038/s41467-022-34837-x)
Supplement: Supplementary file 1 — Supplementary Information [file 41467_2022_34837_MOESM1_ESM.pdf]

Supplementary Information

How Mono- and Diphosphine Ligands Alter Regioselectivity of the Rh-Catalyzed Annulative Cleavage of Bicyclo[1.1.0]butanes

Pan-Pan Chen[1], Peter Wipf[2*], and K. N. Houk[1*]

[1]Department of Chemistry and Biochemistry, University of California, Los Angeles, California 90095, United States
[2]Department of Chemistry, University of Pittsburgh, 219 Parkman Avenue, Pittsburgh, Pennsylvania 15260, United States

*Corresponding authors: houk@chem.ucla.edu; pwipf@pitt.edu

Table of Contents

I. **Supplementary Methods** **S-2**
a. Computational methods **S-2**
b. Elucidation of the choice of computational methods **S-2**
II. **Supplementary Discussions** **S-3**
a. DFT-optimized structures of selected intermediates and transition states involved in Rh(I)/PPh_3 catalysis **S-3**
b. Exploration of the reference point of catalyst **S-4**
c. Alternative transition states of rhodium carbenoid formation involved in Rh/PPh_3 case **S-5**
d. Alternative transition states of alkene carbometalation involved in Rh/PPh_3 case **S-6**
e. Alternative transition states of reductive elimination involved in Rh/PPh_3 case **S-6**
f. IRC analyses of transition states involved in Rh/PPh_3 case **S-7**
g. Alternative transition states of *endo* oxidative addition involved in Rh/PPh_3 case **S-8**
h. Alternative transition states of ionic species generation **S-8**
i. Origins of regioselectivity of Rh(I)/PPh_3-catalyzed cycloisomerizations **S-9**
j. Origins of diastereoselectivity of Rh(I)/PPh_3-catalyzed cycloisomerizations **S-10**
k. DFT-optimized structures of selected intermediates and transition states involved in Rh(I)/dppe catalysis **S-11**
l. Alternative transition states of central C–C bond cleavage involved in Rh/dppe case **S-12**
m. IRC analyses of transition states involved in Rh/dppe case **S-13**
n. Origins of regioselectivity for Rh(I)/dppe-catalyzed cycloisomerizations **S-14**
o. Origins of diastereoselectivity for Rh(I)/dppe-catalyzed cycloisomerizations **S-14**
III. **Supplementary Tables** **S-16**
IV. **Supplementary References** **S-18**

I. Supplementary Methods

a. Computational methods

All density functional theory (DFT) calculations were conducted with Gaussian 16[1]. Geometry optimizations of all intermediates and transition states were performed at the B3LYP[2] level of theory with a def2-SVP[3] basis set including Grimme's D3 dispersion corrections[4]. Based on the optimized structures, vibrational frequencies were calculated at the same level of theory to evaluate zero-point vibrational energy (ZPVE) and thermal corrections at 298 K. The single-point energies were computed with a PBE0[5] functional and def2-TZVPP[3,6] basis set, including solvation energy corrections and Grimme's D3 dispersion corrections. The solvation energies were evaluated by a self-consistent reaction field (SCRF) using a CPCM model[7]. Extensive conformational searches for intermediates and transition states were conducted to ensure that the lowest energy conformers were located. Intrinsic reaction coordinate (IRC) calculations of the transitions states were performed to verify their locations in the free energy surface. The 3D diagrams of molecules were generated using CYLview[8], all hydrogens are hidden for simplicity, except at the stereogenic carbons. Certain fragments of the *p*-tolylsulfonyl group are simplified for clarity.

As it is known that in solution energies for association/dissociation processes are overestimated/underestimated, in order to adjust the Gibbs free energies from 1 atm to 1 mol L^{-1}, a correction of $RT\ln(c_s/c_g)$ is added to the energies of all species[9]. c_s is the standard molar concentration in solution (1 mol L^{-1}), c_g is the standard molar concentration in the gas phase (0.0446 mol L^{-1}), and R is the gas constant.

b. Elucidation of the choice of computational methods

In organometallic reaction systems, PBE0 is one of the reliable density functionals for the energy calculations and shows a good performance with the addition of Grimme's D3 dispersion corrections[10–12]. In our study, we performed calculations regarding regioselectivity with different single-point methods. As shown in the Supplementary Table 1, all the tested methods showed consistent selectivity and were consistent with the experimental observations[13]. Therefore, based on our calculations and literature reports, we selected PBE0-D3 as the single-point calculation method.

Supplementary Table 1. Methods benchmark for energies. Geometries of the structures are optimized with B3LYP-D3 functional. TS, transition state. Energies are in kcal mol^{-1}.

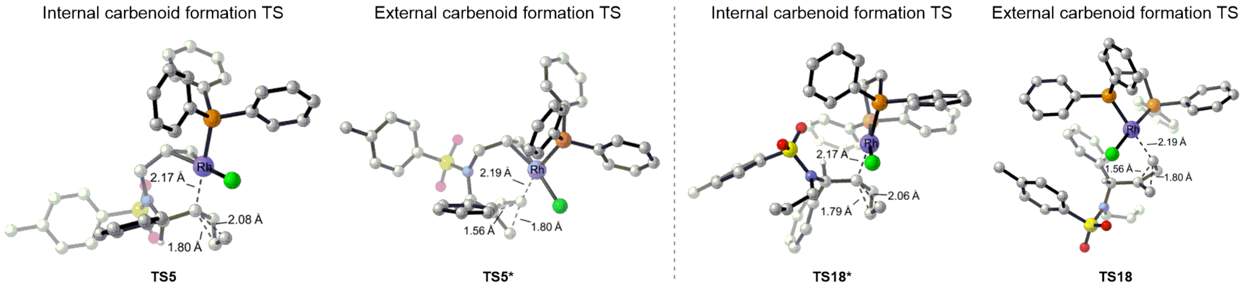

	M06[14]	M06-2X[14a]	ωB97X-D[15]	B3LYP-D3	PBE0-D3
ΔΔ*G*(**TS5*–TS5**)	0.7	2.3	3.7	5.8	2.9
ΔΔ*G*(**TS18–TS18***)	–7.2	–3.7	–4.5	–0.8	–4.0

II. Supplementary Discussions

a. DFT-optimized structures of selected intermediates and transition states involved in Rh(I)/PPh_3 catalysis

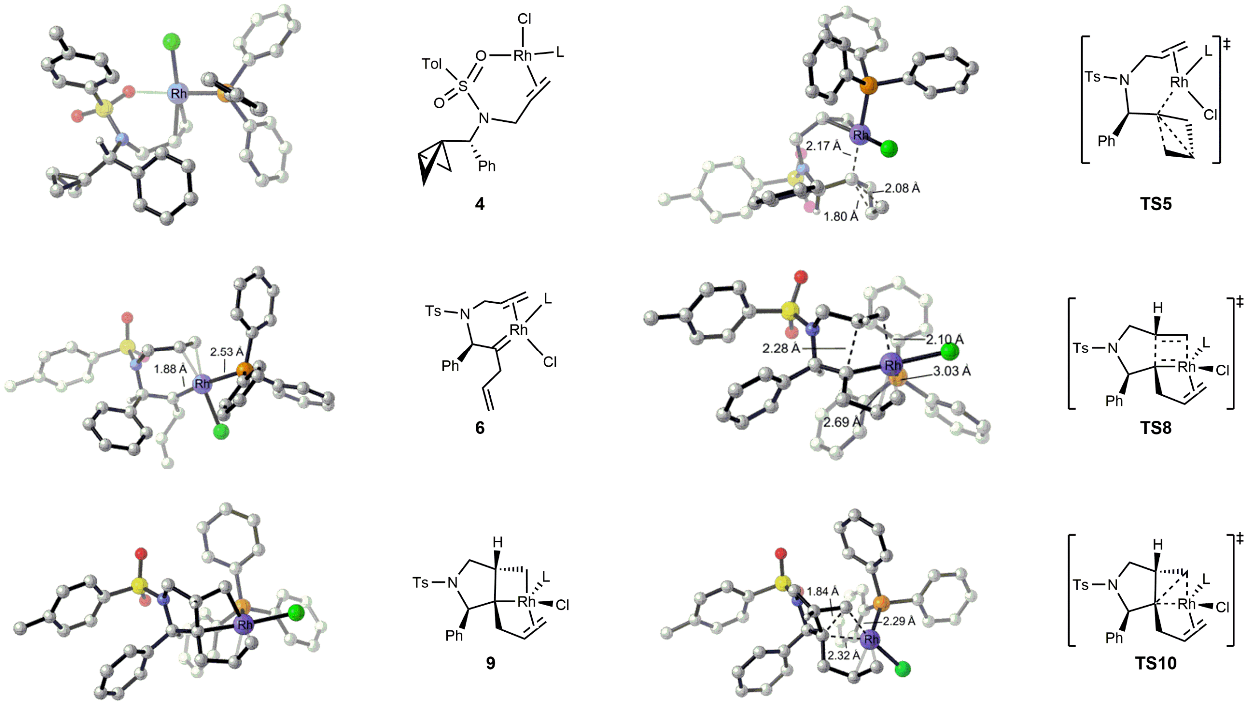

Supplementary Fig. 1. DFT-optimized structures of selected intermediates and transition states involved in Rh(I)/PPh_3-catalyzed cycloisomerizations. L = PPh_3.

b. Exploration of the reference point of catalyst

For both the Rh/PPh_3 and Rh/dppe cases, starting from the catalyst precursor (**Rh-I** or **Rh-II**), the competitive complexation of the substrate (**1**) and ligand (PPh_3 or dppe) leads to a stable catalyst-substrate complex (**4** or **16**). Therefore, we chose **4** and **16** as the zero level of catalyst in the relative energies calculations for Rh/PPh_3 and Rh/dppe cases, respectively.

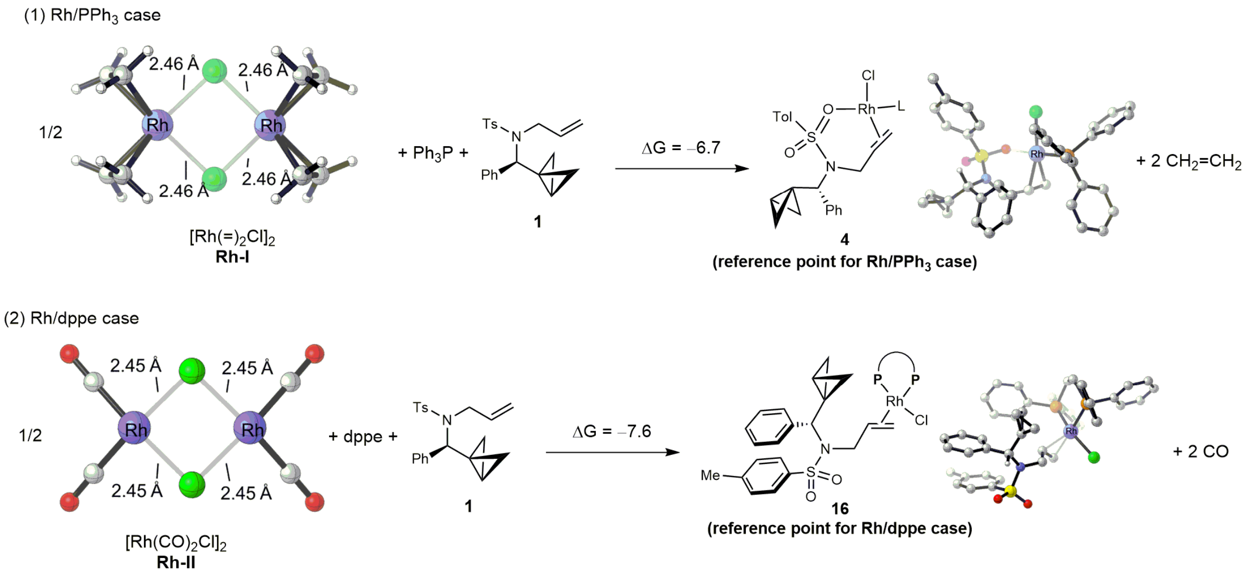

Supplementary Fig. 2. Free energy changes of catalyst-substrate complex formation starting from catalyst precursor. Free energies are in kcal mol^{-1}. L = PPh_3.

c. Alternative transition states of rhodium carbenoid formation involved in Rh/PPh_3 case

We explored different ways of central C–C bond activation. For C–C bond activation with internal carbenoid formation, alkene coordination effectively facilitates C–C bond cleavage (via **TS5**). While without the alkene assistance, the corresponding transition state **TS-S1** is less favorable by 24.1 kcal mol^{-1} compared to **TS5**. For C–C bond activation with external carbenoid formation, the alkene coordination is also indispensable. Although **TS-S2** has an extra coordination of oxygen, it is 20.3 kcal mol^{-1} less favorable than **TS5***. Therefore, the competition between **TS5** and **TS5*** determines the regioselectivity of C–C bond activation.

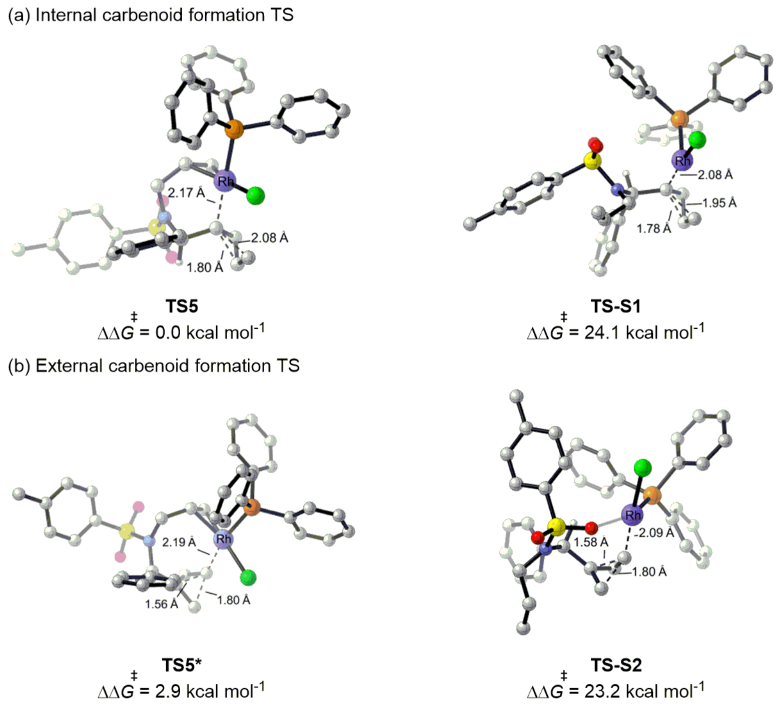

Supplementary Fig. 3. Optimized structures and free energies of alternative rhodium carbenoid formation transition states. TS, transition state.

d. Alternative transition states of alkene carbometalation involved in Rh/PPh_3 case

In addition to **TS8**, **TS-S3** without coordination of the olefin from the allyl group to the rhodium center is also located. As can be seen, **TS8** with olefin coordination is a more favorable transition state for carbometalation.

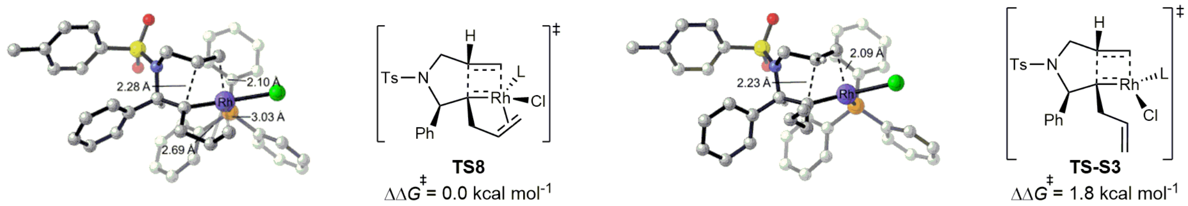

Supplementary Fig. 4. Optimized structures and free energies of alternative carbometalation transition states. L = PPh_3.

e. Alternative transition states of reductive elimination involved in Rh/PPh_3 case

We explored different transition states leading to C–C bond reductive elimination. **TS-S4** without the coordination of the alkene is higher in energy by 2.7 kcal mol^{-1} compared to **TS10**. Therefore, **TS10** is the most favorable transition state to facilitate reductive elimination.

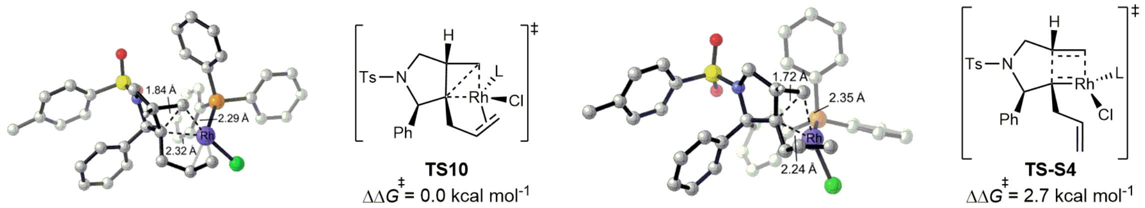

Supplementary Fig. 5. Optimized structures and free energies of alternative transition states of reductive elimination. L = PPh_3.

f. IRC analyses of transition states involved in Rh/PPh3 case

We have performed IRC analysis from both directions, for the transition states that are involved in the Rh/PPh3 case. The computations further verified the C–C bond cleavage and rhodium carbenoid formation (**TS5** and **TS5***), olefin insertion (**TS8**) and reductive elimination (**TS10**) transition states.

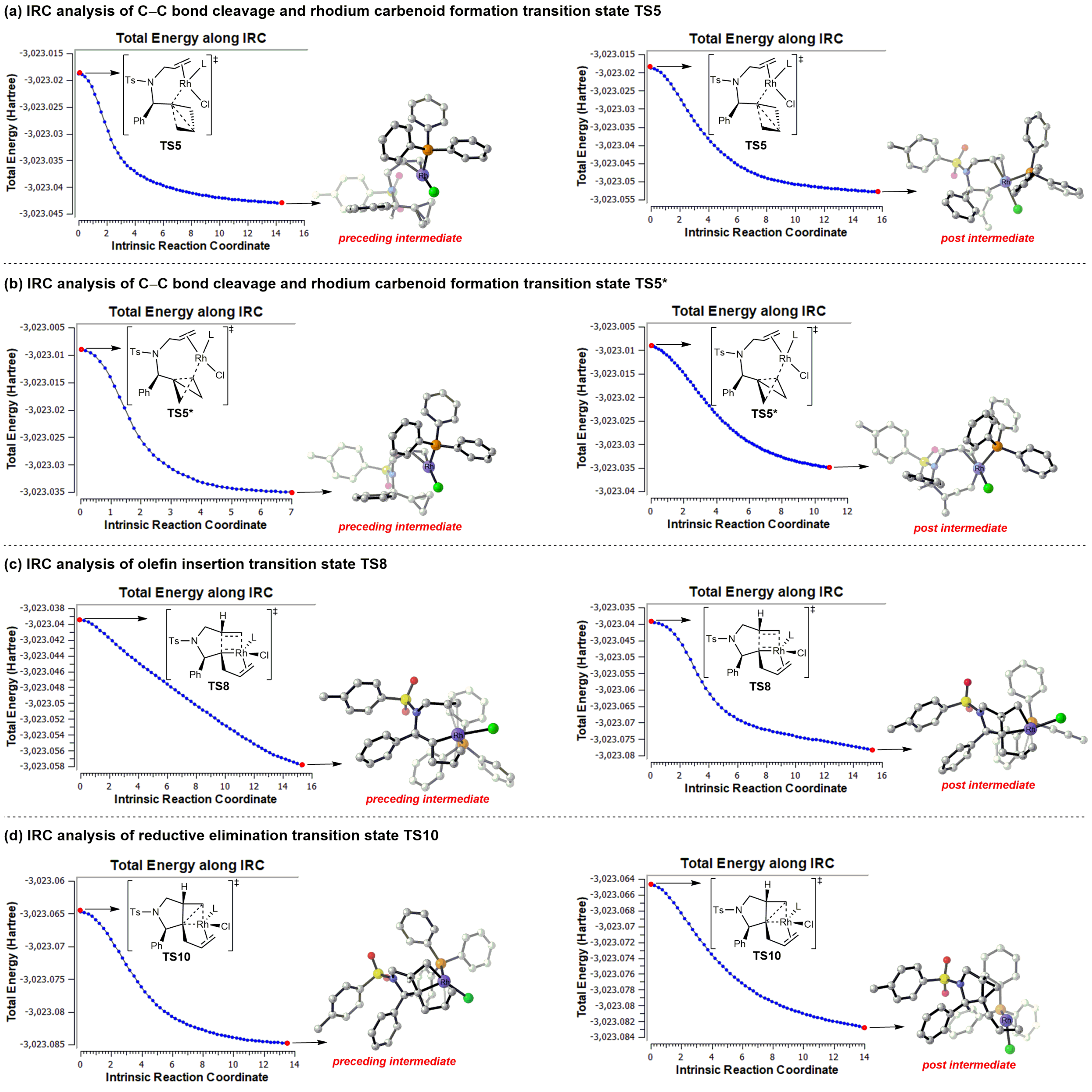

Supplementary Fig. 6. IRC analyses of the transition states in Rh/PPh3 case. (a) **TS5**; (b) **TS5***; (c) **TS8**; (d) **TS10**. L = PPh3.

g. Alternative transition states of *endo* oxidative addition involved in Rh/PPh_3 case

We explored different *endo* oxidative addition transition states. **TS-S5** without the alkene coordination is 5.7 kcal mol^{-1} higher in energy compared to **TS12**. In **TS-S6**, the weaker oxygen coordination replaces the stronger olefin coordination, making it energetically 7.3 kcal mol^{-1} less stable than **TS12**. These results suggest that the most favorable transition state for *endo* oxidative addition is **TS12**.

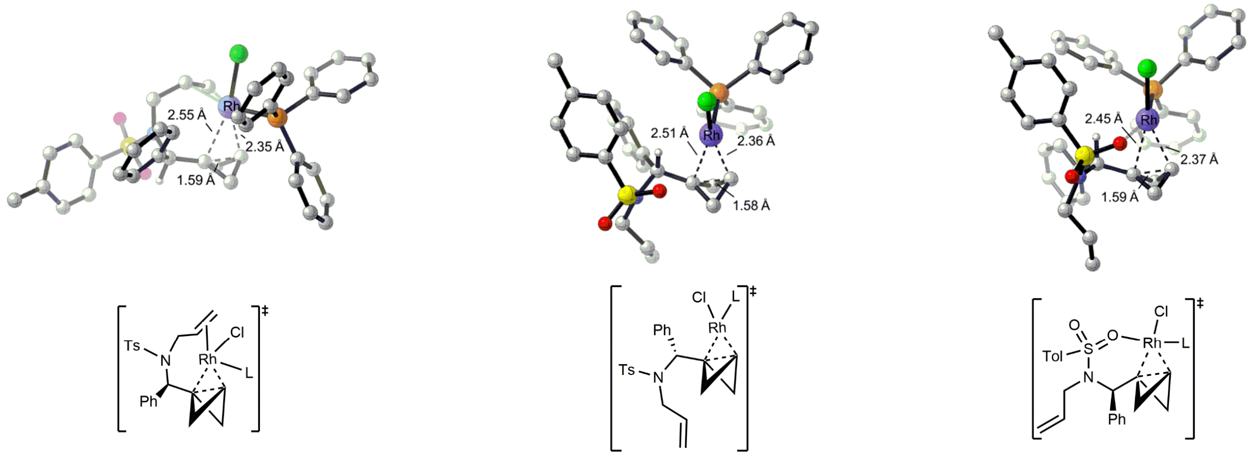

TS12 $\Delta\Delta G^{\ddagger}$ = 0.0 kcal mol^{-1} **TS-S5** $\Delta\Delta G^{\ddagger}$ = 5.7 kcal mol^{-1} **TS-S6** $\Delta\Delta G^{\ddagger}$ = 7.3 kcal mol^{-1}

Supplementary Fig. 7. Optimized structures and free energies of alternative transition states of the *endo* oxidative addition. L = PPh_3.

h. Alternative transition states of ionic species generation

Supplementary Fig. 8 shows the competing transition states for ionic species generation. **TS14** leads to a process in which C(α)–C(β) bond cleavage along with the C(α)–C(γ) bond formation occurs simultaneously. While in **TS-S7**, the C(α)–C(β) bond cleavage and the C(β)–C(γ) bond formation occur at the same time. Both **TS14** and **TS-S7** are transition states of ionic species generation, and the competition between these two transition states determines the bond activation mode. Our calculations suggest that **TS14** is more favorable in terms of Gibbs free energy.

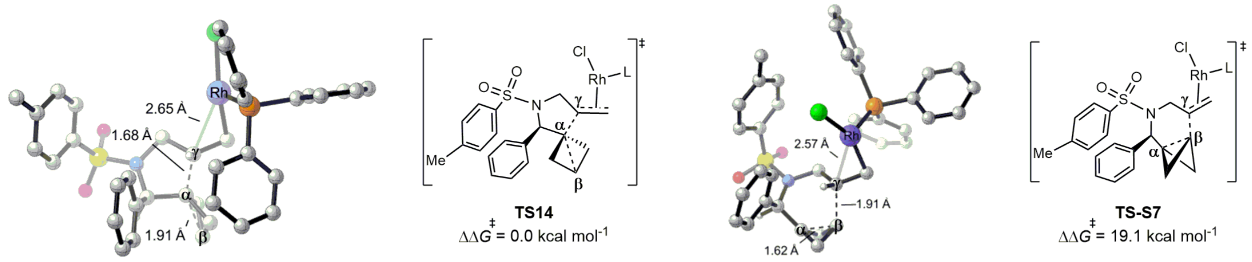

TS14 $\Delta\Delta G^{\ddagger}$ = 0.0 kcal mol^{-1} **TS-S7** $\Delta\Delta G^{\ddagger}$ = 19.1 kcal mol^{-1}

Supplementary Fig. 8. Optimized structures and free energies of alternative ionic species generation transition states. L = PPh_3.

i. Origins of regioselectivity of Rh(I)/PPh_3-catalyzed cycloisomerizations

The Rh(I)/PPh_3-catalyzed cycloisomerizations of BCBs are intramolecular reactions, in which the concerted C–C bond cleavage and rhodium carbenoid formation occur with the assistance of the alkene moiety in the substrate. Based on this scenario, we assumed that the linker (the green-highlighted part in **TS5** and **TS5***, Supplementary Fig. 9a) has a significant effect on regioselectivity: under the influence of the linker, the Rh catalyst tends to attack the adjacent internal carbon via **TS5**, whereas in **TS5***, the Rh catalyst, in order to attack at the external carbon, restricts the conformation of the linker. To support this hyphothesis, we calculated the energy difference of the molecular skeleton highlighted in green in **TS5** and **TS5*** (Supplementary Fig. 9a), by replacing the PPh_3RhCl(BCB) moiety with a hydrogen atom to construct **TS5-model** and **TS5*-model**, respectively (Supplementary Fig. 9b). **TS5-model** is 2.3 kcal mol^{-1} lower in energy than **TS5*-model**, which is similar to the electronic energy difference (3.8 kcal mol^{-1}) of the competitive transition states (**TS5** and **TS5***), indicating that the regioselectivity mainly originates from the difference of molecular skeleton deformation.

To further elucidate the difference in the geometric deformation of the linker in competitive transition states (**TS5** and **TS5***), we conducted analysis based on **TS5-model** and **TS5*-model**. An overlay of these two models was generated first, and root-mean-square deviation (RMSD) value was found to be 0.33 Å (Supplementary Fig. 9c). The overlay structure indicated that the main differences between **TS5-model** and **TS5*-model** stemmed from the phenyl (highlighted with a dashed green oval in Supplementary Fig. 9c) and the linker (highlighted with a dashed red oval in Supplementary Fig. 9c) moieties. Then, we re-optimized **TS5-model** and **TS5*-model**, and both converged to **TS5-model-opt** (Supplementary Fig. 9d). By employing **TS5-model-opt** as the reference structure, we found that the dihedral angles of the highlighted atoms (φ1 and φ2) in the **TS5-model** are similar to those of the optimized structure (**TS5-model-opt**), while the corresponding dihedral angles (φ1 and φ2) in **TS5*-model** are significantly deviated from the optimized values (φ1 and φ2 in **TS5-model-opt**). Therefore, in **TS5-model**, the angle distortion is smaller, and, accordingly, the transition state derived from **TS5-model** has a lower energy, leading to the experimentally observed regioselectivity (Supplementary Fig. 9e).

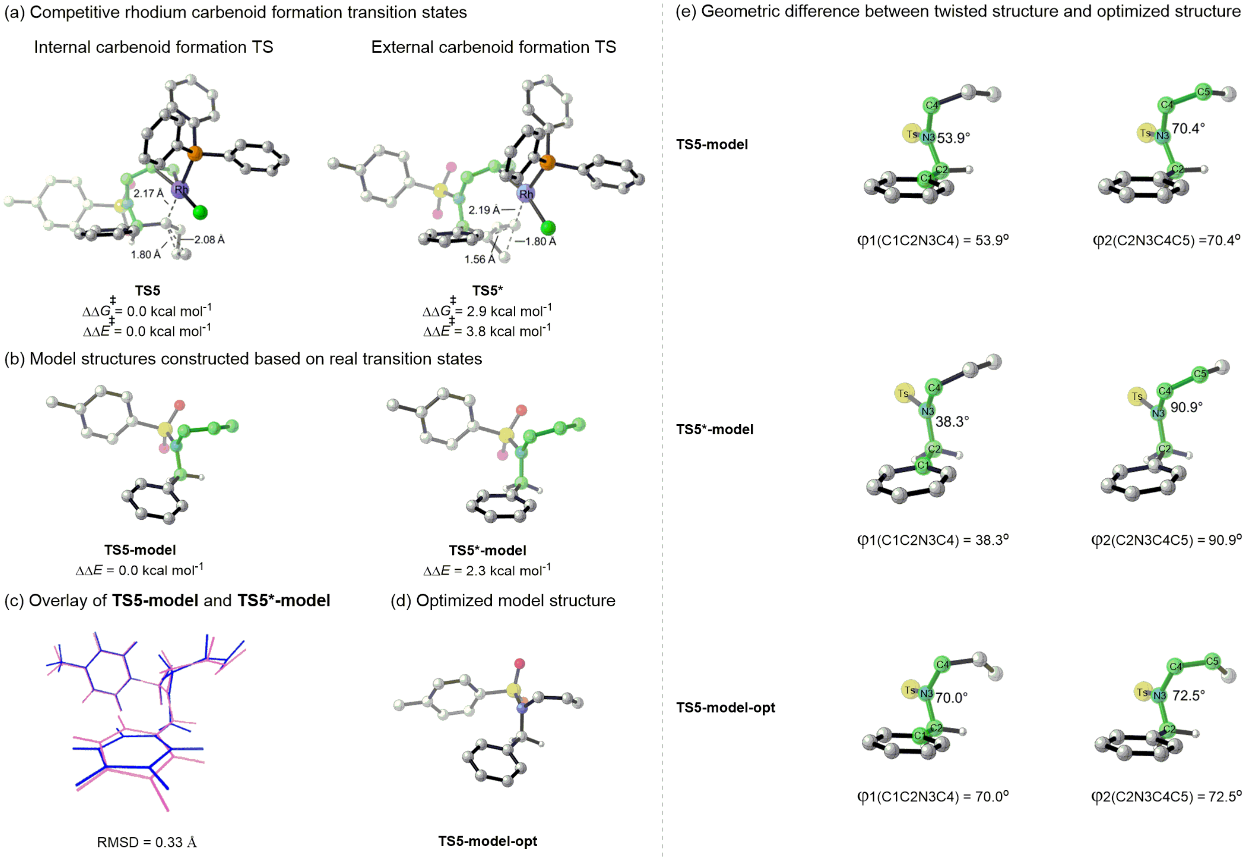

Supplementary Fig. 9. Origins of regioselectivity for Rh(I)/PPh_3-catalyzed cycloisomerizations. (a) DFT-optimized structures of rhodium carbenoid formation transition states. TS, transition state. (b) Model structures constructed based on **TS5** and **TS5***. (c) Overlay of **TS5-model** and **TS5*-model**. (d) Optimized structure (**TS5-model-opt**) based on **TS5-model** or **TS5*-model**. (e) Comparison of optimized and distorted structures. φ, dihedral angle.

j. Origins of diastereoselectivity of Rh(I)/PPh_3-catalyzed cycloisomerizations

Comparing the two olefin insertion transition states (**TS8** and **TS8***, Supplementary Fig. 10a), we posed that in **TS8**, the allylic alkene coordination to rhodium provided additional stabilization, thus rendering this transition state energetically more favorable than **TS8***. To support this hypothesis, we replaced the allyl group with a methyl substituent in **TS8-model** and **TS8*-model**, respectively. As shown in Supplementary Fig. 10b, once we remove the alkene coordination, **TS8*-model** is actually more stable than **TS8-model** by 1.2 kcal mol^{-1} in terms of electronic energy. This reversal highlighted the importance of the rhodium-alkene coordination, consistent with our hypothesis.

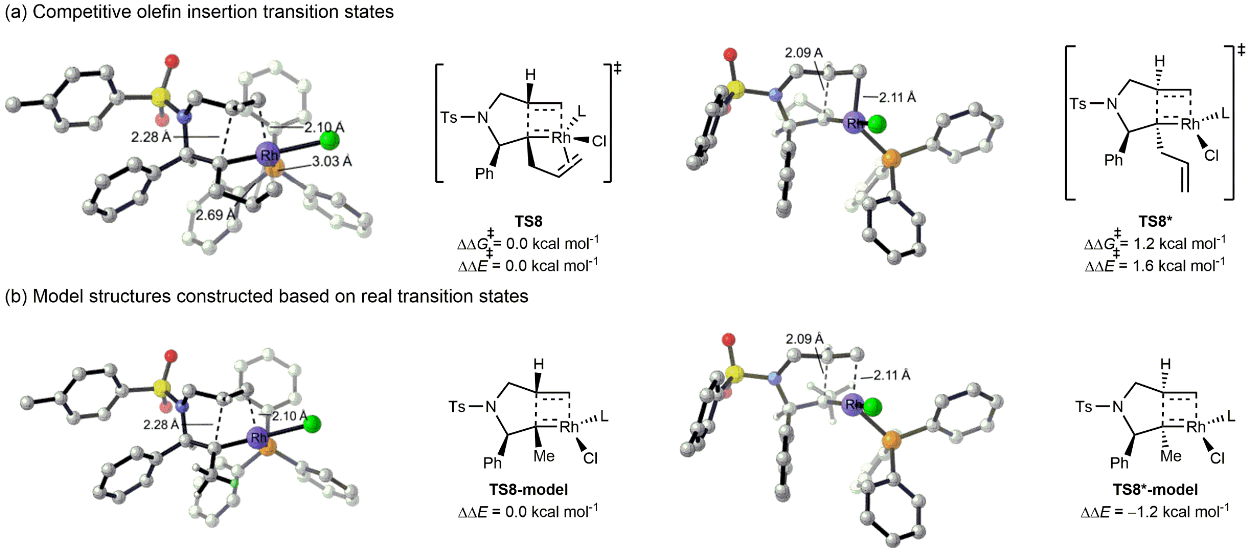

Supplementary Fig. 10. Origins of diastereoselectivity in Rh(I)/PPh_3-catalyzed cycloisomerizations. L = PPh_3. (a) DFT-optimized structures of olefin insertion transition states. (b) Model structures constructed based on **TS8** and **TS8***.

k. DFT-optimized structures of selected intermediates and transition states involved in Rh(I)/dppe catalysis

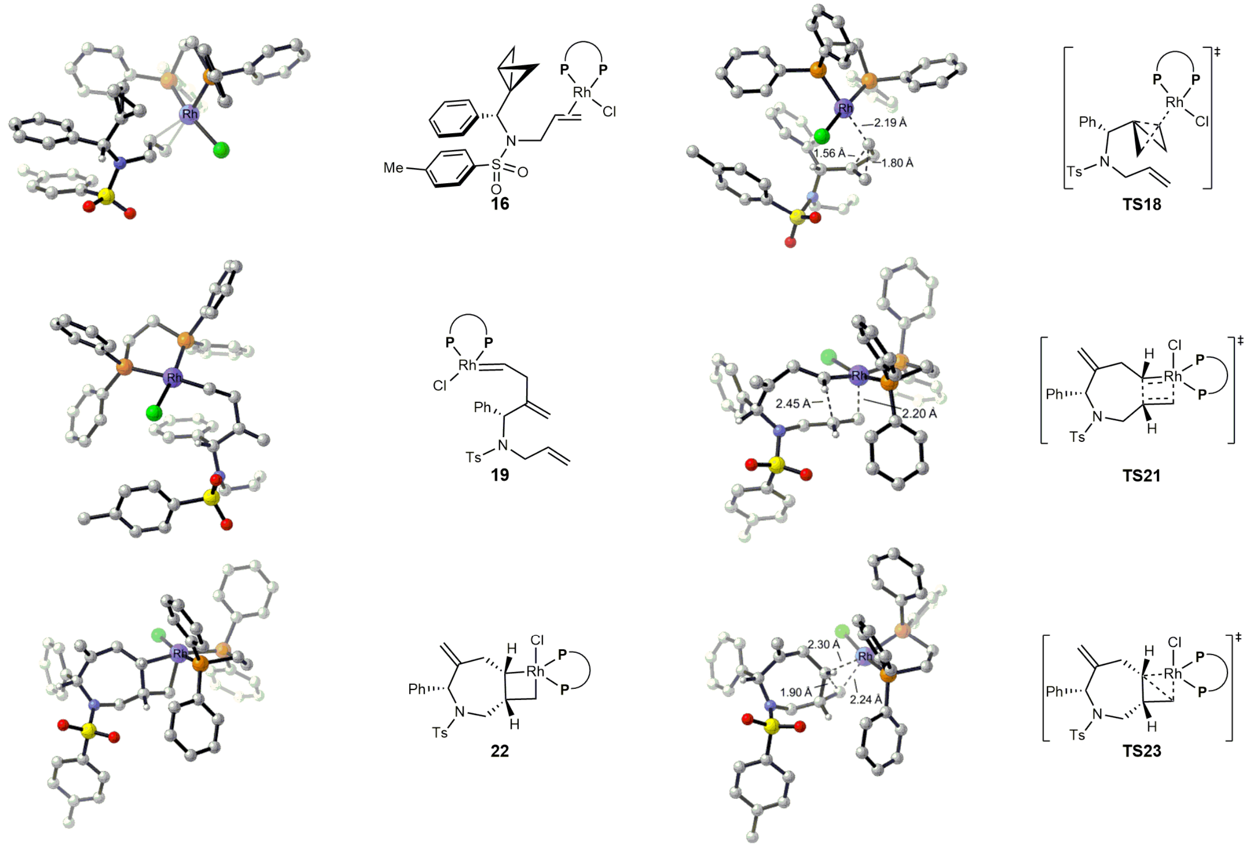

Supplementary Fig. 11. DFT-optimized structures of selected intermediates and transition states involved in Rh(I)/dppe-catalyzed cycloisomerizations.

l. Alternative transition states of central C–C bond cleavage involved in Rh/dppe case

A number of mechanistic pathways were considered for central C–C bond cleavage. **TS-S8** is the *endo* oxidative addition transition state to break the central C–C bond, and it is higher in erergy than **TS18** by 4.6 kcal mol^{-1}. **TS-S9**, **TS-S10**, **TS-S11** and **TS-S12** are concerted transition states with alkene assistance to form rhodium carbenoids. **TS-S9** and **TS-S11** lead to the internal carbenoid formation, and **TS-S10** and **TS-S12** lead to the external carbenoid formation. In comparison to **TS18**, **TS-S9**, **TS-S10**, **TS-S11** and **TS-S12** are disfavored by 10.6 kcal mol^{-1}, 7.3 kcal mol^{-1}, 11.4 kcal mol^{-1} and 15.5 kcal mol^{-1}, respectively. Therefore, the competition between **TS18** and **TS18*** (Fig. 8a) determines the regioselectivity of the cycloisomerizations.

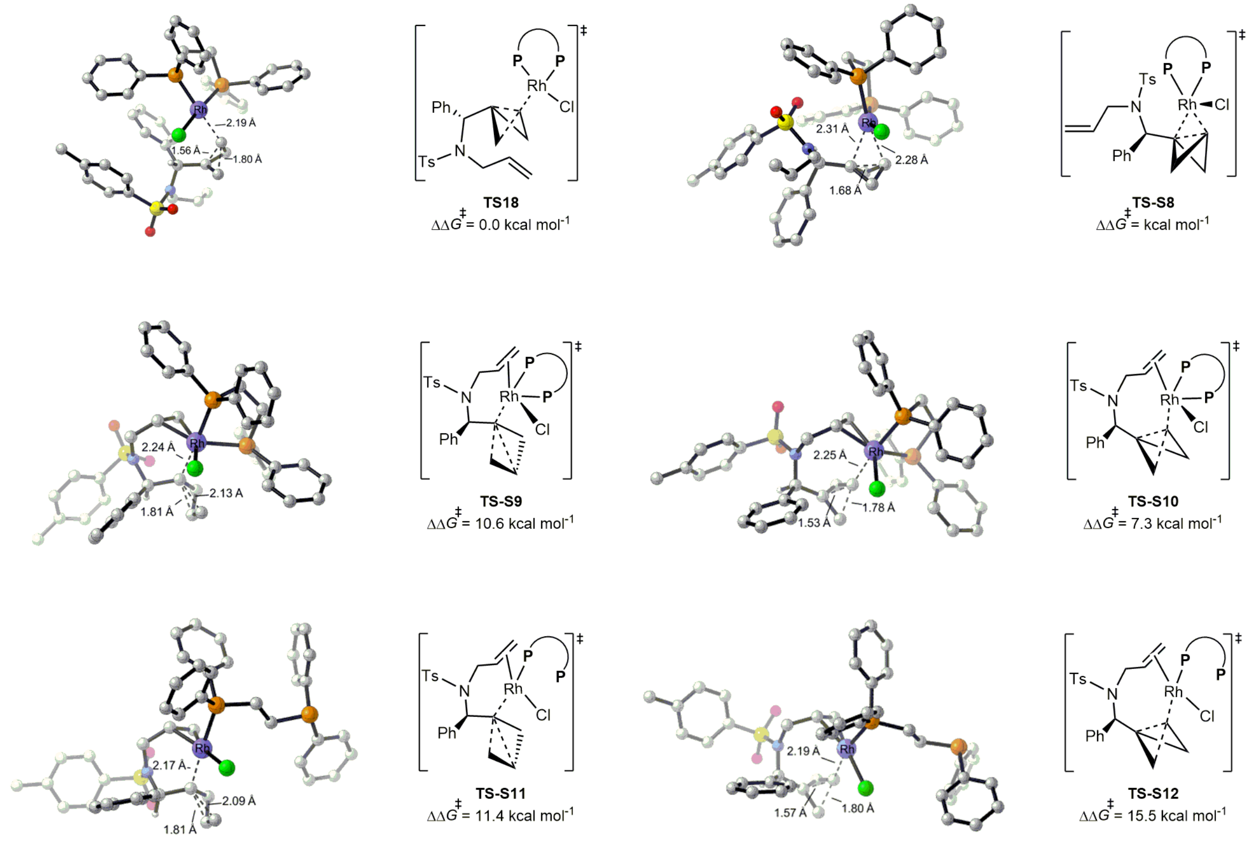

Supplementary Fig. 12. Optimized structures and free energies of alternative C–C bond cleavage transition states.

m. IRC analyses of transition states involved in Rh/dppe case

We have performed IRC analysis from both directions, for the transition states that are involved in Rh/dppe case. The computations further verified the C–C bond cleavage and rhodium carbenoid formation (**TS18** and **TS18***), olefin insertion (**TS21**) and reductive elimination (**TS23**) transition states.

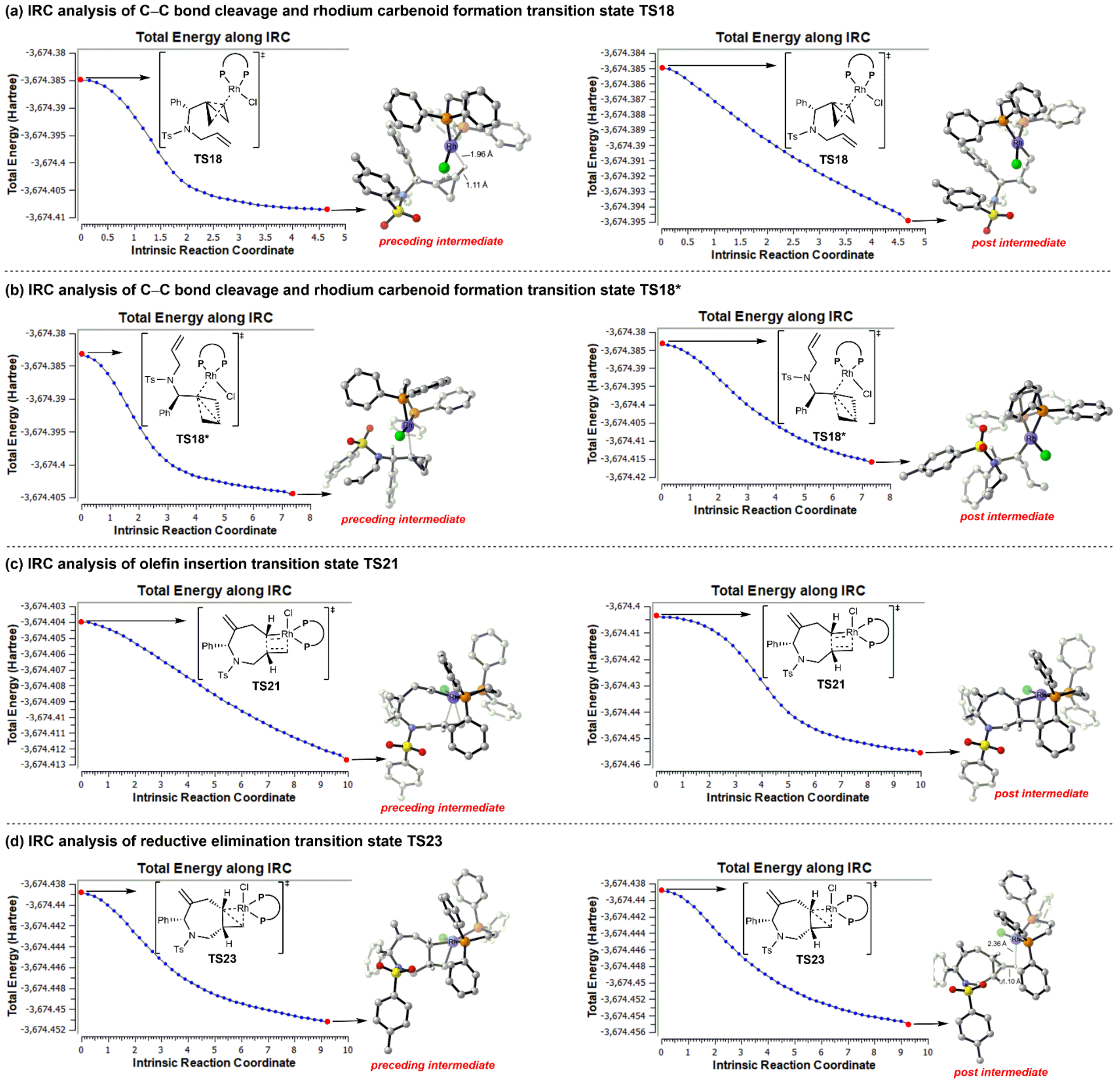

Supplementary Fig. 13. IRC analyses of the transition states in Rh/PPh_3 case. (a) **TS18**; (b) **TS18***; (c) **TS21**; (d) **TS23**.

n. Origins of regioselectivity for Rh(I)/dppe-catalyzed cycloisomerizations

In addition to the analysis shown in the manuscript, we performed additional calculations (Supplementary Fig. 14) to further support our rationalization: furthermore, if a smaller substituent such as hydrogen is used to replace the phenyl group in the substrate to construct **TS18-model** and **TS18*-model**, the energies of **TS18-model** and **TS18*-model** are similar, consistent with our rationalization (Supplementary Fig. 14b).

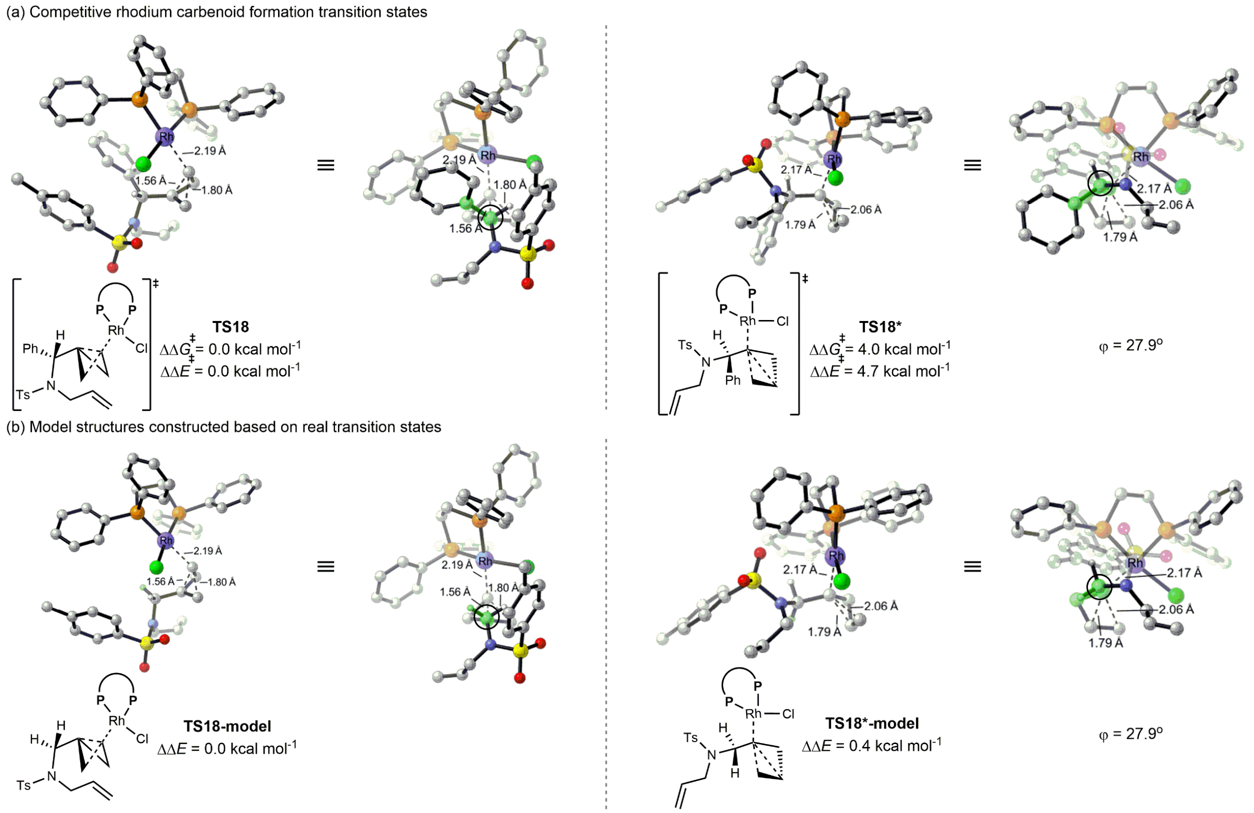

Supplementary Fig. 14. Origins of regioselectivity for Rh(I)/dppe-catalyzed cycloisomerizations. (a) DFT-optimized structures of rhodium carbenoid formation transition states. (b) Model structures constructed based on **TS18** and **TS18***. φ, dihedral angle.

o. Origins of diastereoselectivity for Rh(I)/dppe-catalyzed cycloisomerizations

The competition between **TS21** and **TS21*** determines the regioselectivity for Rh(I)/dppe-catalyzed cycloisomerizations. **TS21** is 8.3 kcal mol^{-1} more stable than **TS21***, indicating that alkene carbometalation exclusively proceeds through **TS21**, forming the experimentally observed product (Supplementary Fig. 15a).

The origins of this diastereoselectivity were further investigated (Supplementary Fig. 15). To understand which part of the complex is responsible for the selectivity, we first removed the chloride and phosphine ligands from rhodium to construct **TS21-model-I** and **TS21*-model-I**, respectively. The electronic energy difference ($\Delta\Delta E$=9.5 kcal mol^{-1}) between these two models is

similar to that of the competing transition states ($\Delta\Delta E$=8.3 kcal mol^{-1}), indicating that the ligands have limited effects on the selectivity (Supplementary Fig. 15b). Then, we removed the rhodium to construct **TS21-model-II** and **TS21*-model-II**, respectively. The energy difference of model II ($\Delta\Delta E$=9.5 kcal mol^{-1}) is identical to that of model I ($\Delta\Delta E$=9.5 kcal mol^{-1}), suggesting that the metallocyclobutane has no effect on the energy difference (Supplementary Fig. 15c).

Moreover, we replaced the phenyl and tosyl groups with hydrogens to construct **TS21-model-III** and **TS21*-model-III**, respectively. The energy difference of model III ($\Delta\Delta E$=9.1 kcal mol^{-1}) is similar to that of model II ($\Delta\Delta E$=9.5 kcal mol^{-1}). The above analysis indicated that the energy difference of the competing transition states mainly results from the ring strain of the seven-membered ring, which is larger in **TS21***. The reason behind this is that in **TS21**, the seven-membered ring can assume a pseudo-chair conformation, while in **TS21***, the seven-membered ring needs to form a pseudo-boat conformation to accommodate the bond formation process, leading to the overall selectivity (Supplementary Fig. 15d).

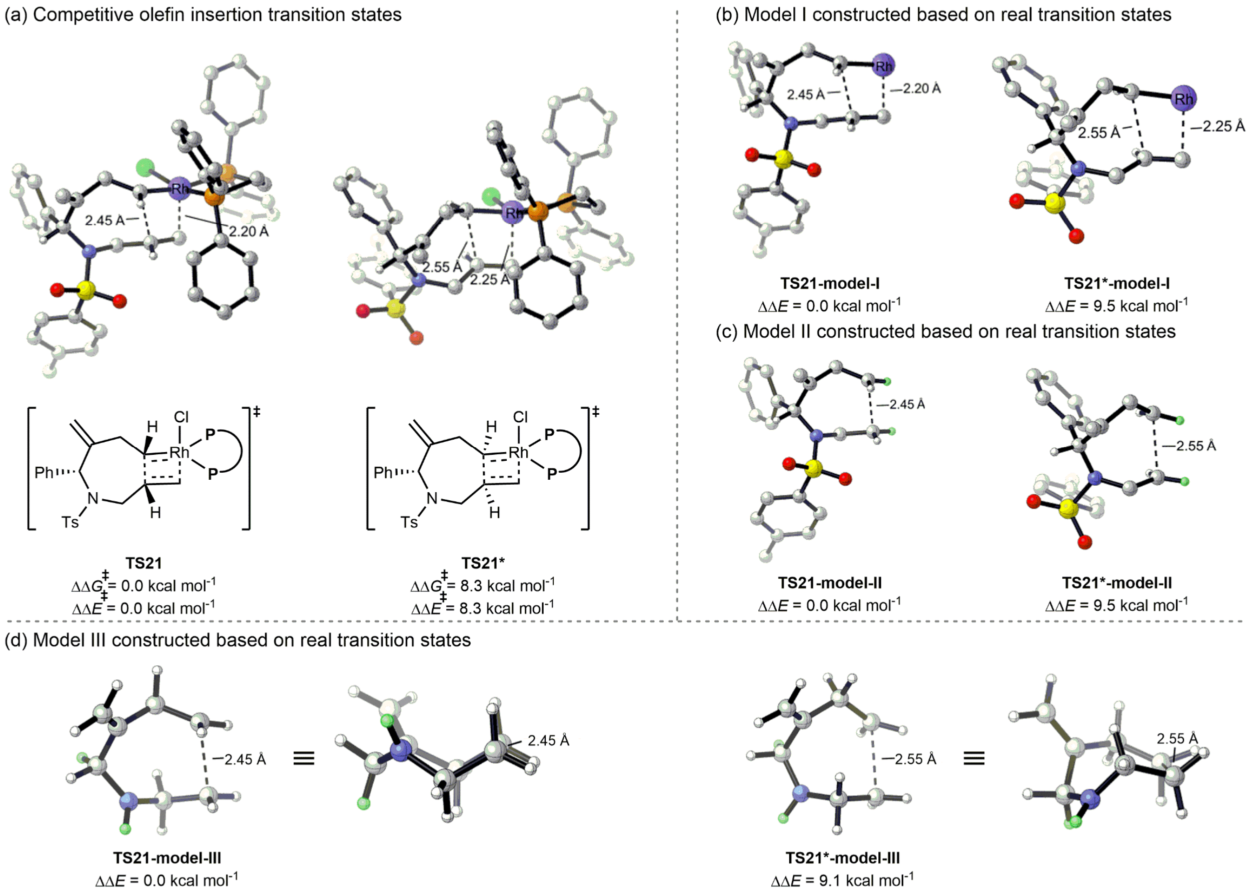

Supplementary Fig. 15. Origins of diastereoselectivity in Rh(I)/dppe-catalyzed cycloisomerizations. (a) DFT-optimized structures of olefin insertion transition states. (b) **Model I**, (c) **Model II** and (d) **Model III** constructed based on **TS21** and **TS21***. From **Model I** to **Model III**, the model structure is gradually simplified, which is conducive to revealing the determinants that control selectivity.

III. Supplementary Tables

Supplementary Table 2. Energies in **Fig. 4**, **Fig. 5**, **Fig. 6**, **Fig. 7**, **Fig. 8**, **Supplementary Fig. 2**, **Supplementary Fig. 3**, **Supplementary Fig. 4**, **Supplementary Fig. 5**, **Supplementary Fig. 7**, **Supplementary Fig. 8**, **Supplementary Fig. 12**. Zero-point vibrational energy (*ZPVE*), thermal correction to enthalpy (*TCH*), thermal correction to Gibbs free energy (*TCG*), energies (*E*), enthalpies (*H*), and Gibbs free energies (*G*) (in Hartree) of the structures calculated at the PBE0-D3/def2-TZVPP-CPCM(toluene)//B3LYP-D3/def2-SVP level of theory.

Structures	*ZPVE*	*TCH*	*TCG*	*E*	*H*	*G*	Imaginary Frequency
**(*R*)-1**	0.392889	0.417578	0.337037	-1416.454531	-1416.036953	-1416.117494	
**2**	0.395445	0.419203	0.341535	-1416.518558	-1416.099355	-1416.177023	
**3**	0.396974	0.420132	0.343340	-1416.513767	-1416.093635	-1416.170427	
**4**	0.673218	0.717477	0.592546	-3022.851639	-3022.134162	-3022.259093	
**TS5**	0.671869	0.715920	0.590278	-3022.813059	-3022.097139	-3022.222781	376.5*i*
**6**	0.671362	0.716628	0.587245	-3022.838599	-3022.121971	-3022.251354	
**7**	0.671748	0.715924	0.593111	-3022.855863	-3022.139939	-3022.262752	
**TS8**	0.670203	0.714977	0.588016	-3022.830881	-3022.115904	-3022.242865	235.4*i*
**9**	0.674555	0.718210	0.596892	-3022.882405	-3022.164195	-3022.285513	
**TS10**	0.673790	0.716995	0.596028	-3022.861018	-3022.144023	-3022.264990	250.0*i*
**11**	0.674878	0.718849	0.592950	-3022.889324	-3022.170475	-3022.296374	
**TS12**	0.671530	0.715234	0.591678	-3022.796055	-3022.080821	-3022.204377	192.7*i*
**13**	0.671894	0.716043	0.592491	-3022.821734	-3022.105691	-3022.229243	
**TS14**	0.670476	0.714636	0.587799	-3022.771092	-3022.056456	-3022.183293	114.4*i*
**15**	0.670866	0.715554	0.587200	-3022.769123	-3022.053569	-3022.181923	
**TS5***	0.670469	0.714466	0.589286	-3022.807499	-3022.093033	-3022.218213	439.2*i*
**TS8***	0.670093	0.714938	0.587629	-3022.828528	-3022.113590	-3022.240899	170.1*i*
**16**	0.822063	0.875729	0.728783	-3674.156524	-3673.280795	-3673.427741	
**17**	0.822102	0.875916	0.730889	-3674.131585	-3673.255669	-3673.400696	
**TS18**	0.819459	0.873004	0.729465	-3674.108250	-3673.235246	-3673.378785	489.1*i*
**19**	0.820656	0.874955	0.728244	-3674.123885	-3673.248930	-3673.395641	
**20**	0.821757	0.875108	0.729610	-3674.136114	-3673.261006	-3673.406504	
**TS21**	0.820326	0.873390	0.727138	-3674.125828	-3673.252438	-3673.398690	171.4*i*
**22**	0.820326	0.873390	0.727138	-3674.184141	-3673.310751	-3673.457003	
**TS23**	0.823401	0.875920	0.730425	-3674.166630	-3673.290710	-3673.436205	269.8*i*
**24**	0.824647	0.877999	0.728003	-3674.186818	-3673.308819	-3673.458815	
**TS18***	0.819995	0.873792	0.728803	-3674.101188	-3673.227396	-3673.372385	414.0*i*
**TS21***	0.820433	0.873567	0.727442	-3674.112919	-3673.239352	-3673.385477	145.5*i*
**Rh-I**	0.221017	0.239455	0.173968	-1455.598973	-1455.359518	-1455.425005	
**Ph_3P**	0.273817	0.290572	0.227571	-1035.614974	-1035.324402	-1035.387403	
**$CH_2=CH_2$**	0.050685	0.054676	0.029141	-78.514415	-78.459739	-78.485274	
**Rh-II**	0.037541	0.053624	-0.009078	-1594.535989	-1594.482365	-1594.545067	
**dppe**	0.422673	0.448739	0.362614	-1686.877201	-1686.428462	-1686.514587	

**CO**	0.005114	0.008418	-0.014012	-113.231227	-113.222809	-113.245239	
**TS-S1**	0.668897	0.714499	0.585175	-3022.769526	-3022.055027	-3022.184351	410.9*i*
**TS-S2**	0.668112	0.713192	0.587501	-3022.773302	-3022.060110	-3022.185801	669.6*i*
**TS-S3**	0.670795	0.715447	0.587812	-3022.827753	-3022.112306	-3022.239941	235.3*i*
**TS-S4**	0.672413	0.716463	0.592051	-3022.852714	-3022.136251	-3021.544200	141.8*i*
**TS-S5**	0.670962	0.715328	0.592546	-3022.787780	-3022.072452	-3022.195234	195.9*i*
**TS-S6**	0.670935	0.715297	0.591172	-3022.783941	-3022.068644	-3022.192769	188.9*i*
**TS-S7**	0.670825	0.714440	0.593240	-3022.746151	-3022.031711	-3022.152911	673.4*i*
**TS-S8**	0.821361	0.874272	0.733010	-3674.104406	-3673.230134	-3673.371396	206.0*i*
**TS-S9**	0.822149	0.874841	0.732721	-3674.094564	-3673.219723	-3673.361843	298.1*i*
**TS-S10**	0.819590	0.872555	0.728974	-3674.096197	-3673.223642	-3673.367223	527.1*i*
**TS-S11**	0.820723	0.874224	0.723540	-3674.084083	-3673.209859	-3673.360543	377.4*i*
**TS-S12**	0.819239	0.872784	0.723169	-3674.077296	-3673.204512	-3673.354127	430.9*i*

Supplementary Table 3. Energies in **Supplementary Table 1**. Zero-point vibrational energy (*ZPVE*), thermal correction to enthalpy (*TCH*), thermal correction to Gibbs free energy (*TCG*), energies (*E*), enthalpies (*H*), and Gibbs free energies (*G*) (in Hartree) of the structures calculated at Method/def2-TZVPP-CPCM(toluene)//B3LYP-D3/def2-SVP level of theory.

**Method**	**Structures**	***ZPVE***	***TCH***	***TCG***	***E***	***H***	***G***	**Imaginary Frequency**
**M06**	**TS5**	0.671869	0.715920	0.590278	-3023.864040	-3023.148120	-3023.273762	376.5*i*
	**TS5***	0.670469	0.714466	0.589286	-3023.861867	-3023.147401	-3023.272581	439.2*i*
	**TS18***	0.819995	0.873792	0.728803	-3675.376356	-3674.502564	-3674.647553	414.0*i*
	**TS18**	0.819459	0.873004	0.729465	-3675.388416	-3674.515412	-3674.658951	489.1*i*
**M06-2X**	**TS5**	0.671869	0.715920	0.590278	-3024.317676	-3023.601756	-3023.727398	376.5*i*
	**TS5***	0.670469	0.714466	0.589286	-3024.312977	-3023.598511	-3023.723691	439.2*i*
	**TS18***	0.819995	0.873792	0.728803	-3675.942009	-3675.068217	-3675.213206	414.0*i*
	**TS18**	0.819459	0.873004	0.729465	-3675.948609	-3675.075605	-3675.219144	489.1*i*
**ωB97X-D**	**TS5**	0.671869	0.715920	0.590278	-3024.656670	-3023.940750	-3024.066392	376.5*i*
	**TS5***	0.670469	0.714466	0.589286	-3024.649737	-3023.935271	-3024.060451	439.2*i*
	**TS18***	0.819995	0.873792	0.728803	-3676.333911	-3675.460119	-3675.605108	414.0*i*
	**TS18**	0.819459	0.873004	0.729465	-3676.341714	-3675.468710	-3675.612249	489.1*i*
**B3LYP-D3**	**TS5**	0.671869	0.715920	0.590278	-3025.401689	-3024.685769	-3024.811411	376.5*i*
	**TS5***	0.670469	0.714466	0.589286	-3025.391409	-3024.676943	-3024.802123	439.2*i*
	**TS18***	0.819995	0.873792	0.728803	-3677.224779	-3676.350987	-3676.495976	414.0*i*
	**TS18**	0.819459	0.873004	0.729465	-3677.226730	-3676.353726	-3676.497265	489.1*i*

Supplementary Table 4. Energies in **Supplementary Fig. 9**, **Supplementary Fig. 10**, **Supplementary Fig. 14**, **Supplementary Fig. 15**. Energies (*E*) (in Hartree) of the structures calculated at the PBE0-D3/def2-TZVPP//B3LYP-D3/def2-SVP level of theory.

Structures	*E*
**TS5**	-3022.799549
**TS5***	-3022.793507
**TS5-model**	-1261.784876
**TS5*-model**	-1261.781230
**TS8**	-3022.815570
**TS8***	-3022.813000
**TS8-model**	-2945.482915
**TS8*-model**	-2945.484749
**TS18**	-3674.093370
**TS18***	-3674.085846
**TS18-model**	-3443.216316
**TS18*-model**	-3443.215694
**TS21**	-3674.110371
**TS21***	-3674.097141
**TS21-model-I**	-1526.751402
**TS21*-model-I**	-1526.736261
**TS21-model-II**	-1378.327066
**TS21*-model-II**	-1378.311953
**TS21-model-III**	-328.886403
**TS21*-model-III**	-328.871927

IV. Supplementary References

1. Frisch, M. J., Trucks, G. W., Schlegel, H. B., Scuseria, G. E., Robb, M. A., Cheeseman, J. R., Scalmani, G., Barone, V., Petersson, G. A., Nakatsuji, H., Li, X., Caricato, M., Marenich, A. V., Bloino, J., Janesko, B. G., Gomperts, R., Mennucci, B., Hratchian, H. P., Ortiz, J. V., Izmaylov, A. F., Sonnenberg, J. L., Williams-Young, D., Ding, F., Lipparini, F., Egidi, F., Goings, J., Peng, B., Petrone, A., Henderson, T., Ranasinghe, D., Zakrzewski, V. G., Gao, J., Rega, N., Zheng, G., Liang, W., Hada, M., Ehara, M., Toyota, K., Fukuda, R., Hasegawa, J., Ishida, M., Nakajima, T., Honda, Y., Kitao, O., Nakai, H., Vreven, T., Throssell, K., Montgomery Jr. J. A., Peralta, J. E., Ogliaro, F., Bearpark, M. J., Heyd, J. J., Brothers, E. N., Kudin, K. N., Staroverov, V. N., Keith, T. A., Kobayashi, R., Normand, J., Raghavachari, K., Rendell, A. P., Burant, J. C., Iyengar, S. S., Tomasi, J., Cossi, M., Millam, J. M., Klene, M., Adamo, C., Cammi, R., Ochterski, J. W., Martin, R. L., Morokuma, K., Farkas, O., Foresman, J. B., Fox, D. J. Gaussian 16, Rev. A.03, Wallingford, CT, 2016.

2. (a) Becke, A. D. Density functional thermochemistry. III. The role of exact exchange. *J. Chem. Phys*. **98**, 5648−5652 (1993). (b) Lee, C., Yang, W. & Parr, R. G. Development of the Colle-Salvetti correlation-energy formula into a functional of the electron density. *Phys. Rev. B*. **37**, 785−789 (1988).

3. Weigend, F. & Ahlrichs, R. Balanced basis sets of split valence, triple zeta valence and quadruple zeta valence quality for H to Rn: design and assessment of accuracy. *Phys. Chem. Chem. Phys*. **7**, 3297−3305 (2005).

4. Grimme, S., Antony, J., Ehrlich, S. & Krieg, H. A consistent and accurate Ab initio parametrization of density functional dispersion correction (DFT-D) for the 94 elements H-Pu. *J. Chem. Phys*. **132**, 154104 (2010).
5. Adamo, C. & Barone, V. Toward reliable density functional methods without adjustable parameters: The PBE0 model. *J. Chem. Phys*. **110**, 6158−69 (1999).
6. Weigend, F. Accurate Coulomb-fitting basis sets for H to Rn. *Phys. Chem. Chem. Phys*. **8**, 1057−1065 (2006).
7. Cossi, M., Rega, N., Scalmani, G. & Barone, V. Energies, structures, and electronic properties of molecules in solution with the C-PCM solvation model. *J. Comput. Chem*. **24**, 669−681 (2003).
8. Legault, C. Y. *CYLview*; version 1.0b; Universitéde Sherbrooke: 2009 (http://www.cylview.org).
9. (a) Kelly, C. P., Cramer, C. J. & Truhlar, D. G. SM6: A density functional theory continuum solvation model for calculating aqueous solvation free energies of neutrals, ions, and solute-water clusters. *J. Chem. Teory Comput*. **1**, 1133–1152 (2005). (b) Kelly, C. P., Cramer, C. J. & Truhlar, D. G. Aqueous solvation free energies of ions and ion−water clusters based on an accurate value for the absolute aqueous solvation free energy of the proton. *J. Phys. Chem. B*. **110**, 16066–16081 (2006).
10. Dohm, S., Hansen, A., Steinmetz, M., Grimme, S. & Checinski, M. P. Comprehensive thermochemical benchmark set of realistic closed-shell metal organic reactions. *J. Chem. Theory Comput*. **14**, 2596–2608 (2018). In this paper, reactions containing rhodium have been chosen as part of benchmark set.
11. Husch, T., Freitag, L. & Reiher, M. Calculation of ligand dissociation energies in large transition-metal complexes. *J. Chem. Theory Comput*. **14**, 2456–2468 (2018).
12. Steinmetz, M. & Grimme, S. Benchmark study of the performance of density functional theory for bond activations with (Ni,Pd)-based transition-metal catalysts. *ChemistryOpen*. **2**, 115−124 (2013).
13. Walczak, M. A. A. & Wipf, P. Rhodium(I)-catalyzed cycloisomerizations of bicyclobutanes. *J. Am. Chem. Soc*. **130**, 6924−6925 (2008).
14. (a) Zhao, Y. & Truhlar, D. G. The M06 suite of density functionals for main group thermochemistry, thermochemical kinetics, noncovalent interactions, excited states, and transition elements: two new functionals and systematic testing of four M06-class functionals and 12 other functionals. *Theor. Chem. Acc*. **120**, 215–241 (2008). (b) Zhao, Y. & Truhlar, D. G. Density functionals with broad applicability in chemistry. *Acc. Chem. Res*. **41**, 157–167 (2008).
15. Chai, J.-D. & Head-Gordon, M. Long-range corrected hybrid density functionals with damped atom–atom dispersion corrections. *Phys. Chem. Chem. Phys*. **10**, 6615−6620 (2008).
