# Supplementary material for: How mono- and diphosphine ligands alter regioselectivity of the Rh-catalyzed annulative cleavage of bicyclo[1.1.0]butanes
Source: Nat Commun. 2022 Nov 26;13:7292. doi: 10.1038/s41467-022-34837-x (PMC9701199; doi:10.1038/s41467-022-34837-x)
Supplement: Supplementary file 4 — Supplementary Data 1 [file 41467_2022_34837_MOESM4_ESM.docx]

**How Mono- and Diphosphine Ligands Alter Regioselectivity of the Rh-Catalyzed Annulative Cleavage of Bicyclo[1.1.0]butanes**

Pan-Pan Chen^1^, Peter Wipf^2*^, and K. N. Houk^1*^

^1^Department of Chemistry and Biochemistry, University of California, Los Angeles, California 90095, United States

^2^Department of Chemistry, University of Pittsburgh, 219 Parkman Avenue, Pittsburgh, Pennsylvania 15260, United States

*Corresponding authors: houk@chem.ucla.edu; pwipf@pitt.edu

**Cartesian Coordinates of Calculated Structures**

**1**

C -3.99568100 -0.51367900 -0.39183700

C -3.30091800 -1.51489100 -1.08896000

C -2.05460000 -1.96858900 -0.65079500

C -1.49506300 -1.40728400 0.49891500

C -2.17825100 -0.43149900 1.23074500

C -3.42059800 0.00733800 0.78029800

H -3.74331400 -1.94999900 -1.98927100

H -1.52204100 -2.75820300 -1.18402900

H -1.72523500 -0.01839300 2.13234900

H -3.95096600 0.78045900 1.34265600

C -5.32044200 0.01192500 -0.88384300

H -5.22034700 1.05758100 -1.22219300

H -5.70961100 -0.57858300 -1.72617400

H -6.07638600 0.00479200 -0.08201500

S 0.17960700 -1.84268500 0.96815900

O 0.43860900 -3.20315300 0.48573500

O 0.34684000 -1.46658800 2.37513300

N 1.16469300 -0.83257300 0.06383700

C 1.69403900 -1.31830300 -1.21822900

H 0.93914600 -1.97198300 -1.68511000

H 1.82774700 -0.45558600 -1.88608700

C 2.98683900 -2.07605800 -1.06731800

H 2.93624700 -2.91901400 -0.36985200

C 4.11369700 -1.78560100 -1.72061500

H 4.17141200 -0.93817900 -2.41271700

H 5.02233300 -2.37981700 -1.58916800

C 1.38261300 0.54703300 0.53381000

H 1.42367200 0.49142900 1.63172700

C 0.25305100 1.50351800 0.15574500

C -0.07392000 2.55523500 1.02319000

C -0.47430700 1.36316400 -1.03402000

C -1.10557500 3.44591000 0.71305300

H 0.47601500 2.66605000 1.96226500

C -1.50798200 2.24886900 -1.34675200

H -0.26149700 0.53126200 -1.70638100

C -1.82792700 3.29400900 -0.47455600

H -1.35267500 4.25437800 1.40604200

H -2.07459400 2.11313500 -2.27140200

H -2.64079700 3.98357400 -0.71605700

C 2.72284800 1.05899600 0.08476900

C 4.04034800 1.01285800 0.79630600

C 3.10935600 1.97407300 -1.04134500

C 3.43721000 2.32230800 0.37982200

H 4.92560900 0.62209500 0.27233800

H 4.00005400 0.76313700 1.86336700

H 3.91575400 1.67661400 -1.73005600

H 2.29332000 2.53631800 -1.50921100

H 3.12757100 3.18182900 0.97433500

**2**

C -0.97143600 -2.03043800 1.34983400

C -2.36084200 -1.52362100 1.68675200

C -2.74361700 -0.45884600 0.67748500

C -1.57331000 -0.31618700 -0.31311100

H -0.88966600 -3.12724900 1.34439600

H -0.22259400 -1.63378700 2.06338900

H -2.63574000 -1.42557500 2.74062500

H -1.92901300 -0.39326700 -1.35060200

C -0.79395600 0.98237700 -0.16568700

C -0.70506200 1.87245000 -1.24231900

C -0.12556700 1.30042500 1.02751100

C 0.03434500 3.05506000 -1.13557100

H -1.21404400 1.62903200 -2.17807100

C 0.61853000 2.47570000 1.13697200

H -0.17925000 0.61818300 1.87769300

C 0.70041500 3.35903300 0.05431200

H 0.09503300 3.73656600 -1.98806100

H 1.14160900 2.70197500 2.06953300

H 1.28460700 4.27897400 0.13877200

C -3.55068800 0.77870300 1.03177800

H -2.89096900 1.56290700 1.43482200

H -4.25398200 0.50506700 1.83859100

C -4.32310200 1.30693500 -0.14808500

H -5.05511800 0.61309900 -0.58141100

C -4.15632200 2.51166100 -0.70017300

H -3.41986200 3.22072400 -0.30826500

H -4.74243400 2.83083300 -1.56675600

N -0.77624500 -1.51852100 -0.00801100

S 0.47779700 -2.02865900 -0.97678400

O 0.73454300 -3.42254300 -0.60735100

O 0.13405600 -1.62283400 -2.34165400

C 1.89886300 -1.06425300 -0.46038800

C 2.24298200 0.08818300 -1.17138700

C 2.59111400 -1.43205900 0.69590300

C 3.27436300 0.89415100 -0.69575000

H 1.69213500 0.34615300 -2.07570800

C 3.62019600 -0.60971700 1.16010100

H 2.33184700 -2.35764700 1.21326300

C 3.97045600 0.56814100 0.48097800

H 3.53756900 1.80324000 -1.24299300

H 4.16464000 -0.89145800 2.06557500

C 5.05478900 1.48003300 0.99749400

H 5.78124900 1.72586700 0.20610800

H 5.60386200 1.03040300 1.83781000

H 4.62561900 2.43461600 1.34765700

C -3.42474600 -1.80667200 0.65659600

H -4.46212900 -1.83698700 1.00127400

H -3.18761600 -2.50682300 -0.15045000

**3**

S -0.55480300 -1.62119800 0.88869100

O -0.52034100 -2.96401000 0.30033600

O -0.35706300 -1.39645300 2.32409200

N 0.56024400 -0.67086400 0.07561200

C 1.42473700 0.26108300 0.81551500

H 1.02891100 0.27986400 1.84018400

C 2.86922000 -0.22927700 0.91122500

C 3.62097100 -0.44306900 -0.38747700

H 4.70626200 -0.37642600 -0.20938400

H 3.37411200 0.36728900 -1.09234800

C 3.29416600 -1.79698100 -1.01201300

H 3.79941300 -2.63497600 -0.52018200

C 3.01224900 -1.94321100 -2.48734600

H 3.36312900 -2.83954000 -3.00642700

H 3.03974500 -1.03404000 -3.09846900

C 1.89211200 -2.08404200 -1.48479300

H 1.50667900 -3.08661600 -1.28159500

C 0.84580300 -0.99834300 -1.32534300

H 1.16502800 -0.06371300 -1.81083400

H -0.09323800 -1.29091800 -1.82051800

C -2.85561000 -1.42209100 -0.63552700

H -2.48642400 -2.31133700 -1.14925600

C -4.04960400 -0.80524600 -1.01845800

H -4.61633100 -1.21303600 -1.85997100

C -4.53676300 0.32144900 -0.33827000

C -3.79506400 0.81746600 0.74978300

H -4.16281500 1.68934100 1.29808900

C -2.60190800 0.21636800 1.14509100

H -2.03347900 0.59763400 1.99517700

C -2.13442900 -0.89738900 0.43865300

C -5.83939300 0.97066000 -0.73337500

H -6.64698300 0.67807000 -0.04010400

H -6.15065600 0.67742400 -1.74690500

H -5.76729000 2.06920000 -0.69918700

C 0.13312500 2.10532500 -0.39181800

H -0.67473800 1.39006100 -0.55431700

C -0.00786700 3.42392500 -0.83304100

H -0.92722600 3.72728100 -1.34099100

C 1.01889500 4.34990600 -0.62680600

H 0.91081800 5.38066600 -0.97389600

C 2.18583900 3.94460900 0.02744700

H 2.99586700 4.65908300 0.19628200

C 2.32495100 2.62534200 0.46849200

H 3.23815700 2.31942800 0.98372200

C 1.30122600 1.68763900 0.26418900

C 3.41561100 -0.48228600 2.10669900

H 4.45094800 -0.82584700 2.19709600

H 2.84487500 -0.37098900 3.03310300

**4**

C -4.13736200 3.37491600 1.72302400

C -4.94611100 3.10815800 0.60684800

C -4.49692900 2.29001800 -0.43129900

C -3.21979000 1.73020600 -0.34568900

C -2.38645000 1.98346800 0.74906100

C -2.85527800 2.79845400 1.77475300

H -5.94446100 3.54896700 0.54550000

H -5.12017700 2.08566600 -1.30273000

H -1.38138800 1.56947500 0.78400000

H -2.20407600 3.00267600 2.62875800

C -4.60903600 4.28013500 2.83180100

H -4.10460100 5.25974100 2.77036400

H -4.37272100 3.85758200 3.82119900

H -5.69307800 4.45887700 2.78191900

S -2.66489900 0.69627300 -1.68892700

O -1.34438500 1.15278700 -2.22272900

O -3.74589300 0.61626900 -2.67932700

N -2.30842500 -0.76017700 -0.96425000

C -1.43520700 -1.60881900 -1.80877300

H -1.70947900 -2.65002000 -1.58934900

H -1.68830300 -1.44111600 -2.86753700

C 0.05378800 -1.40866600 -1.58764300

H 0.47366400 -2.08128600 -0.83725100

C 0.88638800 -0.91753300 -2.61772900

H 0.43315700 -0.58983500 -3.56214100

H 1.92714100 -1.24231800 -2.69978800

C -3.16246900 -1.36490400 0.06973500

H -3.53185000 -0.53784200 0.69906700

C -2.27237200 -2.22343000 0.96965300

C -1.17804500 -1.62245800 1.61279300

C -2.50799700 -3.58848700 1.16649000

C -0.34607800 -2.36566800 2.44992100

H -0.96036600 -0.56836300 1.43649300

C -1.66555400 -4.34126800 1.99461800

H -3.35385200 -4.06622400 0.66839700

C -0.58670700 -3.73259400 2.64029800

H 0.49147100 -1.87494800 2.94916600

H -1.85814900 -5.40806100 2.13538400

H 0.06688300 -4.31865300 3.29165800

C -4.37421000 -2.06351300 -0.48428000

C -4.71514500 -2.53438300 -1.86864100

C -5.72798300 -2.22079800 0.14797100

C -5.57478100 -1.52574100 -1.16842900

H -5.08445000 -3.55996300 -2.02679600

H -4.09869500 -2.13581500 -2.68037800

H -6.19926000 -3.21519600 0.19815400

H -5.92767000 -1.58679900 1.02115900

H -5.80163200 -0.50411000 -1.46700800

C 3.24667800 -1.45961300 -0.39224500

C 4.33923500 -1.56289600 -1.26852900

H 4.79276500 -0.66093300 -1.68433900

C 4.85589400 -2.81582800 -1.61381300

H 5.70779500 -2.87855600 -2.29569200

C 4.28976100 -3.98101500 -1.08938200

H 4.69548000 -4.95914100 -1.36003500

C 3.20208300 -3.88819900 -0.21494200

H 2.75114000 -4.79204900 0.20258900

C 2.68223300 -2.63866500 0.12878100

H 1.83821700 -2.58456400 0.81942600

C 3.85080200 1.35823700 -0.33398800

C 3.85928400 2.11051800 -1.51873000

H 3.01330200 2.04066000 -2.20526700

C 4.93270800 2.95828700 -1.80548000

H 4.92734000 3.54271600 -2.72890000

C 5.99940000 3.06989200 -0.90888300

H 6.83460200 3.73946300 -1.13061900

C 5.99143300 2.32908000 0.27769800

H 6.81891800 2.41816200 0.98636000

C 4.92321700 1.47554000 0.56418300

H 4.92709100 0.90615200 1.49569500

C 2.27063900 0.14862500 1.80725200

C 1.31991600 0.98754500 2.41329700

C 3.06028300 -0.68007100 2.62534400

C 1.14463300 0.97409200 3.79942900

H 0.73886500 1.67063400 1.79168100

C 2.88339800 -0.68887300 4.01232400

H 3.81347100 -1.33442200 2.18395500

C 1.91812700 0.13147300 4.60350300

H 0.39645700 1.62932700 4.25312100

H 3.50200800 -1.34400900 4.63111500

H 1.77329300 0.11713500 5.68676100

Cl 0.67557100 2.86338100 -0.40224900

Rh 0.60301300 0.61990700 -1.19955000

P 2.46240500 0.17938500 -0.03992700

**TS5**

C -7.14893500 1.36540400 1.27101000

C -6.18269900 2.37809600 1.16666200

C -5.11109600 2.26858100 0.27750000

C -4.99805300 1.12158000 -0.51105600

C -5.95640800 0.10407000 -0.44406700

C -7.01996300 0.23225800 0.44739100

H -6.27226700 3.27365500 1.78738000

H -4.37599900 3.06893400 0.17793600

H -5.86815400 -0.76748400 -1.09481900

H -7.77080300 -0.56095800 0.50276000

C -8.31992000 1.49554500 2.21213100

H -8.14866300 2.27251500 2.97161700

H -9.23609400 1.76645200 1.65939900

H -8.52491800 0.54580700 2.73088700

S -3.58746100 0.93076400 -1.60004700

O -3.07710600 2.27430300 -1.88148100

O -3.99489400 0.02299800 -2.68501700

N -2.44651000 0.11058000 -0.68919200

C -1.67603500 0.84673600 0.30780300

H -2.20614100 1.79054400 0.51724900

H -1.68731200 0.26773500 1.24579400

C -0.25136400 1.16188500 -0.08495500

H 0.26447400 1.65683900 0.74459600

C 0.17002200 1.44854200 -1.40185100

H -0.56108600 1.40674800 -2.21367100

H 0.98092700 2.16436000 -1.56715000

C -2.13337400 -1.29874100 -0.96804300

H -2.97920100 -1.65548700 -1.57005400

C -2.14734500 -2.11505200 0.32448400

C -3.31343100 -2.06333900 1.10848100

C -1.07716800 -2.90153200 0.76684400

C -3.40751700 -2.77302900 2.30559600

H -4.15063400 -1.44119700 0.78412200

C -1.17215000 -3.61622800 1.96856200

H -0.14886500 -2.95182800 0.20011900

C -2.33140700 -3.55498200 2.74321100

H -4.32256900 -2.71315500 2.90084200

H -0.31982300 -4.21794300 2.29545100

H -2.39974200 -4.10996900 3.68256900

C -0.82348900 -1.29905900 -1.79277400

C -0.96685600 -0.96241500 -3.27549100

C -0.21632900 -3.14638500 -2.54096500

C -1.20116700 -2.41096800 -3.15936200

H -0.03525100 -0.62619100 -3.74192800

H -1.85006400 -0.39177100 -3.57766500

H 0.84390200 -2.90149000 -2.59554400

H -0.46386100 -4.09823400 -2.06231500

H -2.23002000 -2.77939900 -3.17288600

Rh 0.91951700 -0.45621000 -0.80572500

Cl 2.19170200 -2.52614100 -0.64837300

P 2.79341000 0.41130200 0.27416100

C 2.94224300 2.26017900 0.35577200

C 2.92633800 -0.07732300 2.05010600

C 4.38662700 -0.06861400 -0.52818300

C 2.34661700 2.98924200 1.40118100

C 3.54286800 2.97165100 -0.69762300

C 2.09021600 -1.09606300 2.53620400

C 3.81515500 0.55612500 2.93877100

C 4.39298600 -0.16528400 -1.93027700

C 5.57112600 -0.32126300 0.17696700

H 1.88406800 2.46454400 2.23982300

C 2.35442100 4.38682000 1.39417500

C 3.55029300 4.36897900 -0.70318100

H 4.01636200 2.43364600 -1.52095600

H 1.39631200 -1.59277400 1.85760400

C 2.14851300 -1.47757500 3.87982000

C 3.87546800 0.16824400 4.27941700

H 4.45248400 1.37069000 2.58732200

C 5.56912300 -0.47675100 -2.61465100

H 3.46141600 -0.01457900 -2.48161200

C 6.74537200 -0.64655000 -0.50965500

H 5.58452900 -0.28519000 1.26681500

H 1.88905800 4.93311600 2.21876400

C 2.95516700 5.08229100 0.34146000

H 4.02692100 4.90203300 -1.52989500

H 1.48653200 -2.26863500 4.24138000

C 3.04174400 -0.85098100 4.75285700

H 4.57193000 0.66814600 4.95767900

C 6.75014400 -0.71811700 -1.90511200

H 5.55918800 -0.54738900 -3.70538000

H 7.66033800 -0.84975400 0.05298500

H 2.96166400 6.17519400 0.33602800

H 3.08610500 -1.15081700 5.80326000

H 7.66945000 -0.97295100 -2.43884100

**6**

C -7.57526800 0.54925300 0.24293200

C -6.86654300 1.11064900 1.32326400

C -5.56720500 1.58412500 1.16592500

C -4.96039100 1.49361500 -0.09221600

C -5.64080200 0.95906700 -1.18565500

C -6.94490200 0.48682400 -1.00790200

H -7.34758700 1.17827000 2.30311800

H -5.02145200 2.02518200 2.00218700

H -5.15203900 0.92355500 -2.16064400

H -7.48096800 0.06356500 -1.86136200

C -8.98124800 0.04265800 0.44201600

H -9.01886300 -0.71395500 1.24294300

H -9.65572000 0.86214600 0.74185100

H -9.38640900 -0.41095800 -0.47400000

S -3.26111900 2.03043500 -0.26896800

O -3.04076600 3.19432800 0.59146200

O -2.97641400 2.08682300 -1.71218200

N -2.41673400 0.74792500 0.42397300

C -1.40539200 1.00138100 1.44324300

H -1.74852000 1.85267800 2.05249600

H -1.37990100 0.11727300 2.09970100

C -0.01677400 1.29394200 0.91740900

H 0.72355800 1.32467700 1.72433500

C 0.24654600 2.03168800 -0.25386900

H -0.56808300 2.39630900 -0.88450900

H 1.17324900 2.60811600 -0.32916400

C -2.30502200 -0.47371900 -0.39245400

H -3.19637800 -0.48351100 -1.04126800

C -2.42966600 -1.71632000 0.49111100

C -3.52127600 -1.77780800 1.37335000

C -1.53766900 -2.79323200 0.43103300

C -3.71199300 -2.89298600 2.18981200

H -4.22024700 -0.93930000 1.41989300

C -1.73335600 -3.91185700 1.25056900

H -0.68409800 -2.77013300 -0.24710600

C -2.81448000 -3.96616700 2.13222800

H -4.56525200 -2.92598400 2.87255100

H -1.02723500 -4.74435700 1.19387700

H -2.96094500 -4.84009900 2.77241500

C -1.05938500 -0.39425900 -1.26495200

C -1.29340500 -0.59357500 -2.73314200

C -1.81939200 -3.01280300 -3.14904700

C -2.22562100 -1.74209900 -3.07317000

H -0.33620100 -0.69583600 -3.26116300

H -1.74735800 0.36984200 -3.04197300

H -0.77218100 -3.27758500 -2.96935700

H -2.52001500 -3.81918900 -3.38444300

H -3.28196200 -1.50014200 -3.24023100

Rh 0.66777200 -0.04056700 -0.60910500

Cl 1.44778400 -2.13868500 -1.57294100

P 3.07273100 0.17737900 0.15029200

C 3.52465800 1.85684800 0.77321800

C 3.55973900 -0.94844000 1.52688600

C 4.30726400 -0.10355900 -1.18723700

C 3.19541200 2.24478200 2.08532800

C 4.06133400 2.82034100 -0.09863200

C 2.84168100 -2.14528100 1.69522800

C 4.62176400 -0.65965100 2.40400600

C 3.89214600 0.07802100 -2.51623800

C 5.64436100 -0.44021600 -0.92564300

H 2.79103600 1.51167100 2.78723900

C 3.40140000 3.55845200 2.51354500

C 4.26390600 4.13479200 0.33174100

H 4.32641400 2.54336700 -1.12120800

H 2.02797600 -2.38867300 1.00996300

C 3.18264300 -3.03246400 2.72051800

C 4.95813200 -1.54979200 3.42687000

H 5.18354300 0.27090600 2.29798100

C 4.80602900 -0.05513400 -3.56511500

H 2.84482000 0.30207300 -2.72859900

C 6.55370800 -0.58275300 -1.97670000

H 5.98070000 -0.60167000 0.10019100

H 3.14378500 3.83931100 3.53798600

C 3.93404800 4.50914100 1.63747500

H 4.68462900 4.86941800 -0.35965000

H 2.61538900 -3.95902100 2.83984400

C 4.23824900 -2.73832700 3.58791400

H 5.78498200 -1.31228000 4.10133000

C 6.13756600 -0.38606000 -3.29764700

H 4.47112800 0.08647800 -4.59572400

H 7.59148000 -0.85105600 -1.76244200

H 4.09296900 5.53725400 1.97215300

H 4.50027500 -3.43285800 4.39042200

H 6.85013500 -0.49913500 -4.11889600

**7**

C -6.53876200 0.25479100 -1.01249800

C -6.37442700 1.24593200 -0.02992400

C -5.13747000 1.85392300 0.18186900

C -4.05273800 1.47100400 -0.60976300

C -4.19343500 0.51905500 -1.62177200

C -5.43531600 -0.08385600 -1.81275300

H -7.22825200 1.53890200 0.58689400

H -5.00425000 2.61526300 0.95274600

H -3.33297600 0.24698800 -2.23440800

H -5.54677700 -0.84482100 -2.58950400

C -7.85290300 -0.46254400 -1.18378800

H -8.08440100 -0.63527700 -2.24635700

H -7.81076300 -1.45175500 -0.69514100

H -8.68728200 0.09602500 -0.73452600

S -2.42914500 2.09211300 -0.19847700

O -2.55905900 3.40052300 0.44353800

O -1.58747600 1.91715300 -1.39718500

N -1.91755400 1.01146400 0.98882800

C -1.48212500 1.49548800 2.30404400

H -2.04404700 2.41969600 2.50885700

H -1.79054100 0.75181500 3.05713800

C 0.00627000 1.77466200 2.40319200

H 0.38544900 1.97742900 3.40952700

C 0.71077700 2.22264800 1.28550100

H 0.19031400 2.37242200 0.34151500

H 1.65145900 2.76132900 1.40193600

C -1.57457500 -0.33830300 0.54005400

H -1.28596600 -0.27398100 -0.52977500

C -2.73792500 -1.32515300 0.62123400

C -3.79565800 -1.14801900 1.52000800

C -2.76252700 -2.42449400 -0.25033700

C -4.86017500 -2.05337800 1.54932000

H -3.80427800 -0.27551900 2.17509400

C -3.82264500 -3.33309000 -0.21878500

H -1.95584400 -2.55593500 -0.97616100

C -4.87738500 -3.14977800 0.68172200

H -5.68515600 -1.89501800 2.24856000

H -3.83111900 -4.18052600 -0.90924900

H -5.71186100 -3.85535300 0.70328400

C -0.28159000 -0.81042200 1.12856600

C 0.02146800 -2.24454800 1.40187800

C 1.39524800 -1.45185400 3.31920000

C 1.37454600 -2.09147600 2.09343500

H 0.08769900 -2.81352300 0.45854100

H -0.75576400 -2.74590400 2.01183500

H 0.47587500 -1.28219600 3.88649300

H 2.33288100 -1.29512500 3.85487100

H 2.26549900 -2.57591500 1.68726100

Cl 3.34141900 1.07025600 2.62605600

C 1.42705500 -1.02735800 -1.69290800

C 2.93152100 1.52805700 -1.26379100

C 4.04585500 -1.03940300 -0.52855800

C 0.41545900 -0.43329900 -2.46936000

C 1.52925400 -2.43377100 -1.70228500

C 4.28065300 1.92464900 -1.29263900

C 1.95122400 2.44174500 -1.69663500

C 4.68705900 -1.39399700 0.66697000

C 4.61618700 -1.41903100 -1.75841300

H 0.27255200 0.64347400 -2.47861600

C -0.45051000 -1.21770200 -3.23879200

C 0.66004700 -3.21427300 -2.46933600

H 2.30107800 -2.92847100 -1.10964100

H 5.05785400 1.25408700 -0.92519700

C 4.64096000 3.18427500 -1.77819900

C 2.31857400 3.69420600 -2.19272800

H 0.88930500 2.20863300 -1.62936600

C 5.87666300 -2.13175800 0.63031800

H 4.27753600 -1.05059600 1.61904500

C 5.80126900 -2.15453200 -1.78909500

H 4.12772100 -1.13995400 -2.69525900

H -1.22204400 -0.72473400 -3.83605400

C -0.33482200 -2.61017800 -3.24494300

H 0.76668400 -4.30219100 -2.46212400

H 5.69496800 3.47331500 -1.78975800

C 3.66409100 4.07007400 -2.23918900

H 1.54021900 4.38404400 -2.52831900

C 6.43237700 -2.51592200 -0.59196200

H 6.37231600 -2.39840600 1.56718100

H 6.23556200 -2.44547600 -2.74911300

H -1.01265300 -3.21992200 -3.84742200

H 3.94813500 5.05463700 -2.61938500

H 7.36126800 -3.09192100 -0.61616000

Rh 1.26191500 0.11163100 1.61007700

P 2.47342700 -0.07753200 -0.48772200

**TS8**

C 6.83245400 0.53390000 0.15819900

C 6.30753700 1.33579800 -0.86817100

C 4.99020500 1.79626700 -0.82260100

C 4.18721500 1.44215100 0.26417400

C 4.68882600 0.66662400 1.31272900

C 6.00619600 0.21945800 1.25089500

H 6.94120200 1.60835500 -1.71641100

H 4.58956400 2.43242600 -1.61406900

H 4.04152600 0.40986400 2.15095900

H 6.39851100 -0.39834500 2.06289500

C 8.24206400 0.00246800 0.09645000

H 8.79022100 0.21534300 1.02841800

H 8.23824100 -1.09365200 -0.03111500

H 8.80852000 0.43642300 -0.74038500

S 2.45596000 1.89266800 0.25501400

O 2.32853600 3.19075500 -0.41436400

O 1.92941900 1.66895500 1.60794900

N 1.68659500 0.77702300 -0.72651500

C 1.72989100 0.87540700 -2.17891200

H 1.98682600 1.91308200 -2.43982400

H 2.50321400 0.22026100 -2.61666300

C 0.35930900 0.54368500 -2.72658100

H 0.34154900 0.10075000 -3.72781900

C -0.73586500 1.36463700 -2.30936300

H -0.53297000 2.11109700 -1.53608100

H -1.48636000 1.65205100 -3.05016000

C 1.26766900 -0.52353700 -0.19482000

H 0.74594900 -0.32980700 0.75414300

C 2.42959900 -1.45737700 0.13387900

C 3.44803400 -1.76482500 -0.78286900

C 2.50276200 -2.00621600 1.42176600

C 4.51279700 -2.59008700 -0.41779600

H 3.42412500 -1.34891000 -1.79036700

C 3.56295200 -2.84113400 1.78830600

H 1.72515700 -1.76362100 2.14864700

C 4.57427400 -3.13375100 0.87004900

H 5.30118100 -2.80769800 -1.14273700

H 3.60093800 -3.25558400 2.79913200

H 5.40817900 -3.78039100 1.15472800

C 0.18920100 -1.07422600 -1.13225000

C 0.34077500 -2.50449500 -1.60543500

C -1.34907000 -2.80862200 -3.45034800

C -0.97471500 -2.98857400 -2.17564200

H 0.64742200 -3.12896700 -0.74754300

H 1.16258100 -2.59395300 -2.33999800

H -0.67459600 -2.35009100 -4.18069900

H -2.34695700 -3.07885400 -3.80018300

H -1.67534000 -3.45216700 -1.47053400

Cl -3.52683500 0.01754000 -3.00655700

C -1.60311500 -0.66087200 1.81003600

C -2.45890700 1.92918800 0.66847600

C -4.23194000 -0.37292300 0.80844300

C -1.04540600 0.03912100 2.88838600

C -1.50723600 -2.06565700 1.78978400

C -3.61935900 2.71275700 0.57143000

C -1.21456700 2.56416100 0.82599100

C -5.07277800 -0.95535100 -0.14988100

C -4.70113800 -0.18212700 2.12185900

H -1.10188200 1.12667500 2.93227500

C -0.40197300 -0.65259100 3.92374700

C -0.87997200 -2.75331000 2.82710100

H -1.93512500 -2.62266000 0.95200100

H -4.59195900 2.23842700 0.42725600

C -3.52996200 4.10669100 0.64593600

C -1.12818100 3.95431600 0.90110500

H -0.29921700 1.97917900 0.88742000

C -6.37016000 -1.34374400 0.20743000

H -4.72335200 -1.06547100 -1.17936200

C -5.99540600 -0.56849400 2.46985200

H -4.05015300 0.26650400 2.87649400

H 0.03830600 -0.08966700 4.75032200

C -0.32197900 -2.04556100 3.90056400

H -0.82014200 -3.84416900 2.79789900

H -4.43889200 4.70852200 0.56622900

C -2.28920500 4.73019000 0.81144100

H -0.14418800 4.41668800 1.00917100

C -6.83254600 -1.15383400 1.51125700

H -7.02231000 -1.79207300 -0.54653500

H -6.35180200 -0.41632400 3.49202500

H 0.17750400 -2.58195700 4.71132700

H -2.22636800 5.82026500 0.86207600

H -7.84628800 -1.45878500 1.78468800

Rh -1.54974600 -0.42826000 -1.59252300

P -2.50152600 0.11694400 0.38442600

**9**

C 6.70010600 0.68848400 -0.16528500

C 6.08764900 1.00692800 -1.38779900

C 4.74971600 1.40256900 -1.44322500

C 4.01404600 1.47085100 -0.25715800

C 4.60504600 1.18896700 0.97690600

C 5.94181500 0.80099300 1.01269100

H 6.66789100 0.94795000 -2.31264000

H 4.28055900 1.66991600 -2.39160300

H 4.01010300 1.26180700 1.88699400

H 6.40414200 0.56612900 1.97497200

C 8.12961900 0.21341500 -0.10422700

H 8.69523000 0.74212300 0.67963000

H 8.16846000 -0.86225600 0.14060500

H 8.65210300 0.35804400 -1.06124700

S 2.26389100 1.83653100 -0.33545300

O 2.07058000 2.79492300 -1.43055000

O 1.80131000 2.12602800 1.02787600

N 1.49073200 0.43790500 -0.80488600

C 1.50860200 0.00346000 -2.19950700

H 1.21876300 0.83670400 -2.85376300

H 2.51256100 -0.34282700 -2.51077800

C 0.49462000 -1.14906200 -2.20285400

H 0.83498100 -1.95731000 -2.87058200

C -0.93538800 -0.73750400 -2.48689300

H -1.09935100 0.34501100 -2.48145500

H -1.43412300 -1.20398700 -3.34712300

C 1.24334700 -0.67969500 0.12938400

H 0.70347300 -0.26983500 0.98536700

C 2.51255400 -1.32800600 0.67246000

C 3.49780200 -1.87579400 -0.16613200

C 2.71305400 -1.38002000 2.05867000

C 4.65507400 -2.44399200 0.36714100

H 3.36560500 -1.85514100 -1.24867900

C 3.86588200 -1.96074900 2.59820600

H 1.96154200 -0.94077100 2.71875400

C 4.84317200 -2.49149800 1.75344500

H 5.41595700 -2.85261500 -0.30273600

H 4.00378400 -1.98814800 3.68240200

H 5.74889500 -2.93937100 2.17029200

C 0.36155800 -1.63051200 -0.71672200

C 0.54117900 -3.13645700 -0.49687900

C -1.46085100 -3.72094700 -1.97359800

C -0.85384100 -3.67642800 -0.74817100

H 0.83856400 -3.34808700 0.53983500

H 1.30647200 -3.59636400 -1.14967400

H -0.91011900 -3.50644000 -2.89291200

H -2.47264500 -4.11170300 -2.08943400

H -1.40831300 -4.06694300 0.11688600

Cl -4.10374000 -1.85835400 -1.53175700

C -1.42500400 0.73386000 1.79503100

C -2.27089400 1.81587700 -0.87209600

C -4.04986900 0.39740500 0.96378400

C -1.19688000 2.02853500 2.28096900

C -1.05780700 -0.36666100 2.58858100

C -3.28159800 1.93335400 -1.84605600

C -1.14111900 2.64292700 -0.94292800

C -4.56779300 -0.77496700 1.53523600

C -4.77294100 1.59366500 1.07805400

H -1.49745600 2.89927000 1.69747400

C -0.57459000 2.21743800 3.51723300

C -0.45551800 -0.17646800 3.83318200

H -1.23454800 -1.38213100 2.22231300

H -4.13242600 1.24721100 -1.83320000

C -3.17003200 2.89640900 -2.84974900

C -1.02671400 3.59330800 -1.96192500

H -0.33252500 2.53986900 -0.22375900

C -5.79122600 -0.75143400 2.20547300

H -4.02264200 -1.71544000 1.44314800

C -6.00436100 1.61201400 1.74077200

H -4.38154700 2.51761100 0.64785600

H -0.37908200 3.23232000 3.87109400

C -0.20154200 1.11920000 4.29535300

H -0.17641900 -1.04256700 4.43864200

H -3.95877100 2.98504300 -3.60109600

C -2.04391800 3.72782300 -2.90981700

H -0.11937000 4.19797600 -2.01300800

C -6.51569400 0.44113200 2.30571700

H -6.18724000 -1.67279500 2.63962200

H -6.56339700 2.54833900 1.81526900

H 0.28398900 1.27150100 5.26255000

H -1.95468800 4.46972300 -3.70771200

H -7.47868600 0.45614600 2.82261600

Rh -1.77681600 -1.61570200 -0.84606100

P -2.34969900 0.37018000 0.24046300

**TS10**

C -6.51990500 -0.87417300 0.56287600

C -5.57275300 -0.69021800 1.58506700

C -4.23561600 -1.03162600 1.40057600

C -3.83713700 -1.56733000 0.17308900

C -4.75917300 -1.79442600 -0.85134900

C -6.09481900 -1.44096500 -0.64929400

H -5.88715400 -0.25777400 2.53851800

H -3.49461700 -0.87469600 2.18458000

H -4.43271900 -2.25365600 -1.78620000

H -6.82176900 -1.61296100 -1.44769500

C -7.95074000 -0.44718100 0.77026400

H -8.02492700 0.65382700 0.79669500

H -8.34498200 -0.81867400 1.72967700

H -8.60842400 -0.80842200 -0.03386800

S -2.10228600 -1.89439500 -0.12157000

O -1.42754700 -1.86762000 1.18052600

O -2.02567900 -3.05392300 -1.01681300

N -1.50605400 -0.60503700 -0.99558200

C -1.61692600 -0.54696900 -2.45428400

H -2.66627900 -0.45679600 -2.79478600

H -1.19032000 -1.45933300 -2.89559000

C -0.83045200 0.71102900 -2.79022500

H -1.19988200 1.27076400 -3.65692700

C 0.66303100 0.64547200 -2.60857200

H 1.01415600 -0.35862400 -2.38608500

H 1.24397400 1.16853100 -3.37257000

C -1.22490900 0.69958600 -0.37007900

C -0.56659300 1.49406200 -1.53308600

C -0.69471200 3.02336300 -1.61562300

H -0.98063000 3.31902700 -2.63911400

H -1.49452600 3.36758100 -0.94779100

Rh 1.63132800 1.74327500 -0.84744700

C 0.63907000 3.61143700 -1.22258700

C 1.72354100 3.55384600 -2.09102300

H 2.64773200 4.08323000 -1.84933100

H 1.59079400 3.27695300 -3.14211100

H 0.70923400 4.20557300 -0.30724000

C -2.45301000 1.41483300 0.17274900

C -3.60869800 1.59320800 -0.60463300

C -2.43293300 1.91983200 1.47838500

C -4.72151600 2.25141900 -0.08231300

H -3.64641100 1.20240300 -1.62259800

C -3.54515000 2.58731500 2.00283100

H -1.54141000 1.77471400 2.09201600

C -4.69325500 2.75267000 1.22474500

H -5.61869300 2.36906800 -0.69500500

H -3.51447200 2.97258100 3.02519700

H -5.56588600 3.26795000 1.63400200

H -0.54206800 0.53142700 0.46659200

Cl 3.04422900 2.87701200 0.67811000

P 2.45468500 -0.34385100 0.14684900

C 4.23545300 -0.49910900 0.66269700

C 1.64242000 -0.71769600 1.75867300

C 2.18479100 -1.79634300 -0.95560100

C 5.14874000 -1.38444600 0.06745700

C 4.66612400 0.27669800 1.75602200

C 1.72826900 -1.98581500 2.35637900

C 0.98639700 0.31799100 2.44170000

C 2.89141700 -1.81420900 -2.17710500

C 1.19102000 -2.76092800 -0.74100000

H 4.84555800 -2.03272200 -0.75341300

C 6.46448700 -1.47332900 0.53540400

C 5.97872200 0.18354400 2.21962800

H 3.97359800 0.96081900 2.24338500

H 2.28154400 -2.78774000 1.86314300

C 1.10614200 -2.23074700 3.58031200

C 0.37449700 0.07203400 3.67452100

H 0.98444500 1.32544300 2.01845700

C 2.64391600 -2.80191900 -3.13026100

H 3.61730400 -1.02707100 -2.39762100

C 0.92692200 -3.73660900 -1.70953900

H 0.58863400 -2.74074300 0.16523300

H 7.15711200 -2.17125800 0.05758400

C 6.88671700 -0.68667500 1.60826600

H 6.29176900 0.79844400 3.06727700

H 1.16124300 -3.22705400 4.02610200

C 0.41773200 -1.20518000 4.23712100

H -0.13308800 0.88641600 4.19841800

C 1.65946100 -3.77012400 -2.89696200

H 3.20675500 -2.80288000 -4.06729500

H 0.11789600 -4.44659600 -1.53189000

H 7.91488300 -0.75704100 1.97283700

H -0.07136800 -1.40058500 5.19493500

H 1.45240300 -4.53498700 -3.64985000

**11**

C 7.07102600 -1.12080900 0.63310600

C 7.15945600 0.20237800 0.17224100

C 6.09836100 1.09623000 0.33463800

C 4.92829800 0.65564100 0.95830600

C 4.82134800 -0.64866700 1.44765100

C 5.89091200 -1.52525000 1.28078400

H 8.07681600 0.54236000 -0.31624700

H 6.17725300 2.13151300 -0.00214200

H 3.90202800 -0.96417900 1.94024000

H 5.80453700 -2.54951800 1.65362100

C 8.20400200 -2.09785100 0.44103400

H 9.08958400 -1.61753000 -0.00035000

H 8.50471300 -2.55250700 1.39918700

H 7.90059200 -2.92365700 -0.22485400

S 3.51514300 1.75916000 1.05843300

O 4.04220100 3.11970500 1.17872700

O 2.56733100 1.18326800 2.01431100

N 2.78855100 1.66929100 -0.43766300

C 3.33073300 2.35565500 -1.60704400

H 3.58376600 3.39275900 -1.34140800

H 4.24322500 1.86368400 -1.99929200

C 1.75144000 0.67177800 -0.74838100

H 0.98080300 0.75009100 0.03697200

C 2.27026000 -0.75461400 -0.74319600

C 1.77176000 -1.65899100 0.20453900

C 3.27978600 -1.17833100 -1.61995900

C 2.26128100 -2.96661700 0.26827400

H 0.99394700 -1.33099200 0.89810600

C 3.77699800 -2.48163700 -1.55352400

H 3.68642900 -0.48007700 -2.35520100

C 3.26670600 -3.38117000 -0.61029100

H 1.85402500 -3.65738200 1.01096800

H 4.56869000 -2.79682700 -2.23799500

H 3.65544300 -4.40160100 -0.56007400

C 2.19270500 2.24406100 -2.60228000

H 2.43027600 2.31270400 -3.66766800

C 0.84109700 2.66934600 -2.10947100

H 0.76557300 3.18305600 -1.14833400

H 0.10148100 2.97913800 -2.85189400

C 1.21773900 1.18890700 -2.11010200

C 0.52060600 0.26357700 -3.10611300

H 0.44811600 0.81195700 -4.05741700

H 1.15989100 -0.61476800 -3.29375000

Rh -1.27917800 0.72654700 -0.82991900

P -3.04558200 -0.16210200 0.15234000

C -4.32129800 -1.03666700 -0.85077600

C -4.45295800 -0.73172600 -2.21329900

H -3.75828200 -0.02511400 -2.67196300

C -5.47137000 -1.31543400 -2.97298700

H -5.56494300 -1.07014500 -4.03385800

C -6.36710300 -2.20691300 -2.37656500

H -7.16179000 -2.66550100 -2.97049100

C -6.25025900 -2.50474400 -1.01397300

H -6.95530900 -3.19276100 -0.54060000

C -5.23820900 -1.91776400 -0.25193400

H -5.16661000 -2.14157800 0.81483400

C -4.12648200 0.91213500 1.18183700

C -4.50924400 2.16068600 0.66782100

H -4.07870400 2.51143500 -0.27217600

C -5.41127800 2.95964200 1.37092100

H -5.69951000 3.93366400 0.96794100

C -5.92952500 2.52516500 2.59620600

H -6.62835000 3.15721700 3.15022500

C -5.54450200 1.28618900 3.11484300

H -5.93973600 0.94538800 4.07524300

C -4.64721300 0.47781400 2.40941200

H -4.34759000 -0.48611400 2.82630200

C -2.31327500 -1.39187400 1.31222500

C -1.53560500 -0.87919600 2.36982300

C -2.32347400 -2.77425800 1.07388900

C -0.79679400 -1.74159500 3.17953600

H -1.47672600 0.20115700 2.52653400

C -1.57635500 -3.63407400 1.88750000

H -2.90102200 -3.18848700 0.24551400

C -0.81405800 -3.12199800 2.94027300

H -0.18851600 -1.32948000 3.98817200

H -1.58850900 -4.70907600 1.69012200

H -0.22735000 -3.79497500 3.57090200

Cl -1.19681200 2.46082100 0.74135300

C -0.88151600 -0.20611200 -2.70574500

C -1.05357000 -1.22617500 -1.76104100

H -1.96632300 -1.82612200 -1.77889800

H -0.19328100 -1.73173200 -1.31636200

H -1.67304900 -0.01883600 -3.44030400

**TS12**

C -7.17672000 1.19320000 0.29212400

C -6.58014900 0.88912700 -0.94646200

C -5.65875700 -0.14667600 -1.06805700

C -5.31575400 -0.88992100 0.06735600

C -5.90256600 -0.62470900 1.30411000

C -6.83047200 0.41650300 1.40730500

H -6.84268700 1.47995000 -1.82834500

H -5.20188400 -0.37344600 -2.03331300

H -5.62168800 -1.22543400 2.17070900

H -7.29003400 0.62952400 2.37621300

C -8.16344300 2.32845200 0.39786300

H -7.70660500 3.27861600 0.07470400

H -9.03661100 2.15462800 -0.25280800

H -8.52864400 2.45913400 1.42685600

S -4.03366200 -2.14036800 -0.06160200

O -4.31915000 -2.98677600 -1.22104700

O -3.86046400 -2.71087000 1.27896700

N -2.64311200 -1.27485900 -0.42528700

C -2.22107900 -1.10450200 -1.80695600

H -2.88482700 -1.72055400 -2.43256100

H -2.36144200 -0.05660500 -2.12163500

C -0.80356900 -1.53113200 -2.09620400

H -0.44484200 -1.13556100 -3.05020100

C -0.15574700 -2.64859300 -1.57912300

H -0.64667600 -3.27588800 -0.83206900

H 0.63825200 -3.12691100 -2.15925800

C -2.10229100 -0.40272500 0.62743600

H -2.79143500 -0.51511700 1.48061200

C -2.13156000 1.07494600 0.26368400

C -3.21102500 1.85156100 0.71784000

C -1.17677000 1.67889600 -0.56560500

C -3.33570300 3.19313000 0.34940400

H -3.97336300 1.39897200 1.35595200

C -1.30238500 3.01900400 -0.94296300

H -0.33064500 1.08164500 -0.93246000

C -2.38185300 3.78127600 -0.48716500

H -4.18434900 3.77720000 0.71481000

H -0.54838300 3.46064800 -1.59774900

H -2.48041400 4.82888700 -0.78395600

C -0.83691700 -0.99474500 1.22959500

C 0.10925800 -0.46700700 2.26942100

C -0.74038800 -2.41794400 1.71120400

C 0.56323700 -1.69692100 1.49943100

H -0.15608300 -0.61682200 3.33004600

H 0.58926800 0.49290200 2.07039200

H -1.04988100 -2.60826200 2.75136900

H -1.04134000 -3.20859700 1.02271000

Rh 0.83188300 -0.92698400 -0.70242400

H 1.54752300 -2.10406500 1.25075700

Cl 1.92391700 -0.37177900 -2.75071700

P 2.85666700 0.18367800 0.10231900

C 3.22859000 0.39382100 1.91439000

C 4.38472800 -0.73662700 -0.37886800

C 3.02947200 1.87796500 -0.59208100

C 3.53805200 -0.76896500 2.64582200

C 3.16074200 1.61556500 2.60196500

C 4.30683500 -1.96818100 -1.04485600

C 5.64552700 -0.23871000 -0.00284200

C 2.06905000 2.83903000 -0.23442000

C 4.02129100 2.22166500 -1.52288300

H 3.63790000 -1.72770800 2.13110500

C 3.74653000 -0.71563500 4.02412300

C 3.37392500 1.66892900 3.98405000

H 2.95321700 2.54124600 2.06518200

H 3.33310600 -2.35660500 -1.34489100

C 5.47163300 -2.68620000 -1.33493900

C 6.80704900 -0.95121400 -0.30514200

H 5.71882800 0.70954600 0.53508200

C 2.12842500 4.13282000 -0.75743200

H 1.25333800 2.57692200 0.44212400

C 4.07148700 3.51350500 -2.05341200

H 4.74600200 1.47647400 -1.85157400

H 3.98476900 -1.63169700 4.57067600

C 3.65833500 0.50533300 4.70171100

H 3.32115300 2.63193600 4.49861200

H 5.39789500 -3.64523800 -1.85374600

C 6.72162800 -2.17901000 -0.97159400

H 7.78114400 -0.55011100 -0.01367400

C 3.13353300 4.47517100 -1.66696300

H 1.37638600 4.86848900 -0.46146000

H 4.84635100 3.76570300 -2.78178600

H 3.82133300 0.54905200 5.78147300

H 7.63049700 -2.74015000 -1.20412900

H 3.17775500 5.48481000 -2.08318200

**13**

C -7.24871800 0.66359600 0.19876900

C -6.66783200 0.30653800 -1.03313200

C -5.55108400 -0.52292000 -1.09090300

C -4.99816100 -1.00239500 0.10142900

C -5.56223600 -0.68323100 1.33667800

C -6.68257000 0.15189600 1.37621400

H -7.10065800 0.68930900 -1.96158000

H -5.10408200 -0.79799700 -2.04798500

H -5.11966200 -1.08596700 2.24919100

H -7.12348100 0.41034500 2.34269300

C -8.46828100 1.54949900 0.23788900

H -8.37827900 2.38748400 -0.47122400

H -9.37135100 0.98181200 -0.04573100

H -8.63908100 1.96472700 1.24199600

S -3.48720500 -1.96495000 0.02800800

O -3.55678400 -2.84736400 -1.13987900

O -3.25852000 -2.50009300 1.37797700

N -2.31215900 -0.82859200 -0.30685800

C -1.54151100 -0.89461100 -1.54291400

H -2.20776200 -1.22681100 -2.35621500

H -1.23399600 0.13085400 -1.79444900

C -0.33575700 -1.80442100 -1.52975500

H 0.41129400 -1.59174300 -2.29737000

C -0.22363000 -2.94674300 -0.81818300

H -1.01053700 -3.27506800 -0.13443300

H 0.61753300 -3.62304300 -0.96743000

C -1.97155300 0.15311400 0.74707100

H -2.77092600 0.06204700 1.50253300

C -2.06358900 1.56824500 0.18750100

C -3.32073100 2.18013300 0.05598200

C -0.93078800 2.26585400 -0.25024400

C -3.43800000 3.45596500 -0.50036500

H -4.21698000 1.65213900 0.38872400

C -1.04244600 3.54561400 -0.80232900

H 0.04861900 1.80467300 -0.14845200

C -2.29838300 4.14421900 -0.93211200

H -4.42501300 3.91685100 -0.59248600

H -0.13922700 4.07229100 -1.11938500

H -2.39012000 5.14517800 -1.36173600

C -0.67867600 -0.17876000 1.45442500

C 0.04693700 0.71417900 2.46719400

C -0.46536300 -1.37117600 2.39694100

C 0.80195400 -0.59747700 2.70633800

H -0.58277600 1.02909000 3.32868000

H 0.58076300 1.59597600 2.09524600

H -1.21700700 -1.58372700 3.17779100

H -0.30270700 -2.38139600 1.90393600

Rh 0.98985900 -1.23183200 0.72574800

H 1.55769200 -0.82254000 3.46899900

Cl 2.64458500 -3.05739300 0.66510200

P 2.55578500 0.16016200 -0.11402700

C 2.60182700 1.98768200 0.22065700

C 4.30347000 -0.16017100 0.39647600

C 2.52578700 -0.06149300 -1.93811800

C 2.74802100 2.35934300 1.57038400

C 2.61926500 2.99549800 -0.75594300

C 4.58070600 -0.71308500 1.65430100

C 5.36132700 0.29069000 -0.40898500

C 1.62918200 0.64971500 -2.75683800

C 3.24885100 -1.13436600 -2.48843900

H 2.80138200 1.58724700 2.34048600

C 2.85779800 3.70065900 1.93677000

C 2.73134600 4.34095500 -0.38725100

H 2.56947300 2.74544500 -1.81454100

H 3.76356600 -1.08396600 2.27196700

C 5.90180100 -0.81569300 2.09752100

C 6.68178200 0.17060000 0.03068700

H 5.15792400 0.74030600 -1.38373000

C 1.50034300 0.32900100 -4.11050700

H 0.99357500 1.43117800 -2.33833900

C 3.10845300 -1.45701900 -3.84057600

H 3.88969500 -1.74188800 -1.84634200

H 2.96634000 3.96554100 2.99135900

C 2.83743600 4.69967000 0.95790200

H 2.74414200 5.11061100 -1.16341000

H 6.10850700 -1.25373800 3.07713600

C 6.95417900 -0.38102100 1.28663000

H 7.49905900 0.51472700 -0.60836900

C 2.24383100 -0.72162600 -4.65769300

H 0.80214300 0.89252500 -4.73451600

H 3.67172300 -2.29720900 -4.25392500

H 2.92118500 5.75131200 1.24280500

H 7.98719400 -0.47302700 1.63205400

H 2.13636200 -0.97761900 -5.71467100

**TS14**

C -4.83170600 3.38245700 0.50070100

C -5.45172300 2.19618300 0.92637600

C -5.31997000 1.01116800 0.20172200

C -4.56116000 1.01556200 -0.97010000

C -3.94597600 2.18416400 -1.43166800

C -4.08105100 3.35655000 -0.68828800

H -6.04078600 2.19669600 1.84742600

H -5.78728000 0.08463700 0.53839200

H -3.37784400 2.16939800 -2.36364200

H -3.59628300 4.27136800 -1.04003400

C -4.99185600 4.66505600 1.27769300

H -4.07229700 5.26999000 1.25114200

H -5.24982200 4.47369600 2.33014500

H -5.79933100 5.28279700 0.84737000

S -4.25397200 -0.52586500 -1.82611000

O -4.01386100 -0.21247800 -3.23553500

O -5.26566000 -1.49563100 -1.39430200

N -2.80005900 -1.05290100 -1.20791300

C -1.52151600 -0.64274000 -1.79287000

H -1.45660500 -0.95803600 -2.84674200

H -1.40798700 0.45494700 -1.76227600

C -0.45139000 -1.34390600 -0.93804700

H -0.33307700 -0.84147300 0.03219000

C 0.86057800 -1.60120000 -1.57675000

H 0.79970300 -1.97527000 -2.61301600

H 1.53074100 -2.23653200 -0.98058500

C -2.70520100 -2.01929700 -0.11870300

H -3.52181000 -2.74520200 -0.22394000

C -2.78983800 -1.40458600 1.27300400

C -3.20011500 -2.19658100 2.35590500

C -2.47521800 -0.05860100 1.50690800

C -3.28157300 -1.66254300 3.64483900

H -3.47317900 -3.24200800 2.18484100

C -2.55829500 0.48073200 2.79299100

H -2.19043400 0.58404200 0.67291400

C -2.95966100 -0.31921700 3.86747700

H -3.60838800 -2.29333200 4.47543100

H -2.31870300 1.53507200 2.95101000

H -3.03067100 0.10376700 4.87267300

C -1.33893900 -2.67826300 -0.44034800

C -1.42608000 -3.82978600 -1.49725100

C -0.66542200 -3.61811400 0.58212700

C -1.45168100 -4.57700700 -0.22772800

H -0.47899200 -3.86579000 -2.06820300

H -2.29746600 -3.88016800 -2.16502700

H 0.43098000 -3.61639400 0.42781000

H -0.89370400 -3.50911100 1.64902800

H -2.31680400 -5.13419200 0.14376300

C 2.17365300 -0.86660200 1.36942400

C 2.81694900 -2.09225000 1.60154300

H 3.69638200 -2.36690900 1.01568100

C 2.34643800 -2.96157300 2.59347000

H 2.86192500 -3.90948700 2.76822100

C 1.22831000 -2.61941900 3.35809300

H 0.86328300 -3.29868300 4.13267200

C 0.56908400 -1.40754700 3.12068000

H -0.32061800 -1.13974700 3.69374800

C 1.03635300 -0.54141100 2.13204400

H 0.51640400 0.40391200 1.95854000

C 4.40597500 -0.28907600 -0.38678100

C 4.60319200 -1.00204100 -1.58020200

H 3.75881900 -1.15083900 -2.25766000

C 5.86920300 -1.49864400 -1.90477700

H 6.01166200 -2.04804700 -2.83875000

C 6.94969000 -1.28218000 -1.04507600

H 7.94087000 -1.66463900 -1.30240800

C 6.76175800 -0.56474100 0.14147200

H 7.60522400 -0.38433200 0.81292300

C 5.49779700 -0.06940200 0.47010400

H 5.36666300 0.49578100 1.39488300

C 2.99428000 1.86837500 0.97225700

C 2.99360000 3.09243200 0.28253700

C 3.26537100 1.86063300 2.35372300

C 3.26456900 4.28218900 0.96453100

H 2.77518600 3.11515600 -0.78781200

C 3.53164700 3.05451500 3.02956400

H 3.26890700 0.92218300 2.91111400

C 3.53208300 4.26881700 2.33648400

H 3.26063700 5.22683400 0.41474200

H 3.73913900 3.03308500 4.10269800

H 3.73830600 5.20256100 2.86635100

Cl 2.02787500 2.26123100 -2.98344900

Rh 1.38046800 0.41171400 -1.69902300

P 2.70396700 0.29884100 0.03322600

**15**

C -5.09298300 3.27947500 0.46068700

C -5.64948300 2.06226500 0.88711800

C -5.44526700 0.88332000 0.16979700

C -4.67628200 0.92417700 -0.99481200

C -4.12225900 2.12266300 -1.45609300

C -4.33047200 3.28965600 -0.72076600

H -6.24607100 2.03420500 1.80290600

H -5.86405200 -0.06606100 0.50661500

H -3.54420700 2.13399800 -2.38205400

H -3.89499600 4.22876300 -1.07309000

C -5.33513000 4.55413900 1.22955700

H -4.46969900 5.23203700 1.17119800

H -5.54722700 4.35435400 2.29079000

H -6.20305300 5.09828300 0.81778700

S -4.27731600 -0.60216900 -1.84111900

O -4.03089900 -0.28099000 -3.24800000

O -5.24901500 -1.61739400 -1.42195000

N -2.80947900 -1.05329300 -1.19915000

C -1.53869000 -0.63544000 -1.79716700

H -1.46800000 -0.97775600 -2.84214600

H -1.43970500 0.46393600 -1.79613600

C -0.46903900 -1.30928800 -0.91599900

H -0.34996900 -0.74837600 0.02480100

C 0.86403000 -1.53871400 -1.55017200

H 0.81240000 -1.91508600 -2.58635900

H 1.52490400 -2.18311100 -0.95148800

C -2.68402700 -1.99384300 -0.08903700

H -3.47036500 -2.75500600 -0.17682800

C -2.79505100 -1.34816500 1.28642900

C -3.18605500 -2.12496600 2.38730000

C -2.52662400 0.01322700 1.48770000

C -3.29601700 -1.56026900 3.66109700

H -3.42237600 -3.18304100 2.24140700

C -2.63857000 0.58321300 2.75819800

H -2.25843600 0.64268900 0.63826500

C -3.02231400 -0.20133300 3.85054200

H -3.60803100 -2.17989900 4.50571200

H -2.43659200 1.64899600 2.89002800

H -3.11674500 0.24534700 4.84351600

C -1.29310100 -2.60644900 -0.40215600

C -1.37234300 -3.80292600 -1.42044700

C -0.62564500 -3.53707800 0.63861100

C -1.32767900 -4.56945800 -0.16165600

H -0.43913700 -3.81235800 -2.01926300

H -2.24926700 -3.89916200 -2.07824400

H 0.47697900 -3.49372900 0.52045800

H -0.87145300 -3.42739400 1.70327900

H -2.09192500 -5.26228000 0.20232000

C 2.16978900 -0.79160500 1.40764800

C 2.77864900 -2.02240200 1.69902200

H 3.65913700 -2.34283500 1.13872300

C 2.27296000 -2.83765400 2.71924300

H 2.76188400 -3.78994900 2.94022300

C 1.15226600 -2.43750300 3.45083000

H 0.75847000 -3.07496500 4.24647700

C 0.52579000 -1.22154000 3.15260600

H -0.36660200 -0.91105000 3.69895700

C 1.02868600 -0.40815900 2.13738800

H 0.53360600 0.54062200 1.91553500

C 4.42102500 -0.36637300 -0.37196100

C 4.58174100 -1.11156900 -1.55098100

H 3.73065700 -1.23642300 -2.22453500

C 5.82324200 -1.67116700 -1.86776800

H 5.93808300 -2.24516500 -2.79065200

C 6.91482200 -1.48658600 -1.01470100

H 7.88684200 -1.91879700 -1.26622000

C 6.76327200 -0.73758400 0.15753200

H 7.61599300 -0.58229800 0.82350800

C 5.52412800 -0.17873000 0.47828600

H 5.42049500 0.41056100 1.39160200

C 3.12392300 1.89096100 0.93276300

C 3.21070700 3.08589600 0.19849000

C 3.37151900 1.91714400 2.31803600

C 3.54456000 4.28107200 0.84153000

H 3.00955600 3.08265200 -0.87546900

C 3.70097800 3.11693200 2.95465700

H 3.30777300 1.00124800 2.90833400

C 3.78857000 4.30195200 2.21794900

H 3.60810500 5.20318300 0.25824200

H 3.88928100 3.12309100 4.03149100

H 4.04403200 5.24029800 2.71729400

Cl 2.15601200 2.20426200 -3.05617200

Rh 1.42157000 0.45622200 -1.68152600

P 2.75318900 0.31127200 0.04349400

**TS5***

C -6.77419400 1.78913800 1.30716300

C -5.74118800 2.67962300 0.97564000

C -4.78275500 2.35211300 0.01366400

C -4.85246100 1.10894000 -0.61955500

C -5.88756500 0.21259200 -0.33044800

C -6.83645100 0.55747600 0.63017900

H -5.68982200 3.65300600 1.47083500

H -4.00023600 3.05880500 -0.26760700

H -5.95501200 -0.73035000 -0.87589000

H -7.65048400 -0.13800900 0.85304200

C -7.79087200 2.13100400 2.36690600

H -7.80976400 3.20966600 2.58145300

H -8.80422800 1.82148400 2.06659300

H -7.55905200 1.60850900 3.31127900

S -3.59477700 0.64187500 -1.81010500

O -2.98499000 1.87890500 -2.30066800

O -4.19746400 -0.33701900 -2.72390900

N -2.41839400 -0.18338300 -0.93776700

C -1.58347800 0.59944100 -0.02720900

H -2.13011200 1.52680200 0.21685600

H -1.50758000 0.05183000 0.92502100

C -0.19619400 0.99308700 -0.48429500

H 0.25069000 1.62501500 0.29155300

C 0.25501800 1.17341700 -1.80800600

H -0.42186700 0.97297600 -2.63976700

H 1.01337200 1.93492400 -2.01453300

C -2.42822900 -1.65505000 -0.93559700

H -3.41559800 -1.94921900 -1.32560800

C -2.33376300 -2.22435100 0.47356100

C -3.50599400 -2.23147300 1.24883700

C -1.14076300 -2.66790700 1.05783400

C -3.48923400 -2.67360400 2.57241700

H -4.44175100 -1.87252500 0.81360100

C -1.12446600 -3.12190900 2.38175200

H -0.20286000 -2.65056700 0.50287200

C -2.29407400 -3.12501200 3.14448100

H -4.41227600 -2.66912800 3.15784300

H -0.18050800 -3.46942300 2.80958100

H -2.27825900 -3.47720700 4.17921500

Rh 1.10859200 -0.59655300 -0.97218500

Cl 2.13575200 -2.71648700 -0.38180500

P 2.75678500 0.44479900 0.25096500

C 2.96874400 2.25129800 -0.10871900

C 2.53208500 0.38412800 2.08336400

C 4.45778400 -0.20899100 -0.06189200

C 2.25391400 3.23912500 0.59172100

C 3.75110000 2.64481200 -1.20913500

C 1.67423800 -0.59974700 2.60202200

C 3.17088400 1.26761200 2.97262300

C 4.73413400 -0.74987000 -1.32853800

C 5.48566100 -0.14560100 0.89044800

H 1.64478800 2.96805700 1.45609000

C 2.32347800 4.58113900 0.20544800

C 3.82031900 3.98568800 -1.59316500

H 4.31424300 1.89845100 -1.77284100

H 1.18400900 -1.29240600 1.91809600

C 1.45543200 -0.69565100 3.97879600

C 2.95383500 1.16688300 4.34952900

H 3.83092000 2.04918300 2.59004700

C 6.01966100 -1.19525300 -1.64375400

H 3.92970200 -0.84049400 -2.06124400

C 6.76899200 -0.60314200 0.57702800

H 5.29124500 0.24830400 1.88880100

H 1.76158800 5.33318500 0.76542500

C 3.10582000 4.95960200 -0.88820300

H 4.43724100 4.27023100 -2.44941100

H 0.77611400 -1.46126300 4.36201400

C 2.09399600 0.18598200 4.85553400

H 3.45535400 1.86086400 5.02915700

C 7.04091500 -1.12323600 -0.69150000

H 6.21926500 -1.61479400 -2.63305900

H 7.55859600 -0.55503500 1.33147200

H 3.15956200 6.00858900 -1.19020200

H 1.92067400 0.11275000 5.93243100

H 8.04488100 -1.48106000 -0.93441700

C -1.51286100 -2.21167000 -2.01315600

C -0.76616200 -3.41164700 -2.08127700

C -1.23593000 -1.53273900 -3.31286600

C -0.05808500 -1.78891000 -2.39812800

H -0.70357900 -3.96465600 -3.02161900

H -0.42712000 -3.92229600 -1.18008400

H -1.34685300 -2.13908000 -4.22178000

H -1.57684500 -0.50319300 -3.41789100

H 0.89246600 -2.23417000 -2.71115200

**TS5-model**

C -3.77990700 1.25712500 -0.40572500

C -3.47698600 0.17089200 -1.24141800

C -2.49676500 -0.76088700 -0.89229300

C -1.80003000 -0.59599100 0.30669800

C -2.09342700 0.46539800 1.17019600

C -3.07645100 1.38369100 0.80594100

H -4.02214200 0.04599500 -2.18085700

H -2.28164400 -1.61987900 -1.53002500

H -1.56159600 0.55540000 2.11884200

H -3.30839600 2.21463900 1.47806000

C -4.85622300 2.24827200 -0.77044700

H -5.11791200 2.19234800 -1.83732200

H -5.77599700 2.05565300 -0.19149100

H -4.54373700 3.28029600 -0.54591500

S -0.48619100 -1.73834100 0.73207600

O -0.74321800 -2.98054200 0.00020400

O -0.35630200 -1.72574600 2.19842200

N 0.90756400 -1.04105500 0.11940300

C 1.17777800 -1.14098600 -1.31117600

H 0.23394300 -1.40307000 -1.81727900

H 1.46077300 -0.14045300 -1.67729600

C 2.23630000 -2.14807000 -1.69663400

H 2.41488200 -2.10821200 -2.77633000

C 2.45842600 -3.37069600 -1.02596300

H 1.86088700 -3.60661800 -0.14131400

H 2.78267800 -4.24835400 -1.59329400

C 1.90739800 -0.44460300 1.01749700

H 1.37321400 -0.29525700 1.96483700

C 2.30826000 0.94532300 0.52292500

C 1.27795500 1.87998100 0.31806100

C 3.62761400 1.33552000 0.26492600

C 1.55580200 3.16858600 -0.13789300

H 0.24209300 1.58480300 0.49982500

C 3.90725100 2.63074100 -0.19130100

H 4.45211000 0.63558600 0.39043400

C 2.87792300 3.55045800 -0.39700600

H 0.73746300 3.87636100 -0.29472700

H 4.94545300 2.90876600 -0.39184500

H 3.09966100 4.55829800 -0.75778700

H 2.70102000 -1.18369100 1.12710500

**TS5*-model**

C -3.69902300 1.23069700 -0.50224000

C -3.36244500 0.14235700 -1.32212000

C -2.39694700 -0.78834800 -0.93115100

C -1.74967700 -0.62178100 0.29547100

C -2.08499400 0.43726000 1.14662200

C -3.05258900 1.35457400 0.74112300

H -3.87188900 0.01263400 -2.28074900

H -2.16059400 -1.65267600 -1.55399700

H -1.60656700 0.51812700 2.12425300

H -3.32171300 2.17911400 1.40724900

C -4.71969000 2.25142600 -0.93803200

H -5.34934000 1.87477100 -1.75752700

H -5.37733900 2.54362100 -0.10436500

H -4.22290100 3.16990700 -1.29604600

S -0.46080600 -1.76809600 0.78750800

O -0.67089800 -3.00353000 0.03100300

O -0.40437200 -1.76146000 2.25491200

N 0.98361500 -1.09438200 0.25303900

C 1.22974900 -1.08782400 -1.18862300

H 0.25355400 -1.17247200 -1.69680900

H 1.61295900 -0.09374200 -1.46670900

C 2.13978800 -2.14907900 -1.76655800

H 2.15241500 -2.02946900 -2.85582900

C 2.39580900 -3.43666500 -1.25187600

H 1.94749500 -3.73657600 -0.30359100

H 2.58166600 -4.26195100 -1.94614400

C 1.81597800 -0.33775100 1.20203100

H 1.17012300 -0.13273700 2.07058300

C 2.23074400 1.01674300 0.64340000

C 1.27923900 2.05048300 0.67873200

C 3.46835300 1.26505000 0.03674100

C 1.55686200 3.30159300 0.12621600

H 0.30239000 1.86804800 1.13304200

C 3.75237700 2.52265300 -0.50815200

H 4.22356600 0.48292700 -0.03995200

C 2.80062200 3.54361000 -0.46869700

H 0.80165100 4.09091900 0.16324800

H 4.72936500 2.69049800 -0.96878400

H 3.02362600 4.52404800 -0.89762200

H 2.57635700 -0.97486600 1.65370400

**TS5-model-opt**

C -3.83915100 0.22821300 -0.05287100

C -3.15665400 -0.11896700 -1.23469900

C -1.95761200 -0.82584000 -1.19452100

C -1.42518800 -1.18942700 0.04725500

C -2.08688800 -0.87806200 1.23487700

C -3.28828700 -0.16590600 1.17563300

H -3.57639800 0.17145100 -2.20192400

H -1.43019600 -1.09652300 -2.11107600

H -1.66041400 -1.19519600 2.18802800

H -3.80673000 0.08669700 2.10442700

C -5.14569600 0.97801100 -0.12002400

H -5.10382900 1.79125800 -0.86169800

H -5.96589200 0.30506100 -0.42446700

H -5.41700700 1.41311700 0.85304100

S 0.17059500 -2.00527200 0.09728400

O 0.28519700 -2.87328200 -1.07696100

O 0.33980800 -2.52824300 1.46023200

N 1.23469800 -0.72844600 -0.15114700

C 2.24361900 -0.81527000 -1.20102200

H 1.80113100 -1.32623600 -2.07103700

H 2.45975500 0.21766600 -1.52677800

C 3.52812500 -1.51040900 -0.81473800

H 4.35975400 -1.35244200 -1.51337100

C 3.70081200 -2.28988700 0.25525100

H 2.88875400 -2.48507800 0.96266700

H 4.66063000 -2.77549000 0.45060100

C 1.46590600 0.20240100 0.95682200

H 0.72249400 -0.00963500 1.73887500

C 1.35618600 1.64773200 0.51457500

C 0.15222900 2.13413200 -0.02150600

C 2.44938000 2.51657700 0.62046300

C 0.04597000 3.46297200 -0.43405700

H -0.70346200 1.46245900 -0.11759500

C 2.34507600 3.85045000 0.20823600

H 3.39377000 2.14458400 1.02853200

C 1.14282700 4.32646400 -0.31974300

H -0.89805000 3.82896100 -0.84645200

H 3.20758500 4.51613900 0.29675700

H 1.05881800 5.36697500 -0.64390000

H 2.45292300 0.01278700 1.41109200

**TS8***

C -5.81301600 2.34995700 -0.17646200

C -5.72354200 1.62346700 -1.37530300

C -5.27754000 0.30072100 -1.38620000

C -4.91226700 -0.29951700 -0.17909900

C -5.01856100 0.38801200 1.03272400

C -5.47002300 1.70568700 1.02440400

H -6.00637000 2.10143300 -2.31704400

H -5.21506800 -0.26637200 -2.31699200

H -4.73215600 -0.10754600 1.96094500

H -5.54165400 2.25257100 1.96824500

C -6.23543700 3.79697100 -0.16914600

H -6.94234800 4.00591200 0.64927400

H -5.35965400 4.45111900 -0.01497300

H -6.70867300 4.09150100 -1.11735300

S -4.15356400 -1.92068200 -0.20248500

O -4.71043600 -2.67197600 -1.33026400

O -4.17424100 -2.44018700 1.16637500

N -2.54036000 -1.63664300 -0.58676500

C -2.09751400 -1.60218400 -1.98607800

H -2.69420000 -2.33876900 -2.54286600

H -2.24294800 -0.61588600 -2.46246800

C -0.63586400 -1.98045000 -1.99437100

H -0.45943200 -3.02579800 -1.72686000

C 0.36440600 -1.38341200 -2.82386200

H 0.06825100 -0.64097200 -3.57090700

H 1.24456100 -1.98090700 -3.08082100

C -1.61647200 -1.16223100 0.45662000

H -1.87988500 -1.71672700 1.36942200

C -1.69180800 0.32938500 0.78333200

C -1.39776700 0.73887600 2.09362800

C -2.04324600 1.30301300 -0.16281900

C -1.45764700 2.08701800 2.45317800

H -1.12497500 -0.01197000 2.84096800

C -2.10280700 2.65202900 0.19362400

H -2.29151600 1.01730500 -1.18265600

C -1.81587000 3.04884700 1.50239400

H -1.22601900 2.38804500 3.47791500

H -2.37245200 3.39300000 -0.56186600

H -1.86699200 4.10428800 1.78135800

C -0.20329000 -1.60006800 0.01962900

C 0.29692200 -2.87740900 0.65251800

H 0.72475900 -2.55360000 1.61989400

H 1.14977400 -3.25400300 0.06527700

Rh 0.65229600 -0.26875900 -1.05344300

P 2.63568000 0.23575400 -0.02875300

C 2.88660000 -0.80386000 1.47577300

C 3.43476000 -2.09480200 1.38642600

H 3.82228400 -2.45800200 0.43197600

C 3.47953500 -2.92751100 2.50796800

H 3.91455900 -3.92647700 2.42244400

C 2.95584900 -2.49253300 3.72931300

H 2.98429800 -3.14684100 4.60409800

C 2.39231600 -1.21527600 3.82282400

H 1.97884200 -0.86709400 4.77293400

C 2.36193200 -0.37383600 2.70712100

H 1.92017700 0.62148300 2.79267900

C 4.15007000 -0.01680700 -1.04941500

C 4.06341900 0.16210900 -2.44098100

H 3.10854100 0.44390100 -2.89088900

C 5.20250400 0.01701500 -3.23826400

H 5.12292500 0.16347500 -4.31845900

C 6.43408700 -0.30888900 -2.66320900

H 7.32115000 -0.42507000 -3.29139400

C 6.52794800 -0.48314600 -1.27891800

H 7.48762500 -0.73421000 -0.81967900

C 5.39459400 -0.33663800 -0.47505000

H 5.48554300 -0.48139900 0.60321900

C 2.79065000 1.94814900 0.64104300

C 4.00832000 2.44326300 1.13680600

C 1.64224100 2.74970800 0.70647500

C 4.07211600 3.72048100 1.69696300

H 4.91405000 1.83605000 1.08326400

C 1.70852700 4.02516800 1.27526900

H 0.69996600 2.38364300 0.29882300

C 2.92029700 4.51313200 1.76973100

H 5.02454300 4.09949900 2.07656700

H 0.80476800 4.63707200 1.32141700

H 2.97216800 5.51358600 2.20747800

Cl 0.91579400 1.68507400 -2.52131100

C -0.72398100 -3.95881200 0.89441100

C -1.09887200 -4.39156900 2.10075700

H -1.87096300 -5.15591100 2.22036800

H -0.65258000 -3.98504700 3.01504300

H -1.21039000 -4.37931100 0.00585200

**TS8-model**

C 6.77552000 -0.39644200 -0.37919500

C 6.25358200 -1.58375900 0.15920300

C 4.92884500 -1.95988100 -0.07118300

C 4.11545100 -1.13043900 -0.84686700

C 4.61335900 0.04421500 -1.41677700

C 5.93817000 0.40094300 -1.17830900

H 6.89542500 -2.22781000 0.76629900

H 4.53022900 -2.89117900 0.33553600

H 3.95779000 0.66970700 -2.02220500

H 6.32788000 1.32577300 -1.61161100

C 8.19383500 0.03405600 -0.10299400

H 8.72490500 0.28159300 -1.03629300

H 8.20862500 0.94093400 0.52559200

H 8.76585300 -0.74656000 0.41942000

S 2.37784200 -1.52085000 -1.01594600

O 2.24047800 -2.98034900 -1.03402400

O 1.83492600 -0.68290200 -2.09335600

N 1.64012500 -0.99391300 0.39050300

C 1.70328100 -1.76498500 1.62438700

H 1.94815500 -2.80491300 1.36086100

H 2.49287300 -1.39826900 2.30330800

C 0.34613000 -1.72132000 2.29125400

H 0.34986900 -1.80200300 3.38325200

C -0.76747000 -2.24186500 1.55888300

H -0.58739600 -2.53749800 0.52133400

H -1.51128400 -2.83937100 2.09213500

C 1.23332500 0.40649900 0.54220700

H 0.69480100 0.68592400 -0.37549000

C 2.40441400 1.37747600 0.66844400

C 3.44080300 1.21029000 1.60123900

C 2.46698800 2.46767400 -0.21063600

C 4.51255400 2.10325200 1.64624000

H 3.42537500 0.36907500 2.29458500

C 3.53429300 3.36987500 -0.16229100

H 1.67515800 2.60107800 -0.95029000

C 4.56336700 3.18894100 0.76499700

H 5.31475000 1.94865100 2.37220400

H 3.56372400 4.21127300 -0.85954600

H 5.40276700 3.88798200 0.80148000

C 0.17730100 0.45785600 1.65004300

C 0.35763900 1.49571500 2.73764400

H 0.66112700 2.44860100 2.26873000

H 1.19143500 1.22331600 3.41109300

Cl -3.52698800 -1.36362900 2.86426800

C -1.66421800 1.49059800 -1.10360100

C -2.54256200 -1.32620900 -1.29869300

C -4.28211000 0.78208100 -0.30268200

C -1.13318900 1.37730500 -2.39551500

C -1.54661600 2.71964800 -0.42637800

C -3.71328800 -2.05554000 -1.55854800

C -1.31051400 -1.82029800 -1.76163500

C -5.09976600 0.85004300 0.83373100

C -4.77344200 1.23538900 -1.54151900

H -1.20690200 0.43897100 -2.94504700

C -0.49457500 2.47088200 -2.99610300

C -0.92425900 3.81067100 -1.03027700

H -1.95357700 2.81924900 0.58341600

H -4.67630600 -1.69865400 -1.18847500

C -3.64628800 -3.25067800 -2.28225400

C -1.24647600 -3.01179700 -2.48403800

H -0.38734200 -1.28131900 -1.55890500

C -6.39618400 1.36941700 0.72757100

H -4.73358800 0.46007000 1.78656400

C -6.06664100 1.74856700 -1.64047700

H -4.14055700 1.19077000 -2.43145800

H -0.07515200 2.36069800 -3.99904800

C -0.39299200 3.68823300 -2.32153700

H -0.84735900 4.75881500 -0.49221100

H -4.56302900 -3.81317000 -2.47692400

C -2.41776400 -3.73086000 -2.74677000

H -0.27135300 -3.37521400 -2.81686200

C -6.88055100 1.81894500 -0.50252800

H -7.03024900 1.41409300 1.61680000

H -6.44032100 2.09805000 -2.60651900

H 0.10263800 4.53996700 -2.79423100

H -2.37232900 -4.66902100 -3.30584600

H -7.89343300 2.22335400 -0.57970400

Rh -1.56434500 -0.31736700 1.78701300

P -2.55332400 0.13894700 -0.19418800

H -0.57832200 1.61606900 3.28316400

**TS8*-model**

C -5.89671100 1.97379400 -0.61314100

C -5.79982000 0.96181400 -1.58243000

C -5.32602200 -0.30949500 -1.25382300

C -4.94016000 -0.56751200 0.06350000

C -5.05295600 0.40992900 1.05566700

C -5.53223300 1.67123300 0.70989300

H -6.09891600 1.17249400 -2.61270900

H -5.25772600 -1.09827100 -2.00551400

H -4.75000100 0.17881200 2.07743200

H -5.60917200 2.44373100 1.47955300

C -6.34958600 3.36456300 -0.97784400

H -7.05533100 3.76629300 -0.23371500

H -5.48683500 4.05254300 -1.00975300

H -6.83520600 3.39293200 -1.96433500

S -4.14750800 -2.12428300 0.45403600

O -4.69581000 -3.15377400 -0.43250100

O -4.14813900 -2.26943600 1.91109500

N -2.54324900 -1.92008000 -0.01011000

C -2.11049400 -2.24316900 -1.37528900

H -2.69513600 -3.11041800 -1.71393000

H -2.27990500 -1.41798100 -2.09030400

C -0.64125700 -2.58312900 -1.30218400

H -0.44098900 -3.51916700 -0.77382000

C 0.34065300 -2.20427000 -2.27036200

H 0.02392700 -1.68781700 -3.18138400

H 1.23153100 -2.83151600 -2.37332400

C -1.62272300 -1.17298300 0.86268200

H -1.86830600 -1.47529000 1.89144800

C -1.72742900 0.35055700 0.79059100

C -1.43345700 1.09276100 1.94546400

C -2.10563200 1.03725300 -0.37219600

C -1.51946000 2.48665500 1.94225100

H -1.13990800 0.56783300 2.85923700

C -2.19135500 2.43118700 -0.37863900

H -2.35454200 0.49107600 -1.27942800

C -1.90422700 3.16062800 0.77814500

H -1.28747900 3.04858500 2.85042300

H -2.48160200 2.94451800 -1.29774700

H -1.97581600 4.25115200 0.77324500

C -0.20351600 -1.68306200 0.53799700

C 0.32777600 -2.74177100 1.47566600

H 0.75505600 -2.16912900 2.32019300

H 1.18450000 -3.24236300 0.99664400

Rh 0.61660400 -0.66161000 -0.85481800

P 2.59560600 0.12948100 -0.02051500

C 2.87840300 -0.47728800 1.69972100

C 3.45314300 -1.73642900 1.94309400

H 3.84194200 -2.32848000 1.11160400

C 3.52293700 -2.24713100 3.24216200

H 3.97841800 -3.22555600 3.41454200

C 2.99825600 -1.51879400 4.31424900

H 3.04632300 -1.92189600 5.32883000

C 2.40846400 -0.27206400 4.07864300

H 1.99401000 0.30387400 4.91016200

C 2.35291100 0.24885600 2.78276800

H 1.89077800 1.22364300 2.61144900

C 4.10821600 -0.35195500 -0.95814200

C 4.00860000 -0.54348700 -2.34715800

H 3.04502000 -0.40657300 -2.84353100

C 5.14520100 -0.86999900 -3.09264700

H 5.05540100 -1.01159000 -4.17269000

C 6.38717800 -1.01170800 -2.46725900

H 7.27232600 -1.27096000 -3.05402700

C 6.49385600 -0.81744000 -1.08656500

H 7.46164500 -0.92217300 -0.58924200

C 5.36299800 -0.48774100 -0.33515500

H 5.46411700 -0.34479900 0.74245100

C 2.71874700 1.95984000 0.17838900

C 3.92892400 2.58971300 0.51360500

C 1.55409900 2.72913900 0.04650000

C 3.96939400 3.96973400 0.72106100

H 4.84692100 2.00657200 0.60925400

C 1.59715200 4.10975900 0.26272200

H 0.61710200 2.25194200 -0.24053100

C 2.80157300 4.73228200 0.59861500

H 4.91608000 4.45231500 0.97752000

H 0.68097700 4.69553900 0.15869000

H 2.83516500 5.81302900 0.76010200

Cl 0.82900300 0.84680000 -2.78383800

H -0.39288900 -3.46513000 1.85710300

**16**

C -5.82260000 2.28425600 -0.72940800

C -6.42239700 1.01577800 -0.76272400

C -5.74882300 -0.08922300 -1.28451400

C -4.45771100 0.07822000 -1.78314400

C -3.84349300 1.33312200 -1.79267800

C -4.52669800 2.42439000 -1.25736400

H -7.42718900 0.88344200 -0.35251500

H -6.19663100 -1.08348600 -1.27932800

H -2.83897100 1.44711300 -2.20221800

H -4.04617900 3.40726200 -1.25212500

C -6.56304800 3.47160900 -0.16565500

H -7.19563200 3.18350400 0.68845400

H -5.87270400 4.26168100 0.16784200

H -7.22760400 3.91760800 -0.92613400

S -3.53866800 -1.34955900 -2.37491600

O -2.90115800 -0.95482700 -3.63332500

O -4.44131400 -2.50528300 -2.29436200

N -2.31170600 -1.58556000 -1.24702000

C -0.93397400 -1.22736200 -1.61313100

H -0.26860100 -1.95282900 -1.13306800

H -0.78346500 -1.33878200 -2.69263200

C -0.58234000 0.18073500 -1.16774000

H -1.06012000 0.46224300 -0.22477100

H 0.06549200 0.95952500 -3.07565900

H -0.23269300 2.23679100 -1.76262200

C -2.61865300 -2.46343100 -0.08873300

H -2.95979500 -3.43663300 -0.47791400

C -3.74002600 -1.91071100 0.78264500

C -4.81974300 -2.73749400 1.11997800

C -3.72534800 -0.59196700 1.25830200

C -5.86207400 -2.26403300 1.92181500

H -4.85499000 -3.75651100 0.72618400

C -4.76687900 -0.11552300 2.05626800

H -2.91601500 0.08008800 0.97142200

C -5.83788800 -0.94837700 2.39328600

H -6.70092500 -2.92070000 2.16695600

H -4.75182400 0.92098100 2.39547900

H -6.65753100 -0.56908800 3.00917800

C -1.37257100 -2.73255500 0.70950500

C -0.52803700 -3.97531600 0.76969200

C -0.71790500 -1.97757100 1.82868600

C -1.15153900 -3.40283400 2.00860300

H 0.55875100 -3.90937200 0.62446100

H -0.98642500 -4.88227200 0.35713100

H 0.35200000 -1.73510800 1.74270400

H -1.31828100 -1.20792900 2.32239500

H -1.87243100 -3.83112500 2.70485300

C 2.88041500 1.46318700 2.01296300

H 3.75230700 1.98157600 1.58251100

H 2.67396700 1.92799700 2.98914900

C 3.15393100 -0.03793700 2.11663600

H 2.32706900 -0.55393800 2.62931000

H 4.08500400 -0.24616800 2.66644700

C -0.02764300 1.94054900 1.85082600

C -1.15076000 2.60524800 1.32320800

H -1.09752500 3.06547400 0.33378900

C -2.33424300 2.69734200 2.05788800

H -3.19885200 3.20734100 1.62627400

C -2.41368500 2.12995100 3.33391700

H -3.33489600 2.20811500 3.91521600

C -1.31186100 1.45097100 3.85963900

H -1.37158600 0.99342100 4.85018500

C -0.12844900 1.35224900 3.12171900

H 0.71520100 0.80935500 3.55183000

C 1.79124900 3.52901100 0.29817300

C 2.24656100 3.79206900 -1.00387600

H 2.35039600 2.96545600 -1.71291700

C 2.54849000 5.10072800 -1.39429600

H 2.89719000 5.29530800 -2.41159000

C 2.39879800 6.15481500 -0.48860100

H 2.63289600 7.17767100 -0.79477100

C 1.94314000 5.89997500 0.81021100

H 1.82131100 6.72272800 1.51951300

C 1.63892400 4.59439500 1.20227300

H 1.27158200 4.40513300 2.21441600

C 3.19332600 -2.55615900 0.59452100

C 3.24228700 -3.17411100 1.85601600

H 3.31523900 -2.58225400 2.76976600

C 3.19672300 -4.56805700 1.96255400

H 3.22723600 -5.03562300 2.94990700

C 3.11081500 -5.35755200 0.81320700

H 3.07298000 -6.44653300 0.89831500

C 3.06434400 -4.74745700 -0.44522800

H 2.98699200 -5.35772200 -1.34848900

C 3.09938000 -3.35640600 -0.55925100

H 3.02698900 -2.88082700 -1.54094100

C 4.98179800 -0.36790700 -0.08795300

C 6.05340300 -1.18000900 0.31806400

H 5.86263800 -2.09349300 0.88718700

C 7.36471100 -0.83150300 -0.01484000

H 8.19278900 -1.47221900 0.29928100

C 7.61768200 0.32965900 -0.75370000

H 8.64420300 0.59776500 -1.01666700

C 6.55494800 1.13962700 -1.16451700

H 6.74596300 2.04127700 -1.75176800

C 5.24140400 0.78975000 -0.83942200

H 4.40853800 1.41021200 -1.17985600

Cl 2.15827200 -0.93665600 -2.85332800

P 1.48259000 1.78081700 0.80494900

P 3.23829900 -0.73284500 0.37922900

C -0.11454200 1.17993200 -2.02043000

Rh 1.58720400 0.29678800 -0.87358400

**17**

Rh -1.04949600 0.14935200 -0.60422000

Cl 0.53863000 1.16694300 -2.10130500

C 0.96583500 -2.37702600 -1.31422100

C 0.72759300 -2.33909400 -2.79129600

C -0.14052400 -3.31732500 -0.93995100

C -0.40741700 -2.13252900 -1.82557800

H 0.75942900 -3.27479600 -3.37024400

H 1.11440000 -1.45395800 -3.30350000

H -0.15595100 -4.33107100 -1.37151500

H -0.50589100 -3.25629100 0.08568000

H -1.22221000 -1.37984600 -1.86188600

P -2.84003400 -0.72135900 0.43454800

P -1.54629500 2.03708500 0.45378900

C -3.73587400 0.63135000 1.38003000

C -2.66314600 -2.08387700 1.67077800

C -4.18832000 -1.33932000 -0.67104400

C -2.73415600 1.68102900 1.85042500

C -0.23668500 3.06127000 1.25075000

C -2.45036100 3.28407500 -0.56424800

H -4.44419900 1.07188100 0.66084800

H -4.32183000 0.20347500 2.20764500

C -2.35831400 -1.79364000 3.01090000

C -2.73756100 -3.43129000 1.27459500

C -4.14794300 -1.03098800 -2.04015900

C -5.30048200 -2.03315000 -0.16086600

H -2.12028700 1.29882800 2.68034700

H -3.23298700 2.60269200 2.18703600

C -0.32651100 3.50009500 2.58292400

C 0.88062500 3.43377800 0.48414000

C -2.89055300 4.49225400 0.00370500

C -2.70936600 3.02392900 -1.91790000

H -2.26532500 -0.75946900 3.34629400

C -2.15122500 -2.82128400 3.93405800

C -2.51607500 -4.45832600 2.19521300

H -2.97385500 -3.68273300 0.23868500

H -3.29832500 -0.47174600 -2.44047800

C -5.19096900 -1.42105500 -2.88628200

C -6.34287900 -2.41848700 -1.00655500

H -5.35022800 -2.28247400 0.90191700

H -1.17772000 3.23083700 3.20909100

C 0.68418300 4.29018700 3.14041000

C 1.88149100 4.22910500 1.04319600

H 0.97342100 3.07465500 -0.54332000

H -2.66497600 4.72316700 1.04871800

C -3.60030600 5.41536200 -0.76630000

C -3.41589300 3.95365200 -2.68813100

H -2.33543000 2.09842200 -2.36134900

H -1.91947400 -2.57438800 4.97320300

C -2.22501700 -4.15713800 3.52947400

H -2.57549900 -5.49928300 1.86746800

H -5.14287600 -1.17922200 -3.95093500

C -6.28842400 -2.11630700 -2.37208000

H -7.20089700 -2.95898800 -0.59843200

H 0.60194500 4.61875700 4.17966000

C 1.79174800 4.65691100 2.37202600

H 2.74276300 4.51008500 0.43657500

H -3.94032200 6.35193300 -0.31670000

C -3.86637700 5.14527200 -2.11420600

H -3.60901900 3.74534200 -3.74348100

H -2.05640900 -4.96073700 4.25065300

H -7.10288600 -2.42282600 -3.03348200

H 2.58204900 5.27532200 2.80554700

H -4.41863100 5.87032700 -2.71778000

C 5.23034700 2.58290500 0.15442600

C 5.82323600 1.41157000 0.66181000

C 5.59344000 0.17364200 0.06829000

C 4.75189700 0.10039700 -1.04673000

C 4.15080600 1.24176200 -1.57652500

C 4.39752000 2.47652300 -0.96921700

H 6.48199400 1.47622700 1.53239900

H 6.06656800 -0.73149400 0.45394700

H 3.48358600 1.15851800 -2.43512600

H 3.92936000 3.37188800 -1.38631800

C 5.47738400 3.90897400 0.82923400

H 5.22996400 4.75494400 0.17064000

H 4.85975400 4.00206600 1.73924700

H 6.52933100 4.01288700 1.13808300

S 4.41493600 -1.50786200 -1.76923500

O 5.64628700 -2.30489500 -1.71519200

O 3.72589800 -1.26968700 -3.03989900

N 3.34365300 -2.20818600 -0.68413700

C 3.72378000 -3.38674300 0.08570600

H 4.79334500 -3.55728900 -0.10891400

H 3.61635700 -3.18457600 1.16535700

C 2.96196200 -4.62776500 -0.30025500

H 2.90000400 -4.81285500 -1.37911900

C 2.40944600 -5.48218600 0.56453300

H 2.45136100 -5.30701000 1.64525500

H 1.90111400 -6.39022400 0.22887400

C 2.01013900 -1.57775000 -0.57651900

H 2.07424800 -0.64137500 -1.14489200

C 1.70408400 -1.17999900 0.85969900

C 1.91815800 0.15558000 1.23943300

C 1.27862800 -2.09492200 1.83509000

C 1.70806600 0.57033900 2.55523200

H 2.23868400 0.87874500 0.48916900

C 1.06503900 -1.68105700 3.15344000

H 1.11521000 -3.13902000 1.56805700

C 1.27613400 -0.34813000 3.51762100

H 1.87334800 1.61612100 2.82168800

H 0.72821200 -2.40744700 3.89594700

H 1.10928700 -0.02640700 4.54946500

**TS18**

Rh -1.01681500 0.09444300 -0.61239500

Cl 0.56533300 1.15331900 -2.14473700

C 0.91587400 -2.28817400 -1.33799600

C 0.75687400 -2.18315300 -2.74003800

C -0.23374000 -3.13453700 -0.89049800

C -0.55387200 -1.82118500 -1.56216800

H 0.41263800 -3.03721700 -3.32855900

H 1.20574900 -1.33466600 -3.25971600

H -0.38419400 -4.08520900 -1.42207700

H -0.42707800 -3.17972600 0.17967700

H -1.26520800 -1.63105000 -2.37432000

P -2.77973700 -0.76079800 0.44143600

P -1.53210900 2.03290800 0.44753100

C -3.67583200 0.57973200 1.41400900

C -2.59845400 -2.13126600 1.67385900

C -4.15775400 -1.37100700 -0.63947500

C -2.68718400 1.65080300 1.86789400

C -0.24309000 3.10174400 1.22651600

C -2.49006000 3.27350200 -0.53573100

H -4.40699400 1.01028700 0.71159200

H -4.23893600 0.14047500 2.25183800

C -2.24819700 -1.84047000 3.00307100

C -2.69844500 -3.48053000 1.28934100

C -4.15194900 -1.02628800 -2.00068600

C -5.25723100 -2.08028100 -0.12389500

H -2.04908200 1.27866300 2.68452900

H -3.20386100 2.55615800 2.22225700

C -0.31409300 3.54883900 2.55664300

C 0.85695400 3.48467100 0.43891900

C -2.96701300 4.46370500 0.03956200

C -2.74957000 3.01280600 -1.88944700

H -2.13273900 -0.80521900 3.32841100

C -2.02260300 -2.86557500 3.92473700

C -2.45877100 -4.50646600 2.20719700

H -2.96932300 -3.73406300 0.26230700

H -3.31395100 -0.44610400 -2.39674900

C -5.21224200 -1.39760200 -2.83377000

C -6.31668700 -2.44971700 -0.95628900

H -5.28514500 -2.35322900 0.93382900

H -1.15204800 3.27188500 3.19776500

C 0.69602800 4.35534400 3.09237600

C 1.85638000 4.29823100 0.97453100

H 0.93640300 3.11887900 -0.58777500

H -2.74263700 4.69584400 1.08467200

C -3.71239400 5.36679300 -0.72090000

C -3.49179500 3.92171700 -2.65131500

H -2.34791100 2.10102200 -2.33813500

H -1.75670700 -2.61584400 4.95512300

C -2.12271300 -4.20304600 3.53032600

H -2.54046100 -5.54844900 1.88686100

H -5.19189900 -1.12331100 -3.89166900

C -6.29471200 -2.11281600 -2.31438700

H -7.16384400 -3.00330200 -0.54271200

H 0.62760900 4.68983000 4.13091900

C 1.78473600 4.73313200 2.30259900

H 2.70221100 4.59044600 0.35062100

H -4.08121200 6.28921200 -0.26439600

C -3.97840800 5.09483800 -2.06856300

H -3.68535400 3.71238400 -3.70657700

H -1.94070600 -5.00499600 4.25017900

H -7.12321900 -2.40498500 -2.96487000

H 2.57304400 5.36653200 2.71799300

H -4.55905200 5.80385000 -2.66471400

C 5.20441900 2.52668300 0.19101100

C 5.78697500 1.34232400 0.67985700

C 5.56207200 0.11883700 0.05540300

C 4.73664000 0.07552100 -1.07315000

C 4.14848100 1.23084100 -1.58806200

C 4.39120200 2.44980200 -0.94947700

H 6.43057700 1.38441400 1.56287600

H 6.02414000 -0.79674800 0.42964500

H 3.48765200 1.17087700 -2.45342100

H 3.92994100 3.35524100 -1.35128800

C 5.43151300 3.83475100 0.90556200

H 5.25310800 4.69653600 0.24520900

H 4.74474000 3.92727200 1.76448500

H 6.45813300 3.90791900 1.29657900

S 4.38550100 -1.51503100 -1.81979500

O 5.59982300 -2.33665200 -1.77512100

O 3.69689900 -1.25785600 -3.08796000

N 3.29215100 -2.22344600 -0.75118500

C 3.66538400 -3.40377200 0.02215100

H 4.72355700 -3.60349500 -0.20403500

H 3.59607200 -3.19166900 1.10255200

C 2.85798700 -4.62322000 -0.33798000

H 2.78289000 -4.82249100 -1.41378900

C 2.27858300 -5.44503500 0.54088800

H 2.33575100 -5.26076500 1.61914600

H 1.73465400 -6.33748600 0.21949300

C 2.00179400 -1.53000800 -0.58730700

H 2.08044800 -0.59019300 -1.14900100

C 1.73117500 -1.13063600 0.85390000

C 1.93993400 0.21194400 1.21150300

C 1.34540200 -2.03861800 1.85140900

C 1.76707300 0.63928000 2.52821100

H 2.21822100 0.93226300 0.44236500

C 1.16831900 -1.61203200 3.17103500

H 1.17579800 -3.08616300 1.60313900

C 1.37738900 -0.27333700 3.51375700

H 1.92262300 1.69119300 2.77565000

H 0.85859600 -2.33297400 3.93042900

H 1.23762200 0.05842600 4.54631900

**19**

Rh -1.15881700 0.10479000 -0.80475700

Cl 0.46561100 1.43891600 -2.04541600

C 1.42385300 -2.84956100 -1.35161800

C 2.02248300 -3.79597200 -2.07994300

C -0.09609100 -2.72393000 -1.28895700

C -0.53295600 -1.37652600 -1.76932000

H 1.44196100 -4.52980900 -2.64524800

H 3.10887900 -3.84727100 -2.15139900

H -0.53530600 -3.48847900 -1.95754800

H -0.44584900 -2.91532400 -0.26647100

H -0.23923300 -1.20913500 -2.82374900

P -2.92279600 -0.90879300 0.19081200

P -1.80126100 2.07539400 0.51136300

C -3.94795800 0.39697500 1.07352000

C -2.68957700 -2.22490000 1.46588300

C -4.18800300 -1.67278000 -0.91623500

C -3.08112600 1.47607600 1.73052900

C -0.57545800 2.97944600 1.53877000

C -2.66938600 3.40743600 -0.41614700

H -4.57609300 0.84230900 0.28515400

H -4.62397900 -0.08490800 1.79737200

C -2.59777700 -1.92412900 2.83442800

C -2.54601000 -3.56496700 1.06188300

C -4.07308600 -1.52486200 -2.30660700

C -5.30085100 -2.35161000 -0.38782800

H -2.51649000 1.06590500 2.58170100

H -3.70222500 2.30390500 2.10701500

C -0.80500100 3.33729900 2.87772100

C 0.66394900 3.28620800 0.94894500

C -3.25598200 4.50264500 0.24124200

C -2.73922000 3.32189000 -1.81546800

H -2.69118200 -0.89590300 3.18502800

C -2.38014800 -2.93502500 3.77414900

C -2.31254300 -4.57247500 2.00016900

H -2.63763100 -3.82790800 0.00602100

H -3.20913100 -0.99456800 -2.71588400

C -5.05323800 -2.04966500 -3.15612500

C -6.27895200 -2.87215000 -1.23726800

H -5.39789900 -2.48601900 0.69271600

H -1.75766100 3.11451800 3.36222100

C 0.19210500 3.98035300 3.61881500

C 1.65396100 3.93054100 1.69276800

H 0.85650900 2.98636100 -0.08453300

H -3.18019800 4.59581500 1.32850800

C -3.92160300 5.48833600 -0.48912900

C -3.40551500 4.31374300 -2.54403200

H -2.25216500 2.48863900 -2.32761800

H -2.31642600 -2.67974300 4.83496000

C -2.23140300 -4.26138400 3.36138100

H -2.20347200 -5.60731800 1.66590000

H -4.95151100 -1.93139900 -4.23783900

C -6.15598000 -2.72267000 -2.62389100

H -7.13902100 -3.39973400 -0.81678400

H 0.00369300 4.24836100 4.66168700

C 1.42441400 4.27560400 3.02907900

H 2.61740400 4.14561100 1.22865000

H -4.37513400 6.33755400 0.02869800

C -3.99954200 5.39252300 -1.88429700

H -3.45335400 4.24282000 -3.63358100

H -2.05493200 -5.05054900 4.09644700

H -6.92059100 -3.13388500 -3.28806000

H 2.20583500 4.77141800 3.61070800

H -4.51852900 6.16632300 -2.45609700

C 5.11879300 3.05933300 -0.11606500

C 6.03441800 2.03812500 0.19881100

C 5.89434200 0.75559200 -0.32767000

C 4.81724700 0.48579200 -1.18040600

C 3.90223200 1.48119200 -1.52509600

C 4.05789500 2.75994600 -0.98385800

H 6.87777900 2.25735300 0.86010200

H 6.61981400 -0.02832000 -0.10351300

H 3.07269300 1.26204800 -2.19803900

H 3.33600000 3.53507800 -1.25473300

C 5.28747600 4.43672000 0.47644000

H 4.59650400 5.16328000 0.02418200

H 5.10005500 4.42344300 1.56408700

H 6.31446200 4.81018300 0.33389400

S 4.56992200 -1.18073000 -1.79648600

O 5.87758900 -1.84596300 -1.81535800

O 3.73179800 -1.09380200 -2.99216700

N 3.65665700 -1.96131700 -0.61710700

C 4.35679700 -2.55958900 0.52312300

H 5.42334800 -2.31965900 0.40412200

H 4.02076700 -2.09550300 1.46424100

C 4.23240100 -4.05868400 0.59452100

H 4.51655800 -4.59025500 -0.32025100

C 3.85646900 -4.73785200 1.68054200

H 3.55933200 -4.22144600 2.59941000

H 3.82886700 -5.83111500 1.69174300

C 2.19624000 -1.74467100 -0.62680300

H 2.01477900 -0.83665100 -1.22669500

C 1.68662500 -1.42588600 0.77508500

C 1.69252900 -0.09525300 1.21863200

C 1.27082600 -2.43189700 1.65774500

C 1.29939600 0.22234500 2.52194700

H 1.99336200 0.69676900 0.53098800

C 0.86898600 -2.11730400 2.95772900

H 1.27409100 -3.47194200 1.32647300

C 0.88402500 -0.78885300 3.39359900

H 1.30994700 1.26288900 2.85046000

H 0.53748500 -2.91304800 3.62760200

H 0.56928200 -0.54094900 4.41104900

**20**

C 1.75777500 -0.94164800 -0.65247100

C 0.53042100 -0.34276200 -1.31419900

C 2.98398200 -0.11566200 1.50087900

C -0.29535500 0.60140600 1.62546600

C 0.61881600 -0.05144100 2.62672600

H 1.47415600 -1.73749600 0.04868900

H 3.91188300 0.46557900 1.60252000

H 2.36317300 -1.42030700 -1.44420200

H 0.24153000 0.18867200 3.63932800

H 0.62409200 -1.14264600 2.51981000

N 2.58819700 0.03012800 0.07817500

S 3.46394800 1.15512400 -0.80491500

O 3.49759900 2.41079100 -0.04192800

O 2.95418000 1.11562900 -2.18050500

C 5.14582700 0.53001400 -0.86193700

C 6.08168000 0.93219300 0.09374200

C 5.48197200 -0.40406300 -1.84846100

C 7.35461900 0.35890300 0.08232900

H 5.81443200 1.69954300 0.82209500

C 6.75836300 -0.96429700 -1.84557300

H 4.74853800 -0.67245200 -2.61079000

C 7.71009600 -0.60496400 -0.87459300

H 8.08838500 0.67095000 0.83042300

H 7.02513200 -1.69292000 -2.61614400

C 9.07011600 -1.25542700 -0.85256400

H 9.41927500 -1.49901600 -1.86773400

H 9.82214100 -0.61039800 -0.37385000

H 9.03683700 -2.20049900 -0.28273700

C 3.38576000 -1.54777300 1.86620600

C 3.17410000 -2.05332800 3.15675500

C 4.07518600 -2.35011100 0.94326400

C 3.62158300 -3.33005100 3.50945900

H 2.66086400 -1.44268000 3.90091100

C 4.52343500 -3.62498400 1.29264900

H 4.27122700 -1.97390300 -0.05989300

C 4.29471100 -4.12410900 2.57842800

H 3.43712600 -3.70530000 4.51936800

H 5.05417300 -4.23088500 0.55350900

H 4.63856500 -5.12486000 2.85205800

C 2.01031300 0.53803400 2.47716800

C 2.39072200 1.63516300 3.14554100

H 1.71826500 2.13086000 3.85255700

H 3.37262100 2.09073300 2.99130500

C -0.47061400 -1.10861100 -1.90276000

H 0.69254000 0.65744200 -1.71304000

H -1.03057600 -0.69977900 -2.74808500

H -0.51039700 -2.19205800 -1.76807800

H -0.27078600 1.69114700 1.79870100

Rh -1.17225800 -0.19017500 0.14624800

P -1.95224400 1.87994000 -0.39392800

P -3.27767700 -0.98347400 -0.70581000

C -3.69110900 1.72836500 -1.06880300

C -0.95614200 2.79087400 -1.63422400

C -2.23047600 3.11861800 0.94840600

C -3.91562000 0.39829800 -1.78723000

C -3.52078200 -2.50091900 -1.71149100

C -4.58510600 -1.14440700 0.58736300

H -4.33753000 1.78877400 -0.17914200

H -3.93536600 2.59855200 -1.69699500

C 0.37113000 3.09785800 -1.27883700

C -1.41272200 3.11669200 -2.91998400

C -2.82091600 2.66532100 2.14187500

C -1.93290400 4.48165900 0.80614600

H -3.33930100 0.36040900 -2.72521100

H -4.97860100 0.25590800 -2.03726600

C -3.93669400 -2.47969000 -3.05325900

C -3.22821300 -3.73789800 -1.10640600

C -5.93311900 -1.35485200 0.25047000

C -4.21858400 -1.05224500 1.93850700

H 0.76535500 2.81164600 -0.30038000

C 1.21835800 3.73396100 -2.18461200

C -0.55908200 3.75360400 -3.82810800

H -2.43215900 2.87558500 -3.22903100

H -3.02992700 1.59998800 2.26579400

C -3.11520200 3.56227600 3.16931200

C -2.22148100 5.37709300 1.84244800

H -1.46961800 4.85056000 -0.11076300

H -4.16957300 -1.53852700 -3.55423400

C -4.06341700 -3.67165100 -3.77510200

C -3.35916600 -4.92351200 -1.83094100

H -2.87020300 -3.76227800 -0.07537900

H -6.22577300 -1.46430000 -0.79772800

C -6.90425600 -1.44236800 1.24998700

C -5.19259000 -1.14902500 2.93835200

H -3.16317800 -0.92255100 2.19372200

H 2.25114500 3.92834600 -1.89176400

C 0.75249700 4.06655800 -3.46120600

H -0.92273500 4.00072900 -4.82892700

H -3.57374700 3.19705900 4.09179600

C -2.81317900 4.92141300 3.02291600

H -1.97962700 6.43633400 1.72309100

H -4.38942700 -3.63934100 -4.81810400

C -3.77694200 -4.89565800 -3.16630000

H -3.12539100 -5.87637900 -1.34917800

H -7.95156300 -1.60109000 0.97969400

C -6.53462900 -1.33614600 2.59650800

H -4.89781200 -1.08295000 3.98887400

H 1.41883900 4.55457000 -4.17688500

H -3.03554900 5.62285600 3.83121200

H -3.87609300 -5.82612000 -3.73137000

H -7.29492400 -1.40912400 3.37868400

Cl -0.80786700 -2.48270400 1.06103000

**TS21**

C 2.14302900 -0.37737200 0.00369200

C 0.93156700 0.18218500 -0.70472200

C 3.19904400 0.81824000 2.02571800

C -0.13769600 1.15671200 1.27308800

C 0.65743900 0.87733700 2.51887400

H 1.83097600 -1.06697500 0.80349600

H 4.06822200 1.47896700 2.14287000

H 2.70854000 -1.00372400 -0.71212500

H 0.01008200 1.26911800 3.32930700

H 0.72809000 -0.20432400 2.70039600

N 3.04298400 0.62891800 0.57111100

S 3.97671500 1.54392900 -0.47958800

O 4.71320600 2.52017800 0.32929000

O 3.13399600 1.97849100 -1.60202500

C 5.16030100 0.37758100 -1.15357500

C 6.34961600 0.13428500 -0.45675700

C 4.86407700 -0.30804600 -2.33347300

C 7.23659800 -0.82197100 -0.94556000

H 6.56897200 0.69573700 0.45301100

C 5.76483900 -1.26633700 -2.80656300

H 3.94465100 -0.07845100 -2.87467500

C 6.95874300 -1.54136100 -2.12293600

H 8.16719900 -1.01552100 -0.40463400

H 5.53484400 -1.80733400 -3.72837200

C 7.94038100 -2.56443600 -2.63664900

H 7.52664100 -3.14319200 -3.47531100

H 8.86617700 -2.07774900 -2.98795000

H 8.23041400 -3.27130900 -1.84246700

C 3.54257600 -0.48915600 2.74226900

C 3.17877700 -0.69112800 4.08116900

C 4.29472400 -1.48229100 2.09652700

C 3.55114800 -1.85737200 4.75559700

H 2.59830500 0.07280100 4.60233500

C 4.66847500 -2.64818800 2.76862100

H 4.58961900 -1.34467100 1.05523700

C 4.29609600 -2.84234800 4.10202100

H 3.25053100 -1.99803700 5.79706000

H 5.25090100 -3.41036000 2.24412700

H 4.58109400 -3.75756800 4.62708300

C 2.00357000 1.56119200 2.60505000

C 2.16023600 2.78652900 3.12322200

H 1.31176700 3.34061000 3.53752500

H 3.13455200 3.28355800 3.13495100

C 0.00631800 -0.65747100 -1.34862300

H 1.04451100 1.19683500 -1.08017600

H -0.46909000 -0.29779100 -2.26599100

H 0.13805800 -1.74210100 -1.28079300

H 0.02381900 2.20049000 0.95510900

Rh -1.33250300 -0.06530000 0.29468900

P -2.40993500 1.69481100 -0.62347000

P -3.10469700 -1.36637100 -0.52815400

C -4.11196900 1.15812900 -1.17543600

C -1.57393600 2.36434700 -2.12242800

C -2.78007000 3.20198700 0.37058100

C -4.10220400 -0.28394700 -1.68718200

C -2.84601000 -2.90414400 -1.51470700

C -4.34905100 -1.88092600 0.73375300

H -4.73410200 1.23944600 -0.26974900

H -4.51991600 1.86575500 -1.91515900

C -0.36405700 3.06195200 -1.94522900

C -1.99706600 2.07185200 -3.42922000

C -2.86655500 3.09121900 1.76757500

C -3.03596000 4.44230100 -0.23789200

H -3.60634900 -0.34979200 -2.66813500

H -5.12721100 -0.66894000 -1.80220300

C -3.57227400 -3.21087100 -2.67885900

C -1.86073800 -3.80490100 -1.06894500

C -5.50192000 -2.59889500 0.37304400

C -4.14646800 -1.53147600 2.07705900

H -0.01414000 3.30545100 -0.93969400

C 0.41598400 3.43308500 -3.04039600

C -1.22302100 2.45966800 -4.52893600

H -2.93179300 1.53693400 -3.60631500

H -2.65086900 2.12863600 2.23828000

C -3.20722900 4.20257700 2.54344800

C -3.36986500 5.55344100 0.54124200

H -2.96240000 4.54541300 -1.32328200

H -4.34621200 -2.53668200 -3.05096300

C -3.31977600 -4.39282700 -3.38315800

C -1.61581800 -4.98552400 -1.77449000

H -1.28631000 -3.56882700 -0.16833800

H -5.65558800 -2.90503800 -0.66554700

C -6.44966100 -2.94320300 1.33891300

C -5.09582200 -1.88179100 3.04294300

H -3.23233000 -1.00279500 2.35596600

H 1.37319600 3.93052500 -2.87213300

C -0.01292600 3.13143700 -4.33766600

H -1.56692400 2.22571800 -5.53985700

H -3.26997500 4.10608400 3.63036700

C -3.45642700 5.43557300 1.93237300

H -3.55942800 6.51620800 0.05960400

H -3.89284300 -4.61724500 -4.28681400

C -2.34171800 -5.28334400 -2.93259000

H -0.84801500 -5.67608600 -1.41578900

H -7.34410900 -3.50120500 1.04952500

C -6.24835100 -2.58198900 2.67652600

H -4.92745900 -1.61176300 4.08867000

H 0.59894400 3.41582300 -5.19729500

H -3.71349200 6.30686000 2.54034100

H -2.14551500 -6.20698100 -3.48348100

H -6.98822800 -2.85546500 3.43353300

Cl -0.57254900 -2.00268700 1.65796800

**22**

C 2.14302900 -0.37737200 0.00369200

C 0.93156700 0.18218500 -0.70472200

C 3.19904400 0.81824000 2.02571800

C -0.13769600 1.15671200 1.27308800

C 0.65743900 0.87733700 2.51887400

H 1.83097600 -1.06697500 0.80349600

H 4.06822200 1.47896700 2.14287000

H 2.70854000 -1.00372400 -0.71212500

H 0.01008200 1.26911800 3.32930700

H 0.72809000 -0.20432400 2.70039600

N 3.04298400 0.62891800 0.57111100

S 3.97671500 1.54392900 -0.47958800

O 4.71320600 2.52017800 0.32929000

O 3.13399600 1.97849100 -1.60202500

C 5.16030100 0.37758100 -1.15357500

C 6.34961600 0.13428500 -0.45675700

C 4.86407700 -0.30804600 -2.33347300

C 7.23659800 -0.82197100 -0.94556000

H 6.56897200 0.69573700 0.45301100

C 5.76483900 -1.26633700 -2.80656300

H 3.94465100 -0.07845100 -2.87467500

C 6.95874300 -1.54136100 -2.12293600

H 8.16719900 -1.01552100 -0.40463400

H 5.53484400 -1.80733400 -3.72837200

C 7.94038100 -2.56443600 -2.63664900

H 7.52664100 -3.14319200 -3.47531100

H 8.86617700 -2.07774900 -2.98795000

H 8.23041400 -3.27130900 -1.84246700

C 3.54257600 -0.48915600 2.74226900

C 3.17877700 -0.69112800 4.08116900

C 4.29472400 -1.48229100 2.09652700

C 3.55114800 -1.85737200 4.75559700

H 2.59830500 0.07280100 4.60233500

C 4.66847500 -2.64818800 2.76862100

H 4.58961900 -1.34467100 1.05523700

C 4.29609600 -2.84234800 4.10202100

H 3.25053100 -1.99803700 5.79706000

H 5.25090100 -3.41036000 2.24412700

H 4.58109400 -3.75756800 4.62708300

C 2.00357000 1.56119200 2.60505000

C 2.16023600 2.78652900 3.12322200

H 1.31176700 3.34061000 3.53752500

H 3.13455200 3.28355800 3.13495100

C 0.00631800 -0.65747100 -1.34862300

H 1.04451100 1.19683500 -1.08017600

H -0.46909000 -0.29779100 -2.26599100

H 0.13805800 -1.74210100 -1.28079300

H 0.02381900 2.20049000 0.95510900

Rh -1.33250300 -0.06530000 0.29468900

P -2.40993500 1.69481100 -0.62347000

P -3.10469700 -1.36637100 -0.52815400

C -4.11196900 1.15812900 -1.17543600

C -1.57393600 2.36434700 -2.12242800

C -2.78007000 3.20198700 0.37058100

C -4.10220400 -0.28394700 -1.68718200

C -2.84601000 -2.90414400 -1.51470700

C -4.34905100 -1.88092600 0.73375300

H -4.73410200 1.23944600 -0.26974900

H -4.51991600 1.86575500 -1.91515900

C -0.36405700 3.06195200 -1.94522900

C -1.99706600 2.07185200 -3.42922000

C -2.86655500 3.09121900 1.76757500

C -3.03596000 4.44230100 -0.23789200

H -3.60634900 -0.34979200 -2.66813500

H -5.12721100 -0.66894000 -1.80220300

C -3.57227400 -3.21087100 -2.67885900

C -1.86073800 -3.80490100 -1.06894500

C -5.50192000 -2.59889500 0.37304400

C -4.14646800 -1.53147600 2.07705900

H -0.01414000 3.30545100 -0.93969400

C 0.41598400 3.43308500 -3.04039600

C -1.22302100 2.45966800 -4.52893600

H -2.93179300 1.53693400 -3.60631500

H -2.65086900 2.12863600 2.23828000

C -3.20722900 4.20257700 2.54344800

C -3.36986500 5.55344100 0.54124200

H -2.96240000 4.54541300 -1.32328200

H -4.34621200 -2.53668200 -3.05096300

C -3.31977600 -4.39282700 -3.38315800

C -1.61581800 -4.98552400 -1.77449000

H -1.28631000 -3.56882700 -0.16833800

H -5.65558800 -2.90503800 -0.66554700

C -6.44966100 -2.94320300 1.33891300

C -5.09582200 -1.88179100 3.04294300

H -3.23233000 -1.00279500 2.35596600

H 1.37319600 3.93052500 -2.87213300

C -0.01292600 3.13143700 -4.33766600

H -1.56692400 2.22571800 -5.53985700

H -3.26997500 4.10608400 3.63036700

C -3.45642700 5.43557300 1.93237300

H -3.55942800 6.51620800 0.05960400

H -3.89284300 -4.61724500 -4.28681400

C -2.34171800 -5.28334400 -2.93259000

H -0.84801500 -5.67608600 -1.41578900

H -7.34410900 -3.50120500 1.04952500

C -6.24835100 -2.58198900 2.67652600

H -4.92745900 -1.61176300 4.08867000

H 0.59894400 3.41582300 -5.19729500

H -3.71349200 6.30686000 2.54034100

H -2.14551500 -6.20698100 -3.48348100

H -6.98822800 -2.85546500 3.43353300

Cl -0.57254900 -2.00268700 1.65796800

**TS23**

C -2.84859900 0.85229300 -0.70545700

C -1.57887200 0.01647300 -0.68814400

C -3.11436000 1.89597900 1.59012300

C -0.48858400 0.36771400 0.28676000

C -0.57641700 1.70483200 1.01521200

H -2.62994000 1.89491100 -0.97656400

H -3.77630100 1.74676700 2.45485100

H -3.53995300 0.47410300 -1.47636800

H 0.38264200 1.92242800 1.50301900

H -0.73498000 2.52295400 0.29963500

N -3.55830800 0.90770100 0.58172200

S -4.23989600 -0.52172600 1.16865000

O -4.92526800 -0.17354300 2.41822900

O -3.28588800 -1.64230900 1.15142800

C -5.46813900 -0.85816100 -0.09167200

C -6.66781100 -0.13850100 -0.06936300

C -5.21324000 -1.81159800 -1.07701000

C -7.61680300 -0.37943900 -1.05938200

H -6.84659600 0.58946000 0.72443800

C -6.17679800 -2.03729300 -2.06470900

H -4.27205200 -2.36408100 -1.06856800

C -7.38768200 -1.32929300 -2.07351000

H -8.55826700 0.17701200 -1.04794800

H -5.98080800 -2.77973600 -2.84294400

C -8.43614300 -1.57929800 -3.12808500

H -8.76125200 -0.63634700 -3.59689500

H -8.06802500 -2.24633400 -3.92116800

H -9.33344700 -2.04461800 -2.68590200

C -3.38022900 3.31462200 1.08180500

C -2.56124200 4.38556600 1.46564500

C -4.49803800 3.57865900 0.27464400

C -2.84874300 5.68910400 1.04870600

H -1.69109600 4.19995100 2.09853500

C -4.78702500 4.88001400 -0.14160800

H -5.13847400 2.74851400 -0.03113900

C -3.96145400 5.94182900 0.24263300

H -2.19393700 6.50958600 1.35320600

H -5.66112100 5.06579000 -0.77158800

H -4.18317400 6.95998200 -0.08726800

C -1.68388600 1.64696000 2.05163100

C -1.43753800 1.31926000 3.32716700

H -0.41998300 1.10978000 3.67117000

H -2.24238700 1.22889600 4.06243300

C -0.45045200 0.32408300 -1.61114900

H -1.81513400 -1.04829600 -0.65348700

H -0.14843400 -0.45299100 -2.32314300

H -0.41798300 1.34658700 -1.99890200

H -0.27489000 -0.45338100 0.97473800

Rh 1.58910700 0.24706600 -0.69418800

P 1.66248800 -1.92421900 -0.12521100

P 3.86118300 0.00989000 -0.86145600

C 3.30030900 -2.73938600 -0.61941300

C 0.33967800 -3.03685200 -0.76067100

C 1.66270900 -2.22805100 1.70190100

C 4.20604500 -1.75422100 -1.36864500

C 4.89572200 0.96781300 -2.03314800

C 4.65819800 0.24281100 0.77912200

H 3.76921600 -3.07429100 0.31686300

H 3.09492700 -3.64010300 -1.21645800

C -0.82157300 -3.28709900 -0.01008700

C 0.39808600 -3.48905700 -2.09176100

C 1.87631900 -1.14336000 2.56701800

C 1.55704600 -3.52212500 2.24140400

H 3.97744700 -1.75354600 -2.44604100

H 5.27388000 -2.00593900 -1.26793700

C 6.28822700 1.05305200 -1.86942100

C 4.28697200 1.57758800 -3.14095500

C 5.45839100 -0.72145900 1.40858700

C 4.34720600 1.43471300 1.46271900

H -0.92247700 -2.90948400 1.00839600

C -1.89224300 -3.98888000 -0.57218200

C -0.66865900 -4.19757300 -2.64871000

H 1.27619700 -3.27382700 -2.70762200

H 1.97067300 -0.13777200 2.15129000

C 1.98095300 -1.34696200 3.94620600

C 1.65560200 -3.72296500 3.61995300

H 1.38752600 -4.37790800 1.58260500

H 6.76654400 0.59919400 -0.99724200

C 7.06561300 1.72979200 -2.81247100

C 5.07007300 2.25069600 -4.08308700

H 3.20026500 1.55010700 -3.23855600

H 5.70901100 -1.65573800 0.90173000

C 5.93667200 -0.50491000 2.70626100

C 4.83796000 1.65128800 2.75055600

H 3.69849000 2.17396000 0.98189000

H -2.78953400 -4.14771400 0.02942100

C -1.81708400 -4.45149700 -1.88858900

H -0.60650800 -4.54675100 -3.68268300

H 2.15501300 -0.49275500 4.60505800

C 1.86806700 -2.63510500 4.47515600

H 1.56452900 -4.73235000 4.02954700

H 8.14846900 1.79593800 -2.67809300

C 6.45690000 2.32686800 -3.92194300

H 4.59052000 2.73069600 -4.93991900

H 6.54926000 -1.26870800 3.19213800

C 5.62827500 0.67966000 3.37852400

H 4.59258600 2.57956600 3.27303100

H -2.65338200 -5.00230600 -2.32712700

H 1.94512500 -2.79461300 5.55378200

H 7.06506000 2.86086800 -4.65694400

H 5.99971600 0.84603800 4.39300700

Cl 1.77961300 2.63250100 -1.06781600

**24**

C -3.10011300 0.50295500 -0.98240500

C -1.75507000 -0.19137000 -0.93362600

C -3.46709800 1.54467000 1.31703200

C -0.69291600 0.38904600 -0.04269600

C -0.94317400 1.68975800 0.70912700

H -3.00089200 1.55412200 -1.28933300

H -4.08531000 1.30726700 2.19357200

H -3.75624600 0.02853200 -1.73053300

H 0.00145200 2.02434500 1.16191300

H -1.23761600 2.48432600 0.00719000

N -3.79207300 0.51016700 0.31487500

S -4.52920400 -0.91384100 0.80744000

O -4.91965300 -0.73813300 2.20984100

O -3.72103800 -2.06971500 0.39032300

C -6.02061100 -0.94249000 -0.18847600

C -7.11373100 -0.16329800 0.20899400

C -6.06817200 -1.70999700 -1.35224500

C -8.25702900 -0.14786900 -0.58562000

H -7.06206900 0.41274300 1.13473600

C -7.22420500 -1.68049400 -2.13843400

H -5.21108900 -2.32713700 -1.62676800

C -8.33148200 -0.90137400 -1.77285000

H -9.11464600 0.45788500 -0.27944200

H -7.26487200 -2.27920500 -3.05233800

C -9.58555700 -0.87503700 -2.60990700

H -9.88661900 0.15945100 -2.84231000

H -9.45514200 -1.41430000 -3.55942700

H -10.42733400 -1.34121600 -2.07027500

C -3.91439100 2.92421100 0.82295900

C -3.27722700 4.08987100 1.27269000

C -5.01816600 3.05168600 -0.03467400

C -3.72926400 5.35113300 0.87303600

H -2.42027300 4.00946900 1.94488800

C -5.47173900 4.31132700 -0.43413700

H -5.52254100 2.15161500 -0.39204700

C -4.82765600 5.46762100 0.01689100

H -3.21560500 6.24711100 1.23111700

H -6.33248400 4.38860000 -1.10386100

H -5.17814200 6.45344600 -0.29917000

C -2.00894700 1.48852500 1.76586500

C -1.70814600 1.20458900 3.03933300

H -0.66912800 1.14007600 3.37490600

H -2.48675300 1.00102100 3.78026500

C -0.51888400 0.37783600 -1.56791900

H -1.83118500 -1.27802800 -0.91988200

H 0.16944900 -0.32935200 -2.05105800

H -0.55688000 1.35743800 -2.05012100

H -0.17120900 -0.34821700 0.57001200

Rh 2.05951300 0.21951200 -0.69070500

P 2.06257700 -1.82623700 0.21928800

P 4.23263400 0.02181500 -0.68273900

C 3.78555500 -2.59874100 0.16118100

C 0.91503400 -3.08335300 -0.48509300

C 1.70056400 -1.85492500 2.03340500

C 4.68090300 -1.79033200 -0.78059200

C 5.23715000 0.75900400 -2.03215200

C 5.01050800 0.64734700 0.86700700

H 4.16645200 -2.55943800 1.19256800

H 3.72038400 -3.65935200 -0.12416300

C -0.39692500 -3.21944800 0.00225200

C 1.28868700 -3.80270600 -1.63440400

C 1.80006300 -0.64857700 2.74620600

C 1.41154000 -3.04099000 2.73007400

H 4.49057400 -2.05089900 -1.83354400

H 5.75421800 -1.95422300 -0.59521700

C 6.62592800 0.91484700 -1.88854900

C 4.61950800 1.11574400 -3.24038500

C 5.90524100 -0.09897400 1.64904100

C 4.62528000 1.93125800 1.29838500

H -0.72753200 -2.66077300 0.88000500

C -1.31310600 -4.05723500 -0.64069500

C 0.37770500 -4.65150000 -2.26708100

H 2.29355200 -3.69184000 -2.05126900

H 2.03109400 0.27302600 2.20406800

C 1.60861900 -0.62795200 4.13092500

C 1.21512200 -3.01640500 4.11290800

H 1.32755200 -3.98720800 2.18968100

H 7.11241200 0.66105900 -0.94296100

C 7.39008300 1.40987000 -2.94811800

C 5.38888400 1.61012900 -4.29744600

H 3.53533300 1.03003900 -3.33349400

H 6.21905800 -1.09813800 1.33981100

C 6.40665800 0.42586700 2.84610400

C 5.13819300 2.45448700 2.48595400

H 3.90976200 2.50519300 0.70126000

H -2.33335500 -4.11365600 -0.25565300

C -0.92582900 -4.77922800 -1.77300800

H 0.68448900 -5.20847300 -3.15627900

H 1.69057100 0.31690200 4.67414300

C 1.31215000 -1.80976700 4.81550900

H 0.98232900 -3.94264000 4.64462600

H 8.46981200 1.53215600 -2.82888700

C 6.77209700 1.75613600 -4.15467700

H 4.90126300 1.89421000 -5.23342100

H 7.09654200 -0.16844200 3.45093900

C 6.02594200 1.70234900 3.26559800

H 4.83570300 3.45364100 2.81012500

H -1.63998500 -5.43599200 -2.27640900

H 1.15529700 -1.79287400 5.89712900

H 7.36972400 2.14919500 -4.98144900

H 6.41706600 2.11077500 4.20107100

Cl 2.05011800 2.50999100 -1.34749600

**TS18***

C 6.56171800 -1.01571100 -1.44408700

C 5.62184700 -0.02245400 -1.76788500

C 4.25794500 -0.22534500 -1.56529100

C 3.82404000 -1.43682100 -1.01936800

C 4.73088400 -2.45156400 -0.70694900

C 6.09246200 -2.23316100 -0.92406600

H 5.96415600 0.92846700 -2.18559600

H 3.53352600 0.54554500 -1.82889800

H 4.36377800 -3.39375700 -0.29873500

H 6.80590500 -3.02659400 -0.68473000

C 8.03688800 -0.76388900 -1.63049900

H 8.23673800 -0.20293700 -2.55692900

H 8.43625200 -0.16288900 -0.79484600

H 8.61009400 -1.70230300 -1.66606200

S 2.08229700 -1.66501200 -0.65348500

O 1.82773600 -3.10399400 -0.54816800

O 1.35206900 -0.82781800 -1.61587200

N 1.87209000 -1.02908400 0.88320100

C 1.83354200 -1.96369400 2.01963300

H 1.60770100 -1.34001000 2.89502100

H 0.97204800 -2.63692900 1.91768900

C 3.10903600 -2.72764200 2.25962500

H 4.03274600 -2.14022300 2.31164900

C 3.15835300 -4.05283200 2.41179400

H 2.25628200 -4.66659900 2.32500000

H 4.09944700 -4.57110300 2.61780200

C 1.77187500 0.42507500 1.02163100

H 1.60193800 0.78877800 -0.00238200

C 3.07564800 1.09127700 1.46240400

C 3.83464500 0.67101000 2.56752000

C 3.54368300 2.18573300 0.71900000

C 5.02867800 1.31384400 2.90455000

H 3.50032300 -0.16665100 3.17909600

C 4.73350600 2.83675100 1.05643600

H 2.97294800 2.52759000 -0.14570800

C 5.48520500 2.39944200 2.14929100

H 5.60573400 0.96341400 3.76425100

H 5.07399900 3.68343500 0.45464400

H 6.42004800 2.89986600 2.41405500

C 0.43318800 0.91132700 1.63579200

C 0.37416500 2.35803100 2.09852300

C -0.20935400 0.53360600 3.55222700

H -0.61728200 2.82187200 2.04437700

H 1.18152200 3.02256200 1.77474000

H -1.27423400 0.63266800 3.34543500

H 0.07189600 -0.35278000 4.12465500

H 1.72336700 1.48399500 3.64353900

Cl -1.55030900 -1.88517500 2.16269800

C -2.00064800 0.88818800 -2.48269300

C 0.12439400 2.61341000 -1.46557800

C -2.58564700 2.94424600 -0.56164000

C -3.26696200 0.10394800 -2.12822600

C -2.55491900 -2.59785000 -1.34106200

C -4.54293400 -1.16479400 0.21053300

H -2.19086600 1.67221400 -3.23244000

H -1.22184300 0.21311600 -2.86998700

C 1.01342500 2.09489300 -2.42511400

C 0.40000100 3.87733700 -0.91200100

C -2.85811500 3.96941500 -1.48470700

C -3.33812000 2.87777700 0.62124300

H -3.60286700 -0.53347100 -2.95905800

H -4.08643100 0.79737800 -1.88823600

C -1.30634200 -3.18683400 -1.10883400

C -3.48389000 -3.25283200 -2.16959500

C -5.45021900 -0.09727400 0.32135900

C -4.83309200 -2.37384700 0.86591000

H 0.85189300 1.10192900 -2.84272700

C 2.13147300 2.83059900 -2.83129500

C 1.52169200 4.60836200 -1.31390500

H -0.27628300 4.30402700 -0.16854100

H -2.27498200 4.04312700 -2.40684100

C -3.86377400 4.90447400 -1.23003600

C -4.34503900 3.81551000 0.87791900

H -3.13298400 2.06379400 1.32426700

H -0.59903400 -2.70023500 -0.44321300

C -0.97291700 -4.40167300 -1.71487700

C -3.15570200 -4.47260300 -2.76447700

H -4.47148700 -2.81505100 -2.34204700

H -5.23242600 0.86760900 -0.14154400

C -6.63732200 -0.24567400 1.04333300

C -6.01937700 -2.51796800 1.58925500

H -4.11896300 -3.19748000 0.82156800

H 2.80428700 2.41287700 -3.58497300

C 2.38952200 4.09025300 -2.28062300

H 1.71460600 5.58940600 -0.87210100

H -4.06804800 5.69568800 -1.95637300

C -4.61016000 4.82862000 -0.04722900

H -4.92644800 3.74725900 1.80105800

H 0.01845500 -4.82196300 -1.53260500

C -1.89582600 -5.04537300 -2.54251500

H -3.88338300 -4.97864700 -3.40481200

H -7.33548900 0.59285200 1.11375300

C -6.92744700 -1.45816800 1.67674700

H -6.23127200 -3.46443000 2.09350500

H 3.26333200 4.66362300 -2.60046800

H -5.39821600 5.56023700 0.14983300

H -1.63910800 -5.99674900 -3.01676100

H -7.85559800 -1.57433800 2.24260900

C 0.69461700 1.54392800 3.28829700

P -2.91006600 -0.94756700 -0.60908100

P -1.32920200 1.61373300 -0.89296900

Rh -1.26109000 0.00269400 0.63279600

**TS18-model**

Rh -0.85145200 0.03572200 -0.43284800

Cl 0.81943000 1.00489300 -1.92996100

C 1.05477400 -2.41258900 -0.99027300

C 0.95880200 -2.35398600 -2.40073000

C -0.13241900 -3.21734900 -0.56467800

C -0.39269400 -1.92202800 -1.29469000

H 0.62047100 -3.22072600 -2.97394400

H 1.44925500 -1.53444000 -2.92920400

H -0.28189900 -4.18280200 -1.06920300

H -0.37267500 -3.22029900 0.49690800

H -1.06377100 -1.74543700 -2.14334400

P -2.67754200 -0.74305200 0.57153800

P -1.36671100 2.02133800 0.53589000

C -3.58328700 0.65032000 1.45700200

C -2.58143300 -2.07251200 1.85731100

C -4.02153400 -1.36116900 -0.54706500

C -2.59047100 1.71509600 1.91656400

C -0.08794000 3.08889800 1.33329900

C -2.25231000 3.24676000 -0.53069400

H -4.27330300 1.07144900 0.70874100

H -4.19202100 0.25342800 2.28399000

C -2.28209200 -1.74238400 3.18977400

C -2.69611500 -3.43208700 1.51559400

C -3.94908400 -1.06520600 -1.91780100

C -5.15839500 -2.02763000 -0.05602800

H -1.99696500 1.35840900 2.77276500

H -3.10074100 2.64347500 2.21645000

C -0.20575900 3.58437600 2.64280000

C 1.05361600 3.41962500 0.58196200

C -2.72579800 4.46672200 -0.01831500

C -2.45925700 2.94386300 -1.88468200

H -2.15671500 -0.69898700 3.48392600

C -2.12030800 -2.73877700 4.15529300

C -2.52012200 -4.42975200 2.47795500

H -2.92832200 -3.71598100 0.48703300

H -3.08153900 -0.51779700 -2.29652000

C -4.98085300 -1.44273900 -2.78327500

C -6.18931500 -2.40328000 -0.92084100

H -5.23818600 -2.26217900 1.00824700

H -1.07676700 3.34863400 3.25572700

C 0.79882700 4.38725500 3.19415900

C 2.04768900 4.22978000 1.13248800

H 1.16869100 3.01596900 -0.42701600

H -2.54131300 4.73089300 1.02699200

C -3.41652700 5.35822300 -0.84147000

C -3.14663900 3.84106300 -2.70930200

H -2.05995400 2.00821100 -2.28356900

H -1.89330900 -2.45845400 5.18711300

C -2.23440800 -4.08691700 3.80328600

H -2.61214400 -5.48043100 2.19051300

H -4.90858600 -1.20669000 -3.84810300

C -6.10103000 -2.11537000 -2.28749000

H -7.06614400 -2.92329300 -0.52595500

H 0.69353000 4.75981300 4.21653200

C 1.92903500 4.71301600 2.44032600

H 2.92617700 4.48121000 0.53656300

H -3.78311900 6.30406500 -0.43370500

C -3.63049300 5.04443300 -2.18931000

H -3.29937600 3.59864900 -3.76418100

H -2.10227600 -4.86656700 4.55769000

H -6.90731700 -2.41238000 -2.96321300

H 2.71323300 5.34348700 2.86778100

H -4.16827400 5.74428000 -2.83448200

C 5.38427200 2.35919700 0.55821900

C 5.91753200 1.18056400 1.11275100

C 5.69134900 -0.05914800 0.52165000

C 4.91451400 -0.12456100 -0.63985600

C 4.37610500 1.02425500 -1.21965500

C 4.61937100 2.25955900 -0.61350700

H 6.52330400 1.23998500 2.02117100

H 6.11545200 -0.97069200 0.94719000

H 3.75195100 0.94797600 -2.11058200

H 4.19702600 3.15996900 -1.06618800

C 5.61073000 3.68656800 1.23645300

H 5.48102400 4.52803600 0.53954800

H 4.88993200 3.82442900 2.06071600

H 6.62097700 3.75124900 1.66936600

S 4.55897400 -1.73261600 -1.34582100

O 5.75081100 -2.57832500 -1.21958100

O 3.93180600 -1.50572900 -2.65109300

N 3.40443000 -2.37871600 -0.30237700

C 3.71645600 -3.53867100 0.52688000

H 4.77846000 -3.76924500 0.35435900

H 3.60561900 -3.28688400 1.59523200

C 2.89718500 -4.75230400 0.17399900

H 2.86388700 -4.98796900 -0.89654600

C 2.26150700 -5.52961100 1.05445600

H 2.27644300 -5.30845800 2.12717800

H 1.71132300 -6.42088100 0.74051700

C 2.12468500 -1.65202200 -0.21929900

H 2.24929400 -0.73467500 -0.80911700

H 1.89296500 -1.32492400 0.79431500

**TS18*-model**

C 7.11252900 0.32180900 -0.57652400

C 6.06462900 1.20256400 -0.89423000

C 4.73561500 0.78382600 -0.86559200

C 4.44534500 -0.53450800 -0.50266300

C 5.46513600 -1.43970100 -0.20211900

C 6.79081500 -1.00429300 -0.24371400

H 6.29316400 2.23629400 -1.16824200

H 3.92841100 1.46961600 -1.12377300

H 5.21174300 -2.46724500 0.06070900

H 7.59295900 -1.71084200 -0.01312400

C 8.54192400 0.80253400 -0.57173000

H 8.73781800 1.49473400 -1.40561400

H 8.76430300 1.34828500 0.36192600

H 9.25405000 -0.03302300 -0.64364400

S 2.73343200 -1.05192200 -0.35690100

O 2.69562500 -2.51452600 -0.43386500

O 1.97513600 -0.21685700 -1.29922200

N 2.28575200 -0.64418200 1.20621300

C 2.28676300 -1.70590600 2.22562500

H 1.88698500 -1.23200600 3.13220500

H 1.55287800 -2.47784200 1.95853700

C 3.63713400 -2.30329300 2.52181000

H 4.45011100 -1.60069200 2.73728100

C 3.87629300 -3.61641800 2.53666800

H 3.09190000 -4.33758100 2.28641400

H 4.86293300 -4.01612100 2.78862400

C 1.94962300 0.75301300 1.48665300

H 1.82132700 1.21051600 0.49468900

C 0.50071600 0.96473600 1.99808100

C 0.17615200 2.32105500 2.60274100

C -0.25083300 0.26889000 3.78112000

H -0.86570200 2.64163400 2.48953300

H 0.89765200 3.12848100 2.44223400

H -1.29438000 0.23924000 3.47019000

H 0.10964100 -0.63120100 4.28346500

H 1.49489000 1.46736000 4.18498400

Cl -1.06706700 -2.12950700 2.00322500

C -1.50735800 1.09521600 -2.34203700

C 0.22186200 2.96964900 -0.91971000

C -2.57881800 2.79760000 -0.28925700

C -2.66464700 0.10092000 -2.21441300

C -1.61984200 -2.54693600 -1.64556500

C -3.94076700 -1.61196300 -0.17844800

H -1.74601400 1.92933000 -3.02049000

H -0.60104300 0.59046700 -2.71092300

C 1.26552600 2.70400900 -1.82532800

C 0.24625700 4.18318500 -0.20791200

C -2.91955000 3.87790500 -1.12238700

C -3.41849800 2.48098400 0.78988000

H -2.81942600 -0.47212500 -3.14019800

H -3.60012200 0.63580700 -1.99361600

C -0.32226400 -2.97536600 -1.34140400

C -2.35547100 -3.22222200 -2.63616800

C -5.00843700 -0.70646300 -0.05516500

C -4.10018000 -2.92069600 0.30871300

H 1.29884600 1.75638500 -2.36139800

C 2.28927800 3.63574400 -2.02552500

C 1.27391800 5.11032200 -0.40398000

H -0.55389800 4.41529300 0.49780400

H -2.27175900 4.14573900 -1.96169700

C -4.07686600 4.62175200 -0.88185900

C -4.57765300 3.22703400 1.03253500

H -3.15620300 1.62525800 1.42061400

H 0.23647600 -2.47732300 -0.55408100

C 0.24968200 -4.04731400 -2.03285200

C -1.78895100 -4.30112000 -3.31768700

H -3.37852900 -2.91230300 -2.86863800

H -4.90034000 0.32869200 -0.38604500

C -6.22018100 -1.10958500 0.51197700

C -5.31190800 -3.31966500 0.87775500

H -3.26636100 -3.62231300 0.25582900

H 3.08558200 3.41319200 -2.74076700

C 2.29733200 4.84292800 -1.31898400

H 1.27106300 6.04777800 0.15817900

H -4.33263100 5.45807100 -1.53795900

C -4.90910200 4.29679600 0.19687300

H -5.22468500 2.96468800 1.87360900

H 1.27283900 -4.34070700 -1.78840200

C -0.48181100 -4.71076100 -3.02070400

H -2.36707200 -4.82424800 -4.08433000

H -7.04302200 -0.39423000 0.59397200

C -6.37699100 -2.41902200 0.97707100

H -5.42088800 -4.34091100 1.25210300

H 3.09791800 5.56982400 -1.47798700

H -5.81559500 4.87889900 0.38276400

H -0.03826200 -5.55090800 -3.56232000

H -7.32451400 -2.73451500 1.42184500

C 0.50670900 1.42248900 3.72716400

P -2.29213200 -1.06569200 -0.78573900

P -1.10660000 1.71046200 -0.62002000

Rh -0.93229200 -0.04779400 0.72392800

H 2.76126400 1.31404000 1.94989000

**TS21***

C 1.93576700 0.77702400 -1.85595500

C 3.35951200 1.87440000 0.00713100

C 0.94360400 2.24766700 1.02131000

H 2.19424300 0.52224100 -2.89490100

H 4.17239000 2.56053300 -0.26718000

H 1.40813000 1.73829000 -1.91705400

H 0.12199700 2.96617600 0.90248100

H 1.26648000 2.32571600 2.08075400

N 3.19488800 0.94192600 -1.13310200

S 4.61935100 0.41211400 -1.88602300

O 5.67593000 1.37800000 -1.56574500

O 4.28066800 0.08653800 -3.27637300

C 5.02773700 -1.12667800 -1.06887200

C 6.06616200 -1.16007700 -0.14132200

C 4.24339100 -2.25745900 -1.32283700

C 6.31017500 -2.34469000 0.55818000

H 6.65507900 -0.26031900 0.04035700

C 4.49053100 -3.42414400 -0.60550800

H 3.44721100 -2.21687400 -2.06830300

C 5.52224600 -3.48563700 0.35115300

H 7.11779800 -2.37309100 1.29416000

H 3.86994900 -4.30609600 -0.78571600

C 5.75005900 -4.75221500 1.13681500

H 4.88922400 -4.96323100 1.79371100

H 5.86449500 -5.62109600 0.46827700

H 6.64810500 -4.68583600 1.76818400

C 3.78771200 1.20838900 1.31548200

C 4.42958500 2.00207600 2.27891600

C 3.54685500 -0.13847000 1.60903100

C 4.81146800 1.46534100 3.51045700

H 4.63107300 3.05453100 2.05798700

C 3.92247700 -0.67913200 2.84173400

H 3.06751000 -0.78027800 0.87253100

C 4.55551700 0.11949800 3.79712600

H 5.31274200 2.09833500 4.24744400

H 3.71329400 -1.73151500 3.04662500

H 4.85179500 -0.30340600 4.76060100

C 2.11999100 2.74547100 0.19190400

C 2.10703500 3.98065300 -0.33056500

H 1.24402300 4.64270100 -0.21173100

H 2.96346000 4.37305900 -0.88646800

C 1.04034000 -0.30860000 -1.32059700

C 0.47586800 0.82386500 0.89182200

C -0.23722600 -0.56001500 -1.81566600

H 1.52944300 -1.11755500 -0.77120100

H -0.58117100 -1.59598500 -1.84818400

H -0.66179300 0.11285100 -2.56949600

H 1.17720200 0.11828600 1.35058700

C -4.20181200 1.12587200 -0.03838900

C -2.41023900 3.07624300 1.05106900

C -2.26332000 2.47727400 -1.78922400

C -4.39947400 -0.23079800 -0.71587300

C -3.81794200 -2.03839300 1.46102400

C -3.16635700 -2.82894700 -1.24702800

H -4.86767700 1.90629600 -0.43918900

H -4.40878600 1.05025000 1.04124300

C -2.24106500 2.77043400 2.41260300

C -2.58315300 4.41372500 0.66342300

C -1.01819900 3.03974200 -2.12271700

C -3.28528600 2.49488800 -2.75144000

H -5.40503000 -0.63468600 -0.52069100

H -4.26964800 -0.14776600 -1.80664200

C -3.23187500 -1.71207500 2.69307300

C -4.98464400 -2.82119900 1.43707000

C -4.19979700 -3.01017700 -2.18244100

C -2.12837100 -3.77840500 -1.18282100

H -2.07513200 1.73076200 2.70933100

C -2.25126400 3.78578900 3.37015000

C -2.58193200 5.43032900 1.62491400

H -2.71130600 4.66750500 -0.39110200

H -0.20890800 3.03391400 -1.39329600

C -0.79991700 3.60935800 -3.37699800

C -3.06381000 3.05631800 -4.01492000

H -4.26731600 2.07327200 -2.53184800

H -2.30464600 -1.13562700 2.70601200

C -3.81729900 -2.15026800 3.88541400

C -5.56701900 -3.25617000 2.62943800

H -5.43482200 -3.10475000 0.48157800

H -5.02098600 -2.29441900 -2.25554100

C -4.19784600 -4.11864100 -3.03581800

C -2.13384100 -4.88410800 -2.03636500

H -1.31616500 -3.63678200 -0.46302900

H -2.11622900 3.53727600 4.42585800

C -2.41709700 5.11918400 2.97748800

H -2.70974600 6.47022400 1.31317500

H 0.17632500 4.04198500 -3.61035700

C -1.82341300 3.61463600 -4.33188700

H -3.86958200 3.05641100 -4.75347600

H -3.35043700 -1.89755000 4.84090700

C -4.98460200 -2.91813000 3.85670900

H -6.47379800 -3.86615000 2.60143600

H -5.00865300 -4.24714100 -3.75783400

C -3.16593000 -5.05818900 -2.96462600

H -1.32194900 -5.61362400 -1.97640100

H -2.41277000 5.91550800 3.72615900

H -1.65278600 4.05069000 -5.31925900

H -5.43806400 -3.26224900 4.79008200

H -3.16549000 -5.92356100 -3.63249000

Cl -0.04597800 -2.18949400 1.18428400

Rh -1.08781000 -0.12000500 0.22386600

P -2.41456700 1.66626200 -0.13801700

P -3.07226100 -1.39310200 -0.09793200

**TS21-model-I**

C 0.38725300 -0.57074200 -0.53493100

C 1.43612200 -1.64330700 -0.35583900

C 0.15367500 1.38288200 1.12700700

C 3.16099700 -0.20314700 0.62166000

C 2.70242100 1.22742100 0.69552300

H 0.78109700 0.24552400 -1.16040000

H -0.52013200 1.57937900 1.97147600

H -0.45375300 -0.99714500 -1.11407200

H 3.60568800 1.76845400 1.04302300

H 2.49593600 1.62169800 -0.30938600

N -0.13144000 -0.00355100 0.71156600

S -1.14245400 -0.95112000 1.65656900

O -1.44555100 -0.18789400 2.87122200

O -0.57782000 -2.30510200 1.73906600

C -2.65095200 -1.08640600 0.69655200

C -3.62664800 -0.09000200 0.81661100

C -2.81507300 -2.15140300 -0.19161300

C -4.76705100 -0.16035100 0.01997900

H -3.48579900 0.72278700 1.53114500

C -3.96454500 -2.20308400 -0.98505600

H -2.05420600 -2.93193200 -0.24466300

C -4.95376300 -1.21213000 -0.89644800

H -5.53224300 0.61580000 0.11026200

H -4.09505800 -3.03376900 -1.68379400

C -6.20220700 -1.27070500 -1.74033900

H -6.14278100 -2.05947800 -2.50440500

H -7.08869500 -1.47352800 -1.11553700

H -6.38217400 -0.31114700 -2.25158700

C -0.20342200 2.39496900 0.03663700

C 0.47284800 3.61970200 -0.05546800

C -1.26591000 2.14965500 -0.84652000

C 0.10165100 4.57198000 -1.00889300

H 1.29864800 3.83121500 0.62678200

C -1.63899200 3.09997900 -1.79962600

H -1.80657700 1.20372100 -0.79107300

C -0.95475100 4.31592900 -1.88681000

H 0.64724200 5.51727300 -1.06851000

H -2.46724600 2.88543800 -2.48020500

H -1.24037600 5.05749400 -2.63717000

C 1.57322600 1.50522700 1.66242200

C 1.78213200 1.80466900 2.95129400

H 2.79518900 1.88942000 3.35760100

H 0.95128400 1.95714800 3.64621100

C 1.98692300 -2.32089800 -1.45729400

H 1.40402400 -2.16517800 0.59802500

H 2.24748200 -3.37684600 -1.33885100

H 1.69093800 -2.01952400 -2.46721800

H 3.10386700 -0.66408900 1.62212900

Rh 3.80778600 -1.19194200 -0.95380500

**TS21*-model-I**

C 1.17075700 -0.21448200 1.87809600

C 0.14506400 1.84725100 0.69505600

C 2.47510100 1.73585900 -0.55515000

H 0.89268300 -0.70711200 2.82209000

H -0.34846100 2.62435400 1.29450600

H 2.01315300 0.44264500 2.13249500

H 3.50619300 2.06361100 -0.36848900

H 2.14706800 2.26802200 -1.47273300

N 0.02062900 0.58442700 1.46091100

S -1.45721500 0.32411100 2.25141700

O -2.10754300 1.62857500 2.41670900

O -1.18668400 -0.54376700 3.40353600

C -2.43763900 -0.62606200 1.09455300

C -3.46498900 -0.00469400 0.38868800

C -2.10387200 -1.96537300 0.86388700

C -4.15797000 -0.73518500 -0.57997700

H -3.69674900 1.04266900 0.58544600

C -2.79420900 -2.67298300 -0.11566200

H -1.30895100 -2.44016100 1.44176900

C -3.82702400 -2.06876000 -0.85837700

H -4.95862700 -0.24913000 -1.14338400

H -2.52661000 -3.71439900 -0.31385400

C -4.53915400 -2.84775800 -1.93513700

H -3.84908400 -3.09133500 -2.76069800

H -4.92511100 -3.80425100 -1.54635300

H -5.38412100 -2.28402600 -2.35674900

C -0.56358200 1.84059600 -0.66011300

C -0.92423400 3.07593300 -1.22048700

C -0.84270500 0.67207100 -1.37764600

C -1.53890800 3.14244500 -2.47283100

H -0.71892700 3.99620500 -0.66549500

C -1.45303600 0.73347600 -2.63330900

H -0.59025600 -0.29958400 -0.95779100

C -1.80325300 1.96759100 -3.18614800

H -1.81498200 4.11359800 -2.89205900

H -1.65031100 -0.19465600 -3.17451100

H -2.28291000 2.01617200 -4.16721000

C 1.60433000 2.27681600 0.57123100

C 2.09127200 3.18686600 1.42768500

H 3.12792300 3.53171100 1.36723700

H 1.46567900 3.62339600 2.21161700

C 1.58266100 -1.28292900 0.90048000

C 2.40154100 0.28261000 -0.93648100

C 2.70661900 -2.08718000 1.07477300

H 0.80555900 -1.65283500 0.22613100

H 2.65321000 -3.12487600 0.73873200

H 3.38514700 -1.88569100 1.91159300

H 1.46914900 0.04224200 -1.45901700

Rh 3.55007900 -1.28463200 -0.85429000

**TS21-model-II**

C -0.61914100 -0.82339700 1.57026700

C -1.06690200 -2.12372800 2.19576200

C -1.60816000 0.01823800 -0.65385900

C -3.40251200 -2.00992800 1.46314200

C -3.67753800 -0.73962000 0.70620600

H -1.22977500 0.01093100 1.94926300

H -1.26013000 -0.01307100 -1.69484400

H 0.41009500 -0.60615500 1.91376900

H -4.77351900 -0.77169000 0.54139500

H -3.50506000 0.13824400 1.34459300

N -0.64730400 -0.80337600 0.10626200

S 0.50501100 -1.69569700 -0.72379900

O 0.20434500 -1.57321200 -2.15361200

O 0.62518400 -3.01572500 -0.08979000

C 2.04655400 -0.83640800 -0.40636000

C 2.41375200 0.23198400 -1.23275200

C 2.83604900 -1.20749300 0.68404700

C 3.57673400 0.94201300 -0.94392300

H 1.79126700 0.49208800 -2.09058700

C 3.99773700 -0.48095500 0.95996400

H 2.54425400 -2.06331800 1.29509300

C 4.38469100 0.60288700 0.15750300

H 3.86782500 1.77753200 -1.58677200

H 4.61675000 -0.76664600 1.81466300

C 5.64442000 1.38127400 0.44280100

H 6.09028000 1.09850300 1.40755100

H 6.40015600 1.20541900 -0.34161800

H 5.44666600 2.46527900 0.46120400

C -1.57525300 1.48638500 -0.22466700

C -2.71312300 2.29619700 -0.34817200

C -0.38411500 2.07171200 0.23097500

C -2.66418100 3.65378100 -0.01922400

H -3.64781900 1.86012600 -0.70641000

C -0.33268800 3.42818700 0.55967900

H 0.51341400 1.45998700 0.33305500

C -1.47428800 4.22586600 0.43811500

H -3.56497000 4.26539800 -0.11569900

H 0.60525000 3.86180800 0.91670200

H -1.43778800 5.28578100 0.70229100

C -2.99240000 -0.61447700 -0.63620900

C -3.53696000 -1.07203500 -1.77137100

H -4.52785300 -1.53747300 -1.77852800

H -3.00597600 -1.01379500 -2.72590300

H -0.96406800 -3.00068200 1.56045600

H -3.31417900 -2.85220500 0.75643300

H -3.30904400 -2.14380300 2.54084300

H -1.04712700 -2.26887100 3.25568800

**TS21*-model-II**

C -1.70925300 -2.00979500 0.97176500

C -1.81035300 0.05730900 -0.58362300

C -3.39051100 0.89590800 1.36685100

H -1.38861600 -3.04232700 0.76633000

H -1.96075000 -0.08493900 -1.66248400

H -2.80485300 -2.02743600 0.89749500

H -4.44123200 0.77445800 1.66121700

H -3.24925700 1.98130500 1.18051300

N -1.14897900 -1.17241400 -0.08652600

S 0.03886200 -1.84867400 -1.09071400

O -0.19757100 -1.35518700 -2.45175900

O 0.08725800 -3.28618900 -0.79919100

C 1.58118000 -1.14298000 -0.52005900

C 2.19654600 -0.13883600 -1.26357200

C 2.10493500 -1.56286400 0.70784100

C 3.34830500 0.46845300 -0.75681200

H 1.76025000 0.17208300 -2.21350300

C 3.24382700 -0.93600000 1.20458800

H 1.61940900 -2.36543900 1.26588100

C 3.88019300 0.09392900 0.48507300

H 3.82940700 1.26367200 -1.33204800

H 3.64815300 -1.24649000 2.17182800

C 5.09507100 0.77794800 1.05915800

H 4.83137400 1.33888400 1.97178700

H 5.86770400 0.04538200 1.34442300

H 5.54153600 1.48548300 0.34530500

C -0.97746800 1.33114200 -0.43408700

C -1.27111300 2.41268200 -1.27918300

C 0.03772100 1.48040500 0.51739900

C -0.57450900 3.61812800 -1.16848100

H -2.05699500 2.30554400 -2.03282700

C 0.73430900 2.68612600 0.63557800

H 0.29785000 0.65138900 1.17252300

C 0.43149700 3.75939500 -0.20568300

H -0.81541600 4.44865900 -1.83727700

H 1.51548800 2.77671200 1.39373500

H 0.97766600 4.70197700 -0.11540400

C -3.20451800 0.21608100 0.01672100

C -4.26917300 -0.19165800 -0.68973200

H -5.28700400 -0.08582200 -0.30238600

H -4.15753200 -0.63631500 -1.68285400

C -1.25818900 -1.65154800 2.36287200

C -2.44575600 0.59945000 2.49924100

H -0.29618000 -1.13875300 2.44680000

H -1.45975600 1.04937900 2.33941800

H -2.48249100 0.10687300 3.47089900

H -1.64163100 -2.13723500 3.26019000

**TS21-model-III**

C -1.80896400 -0.64955600 0.30781200

C -2.24818500 0.55805100 -0.48677100

C 0.65389500 -1.39973100 0.37713600

C -0.21541700 1.88400300 -0.14844600

C 0.76830600 1.16300500 0.73173900

H -1.56050000 -0.35593500 1.33964000

H 1.22195600 -2.17605400 -0.15237900

H -2.67046000 -1.33740700 0.40372800

H 1.57026000 1.91266800 0.88743400

H 0.34002300 0.98419300 1.72799800

N -0.67563200 -1.38148900 -0.26169300

C 1.38456500 -0.08548300 0.14126800

C 2.50054600 -0.06023700 -0.59930300

H 3.03628200 0.87582800 -0.78724800

H 2.90570100 -0.96764000 -1.05639800

H -1.99358200 0.53210900 -1.54400400

H 0.10893800 1.81780700 -1.20062600

H -1.11727900 2.41921000 0.14870200

H -3.09274300 1.14414800 -0.18993200

H -0.83017900 -1.93091200 -1.10706300

H 0.60252500 -1.70790200 1.42140200

**TS21*-model-III**

C -1.37416400 -0.79509000 -0.69071600

C 0.59696800 -1.01058600 0.97378700

C 1.01071700 1.45956700 0.11841800

H -2.00218400 -1.56923000 -1.15701200

H 1.21306300 -1.92007800 0.96706700

H -0.61454800 -0.54698800 -1.44410600

H 1.48634700 1.94457200 -0.74411400

H 1.57256600 1.80564900 1.01134200

N -0.74153200 -1.40691100 0.47570800

C 1.27689500 -0.03648900 0.01558400

C 2.16181900 -0.50829500 -0.87499200

H 2.68086700 0.15618300 -1.57232800

H 2.40262300 -1.57392200 -0.92908900

C -2.25021400 0.38997400 -0.38227200

C -0.38642100 1.96452100 0.35460200

H -2.67357300 0.43458900 0.62482300

H -0.72479400 1.77157400 1.37854100

H -1.14369500 2.52507700 -0.19349800

H -2.73299500 0.99366300 -1.15076500

H 0.60774000 -0.64427400 2.00033400

H -1.09429000 -2.31005800 0.79238900

**Rh-I**

Rh 1.70826100 0.00002600 -0.05452600

Cl -0.00004600 -1.65279400 -0.69944900

Rh -1.70830500 0.00001500 -0.05466900

Cl 0.00005500 1.65265500 -0.69967000

C -3.20964200 1.50252700 -0.21542400

H -2.84416900 2.22619900 -0.95121700

H -4.20090800 1.09049600 -0.42730100

C -2.69941500 1.49744100 1.09096100

H -3.28733000 1.08544300 1.91664600

H -1.92569600 2.21406300 1.38437200

C 3.20977900 1.50235200 -0.21552000

H 2.84413900 2.22602000 -0.95123900

H 4.20104100 1.09042700 -0.42759500

C 2.69971500 1.49732100 1.09093500

H 3.28765000 1.08528600 1.91659800

H 1.92616400 2.21409800 1.38441000

C -2.69979200 -1.49719700 1.09103500

H -1.92616000 -2.21379000 1.38473600

H -3.28783600 -1.08496900 1.91651200

C -3.20971500 -1.50251300 -0.21546000

H -4.20088400 -1.09046200 -0.42771200

H -2.84402300 -2.22628400 -0.95104700

C 3.20958700 -1.50249700 -0.21568000

H 4.20083700 -1.09058700 -0.42782900

H 2.84380900 -2.22604600 -0.95143100

C 2.69970300 -1.49742000 1.09083800

H 1.92603000 -2.21402600 1.38440300

H 3.28784200 -1.08546200 1.91638900

**PPh_3_**

C 1.48030200 0.74820200 -0.43897900

C 1.41969200 1.58091200 0.69085000

H 0.45322300 1.82331100 1.13785200

C 2.59018200 2.10184000 1.25246000

H 2.52637600 2.74982800 2.13081400

C 3.83633800 1.79379400 0.69885500

H 4.74968300 2.20100300 1.14029300

C 3.90812100 0.96617400 -0.42700500

H 4.87816900 0.72439100 -0.86943200

C 2.73882400 0.45611500 -0.99574700

H 2.80249600 -0.17983100 -1.88394000

C -1.38799400 0.90804700 -0.43880400

C -1.76475100 2.14384100 -0.99558100

H -1.24623000 2.51693500 -1.88400500

C -2.79105800 2.90133700 -0.42654200

H -3.06705200 3.86220100 -0.86899200

C -3.47129700 2.42542100 0.69970300

H -4.28049300 3.01274200 1.14145100

C -3.11457100 1.19231700 1.25333200

H -3.64341300 0.81312200 2.13197000

C -2.07832700 0.43919600 0.69136700

H -1.80474100 -0.51894800 1.13830500

C -0.09243200 -1.65598000 -0.43931300

C -0.98020700 -2.59717100 -0.99186000

C 0.66468300 -2.02282700 0.68574100

C -1.12309700 -3.86495100 -0.42343800

H -1.56734500 -2.33205800 -1.87636800

C 0.53053100 -3.29716100 1.24698900

H 1.36239000 -1.30934500 1.12935600

C -0.36492000 -4.21952300 0.69781100

H -1.82195000 -4.58194000 -0.86244800

H 1.12800400 -3.56819600 2.12165200

H -0.46899000 -5.21420900 1.13900200

P -0.00001600 0.00023300 -1.26696900

**CH_2_=CH_2_**

C 0.00000000 0.66660200 0.00000000

H 0.93133400 1.24257800 0.00000000

H -0.93128700 1.24263200 0.00000000

C 0.00000000 -0.66660200 0.00000000

H -0.93133400 -1.24257800 0.00000000

H 0.93128700 -1.24263200 0.00000000

**Rh-II**

Rh -1.68598500 0.00002400 -0.08089500

C -2.88288900 1.33251700 0.41528100

C -2.88294100 -1.33248800 0.41510700

O -3.60268100 2.16682100 0.71798100

O -3.60308900 -2.16650300 0.71776500

Cl -0.00001600 1.64015200 -0.75427700

Cl -0.00008900 -1.64006900 -0.75460700

Rh 1.68598300 0.00008000 -0.08108100

C 2.88336700 1.33210100 0.41515500

C 2.88270800 -1.33254500 0.41529300

O 3.60350200 2.16612400 0.71782600

O 3.60231800 -2.16689400 0.71829700

**dppe**

C 0.65320400 0.40099400 -0.98787900

H 0.42769000 1.47806700 -1.05013200

H 1.25147900 0.13403400 -1.87508500

C -0.65320500 -0.40113100 -0.98787300

H -0.42768600 -1.47820500 -1.05005000

H -1.25147600 -0.13422600 -1.87509700

C 1.92875800 -1.68676200 0.45107300

C 1.25241500 -2.48943500 1.38451500

H 0.65300100 -2.01543000 2.16611900

C 1.32629800 -3.88498400 1.31069000

H 0.79152100 -4.49657600 2.04214900

C 2.08422600 -4.49382800 0.30701000

H 2.14618100 -5.58384100 0.25066500

C 2.76666100 -3.70318400 -0.62564100

H 3.36226800 -4.17514900 -1.41181500

C 2.68771700 -2.31056200 -0.55566800

H 3.22656700 -1.70086900 -1.28581400

C 3.33828900 0.77442900 -0.01474400

C 3.46723100 1.80344700 -0.96448500

H 2.58507000 2.21803600 -1.45679500

C 4.72543700 2.31816800 -1.29680600

H 4.80201700 3.11614800 -2.04037800

C 5.87762900 1.81552200 -0.68808000

H 6.85998800 2.21584800 -0.95125800

C 5.76275700 0.79541000 0.26341400

H 6.65725000 0.39377300 0.74715400

C 4.50786800 0.28618300 0.60046500

H 4.43538300 -0.51219200 1.34432700

C -3.33832700 -0.77436900 -0.01476500

C -3.46732700 -1.80351200 -0.96436900

H -2.58518400 -2.21824400 -1.45658700

C -4.72556400 -2.31816600 -1.29667200

H -4.80218700 -3.11623600 -2.04014200

C -5.87773700 -1.81533200 -0.68806300

H -6.86012000 -2.21560800 -0.95122700

C -5.76281000 -0.79510900 0.26330100

H -6.65728300 -0.39332400 0.74695400

C -4.50788800 -0.28594000 0.60033000

H -4.43537100 0.51253600 1.34408000

C -1.92868600 1.68669100 0.45108200

C -2.68738600 2.31050200 -0.55584500

H -3.22605600 1.70081000 -1.28612900

C -2.76633500 3.70312400 -0.62581700

H -3.36174100 4.17509100 -1.41214100

C -2.08416000 4.49375700 0.30703300

H -2.14610700 5.58377000 0.25068400

C -1.32648600 3.88490500 1.31089900

H -0.79190900 4.49648600 2.04251200

C -1.25259500 2.48935400 1.38471000

H -0.65337300 2.01534400 2.16645500

P 1.69566100 0.15011100 0.56115300

P -1.69564900 -0.15018200 0.56115600

**CO**

C 0.00000000 0.00000000 -0.64593900

O 0.00000000 0.00000000 0.48445400

**TS-S1**

C -6.34742600 -1.56332900 1.10790500

C -5.60811900 -0.43179300 1.50171400

C -4.23575100 -0.35422100 1.28048800

C -3.59021000 -1.41948100 0.64253000

C -4.29266100 -2.55451300 0.24290900

C -5.66843700 -2.61855600 0.48102800

H -6.11909800 0.40190000 1.99150700

H -3.67130300 0.52612600 1.59211600

H -3.76106800 -3.36591600 -0.25532200

H -6.22401800 -3.50705900 0.16893100

C -7.82905600 -1.63556600 1.38009800

H -8.33517600 -0.70032500 1.09146900

H -8.30456600 -2.46504900 0.83634500

H -8.02205000 -1.78734200 2.45609100

S -1.82803600 -1.31042500 0.32894800

O -1.40987300 -2.56274200 -0.30185300

O -1.21568700 -0.82208800 1.57328900

N -1.69625900 -0.07027200 -0.81336000

C -1.73689800 -0.48423200 -2.22414100

H -1.56510100 0.42841900 -2.81120400

H -0.90863000 -1.17948800 -2.45080900

C -3.05528200 -1.07893000 -2.64356700

H -3.94460600 -0.46657300 -2.45131100

C -3.17813600 -2.27900000 -3.21340500

H -2.30627700 -2.92077300 -3.37423900

H -4.15266500 -2.66572100 -3.52430500

C -1.18260100 1.22561800 -0.36454100

H -0.81110900 1.04799200 0.65311300

C -2.28871000 2.26391800 -0.22555900

C -3.30962800 2.40839200 -1.17938900

C -2.30458400 3.09877900 0.90256200

C -4.30973900 3.36891600 -1.01517100

H -3.33527500 1.75671500 -2.05361800

C -3.30486400 4.06296800 1.06925800

H -1.53269000 2.97970800 1.66790300

C -4.31034900 4.20245200 0.10954900

H -5.09839000 3.46292900 -1.76613100

H -3.30202200 4.69897000 1.95816600

H -5.09611100 4.95076100 0.23904700

C 0.15480600 1.64241300 -1.02675100

C 0.75517100 3.00417600 -0.63820500

C 0.32334700 2.27198100 -2.86035900

H 1.84631800 3.03410800 -0.68812500

H 0.35367200 3.50036000 0.25079800

H 1.36516900 2.00154600 -3.07497700

H -0.41362000 2.00917400 -3.62591900

H -1.01127100 3.63336800 -1.84129700

Cl 2.18438000 -1.77764900 -2.81986600

C -0.01228000 3.19709100 -1.87242100

Rh 1.35800800 0.05302400 -1.61393400

P 2.48614400 -0.16116300 0.25418800

C 2.07223500 -1.66774400 1.25001500

C 4.32766000 -0.27433000 0.05214400

C 2.30730600 1.21606000 1.47965600

C 1.55094500 -2.78861600 0.58679800

C 2.27105700 -1.72789100 2.64169500

C 4.90311900 -0.17268800 -1.22258400

C 5.16399000 -0.45298600 1.16849500

C 1.17467500 1.27271600 2.31400100

C 3.20579200 2.29618600 1.48492800

H 1.38122500 -2.75384600 -0.48988400

C 1.22763400 -3.94321900 1.30423000

C 1.94820500 -2.88451500 3.35368100

H 2.66006400 -0.86448700 3.18477400

H 4.25524700 -0.06079900 -2.09375400

C 6.29247200 -0.24205600 -1.37647800

C 6.54945500 -0.51831000 1.01253200

H 4.73497000 -0.54259800 2.16816100

C 0.94586800 2.39017900 3.12142700

H 0.47239000 0.43721800 2.33572500

C 2.97183100 3.41444400 2.29181800

H 4.09427000 2.26664400 0.84981200

H 0.80248400 -4.79818100 0.77350000

C 1.42271900 -3.99567200 2.68580100

H 2.10069400 -2.91395800 4.43594100

H 6.72772000 -0.16900800 -2.37651300

C 7.11763300 -0.41192900 -0.26257600

H 7.18801700 -0.65726800 1.88895800

C 1.83809600 3.46908800 3.10835000

H 0.06664300 2.41230600 3.77147400

H 3.68128400 4.24616000 2.28230600

H 1.15941300 -4.89716800 3.24571700

H 8.20269600 -0.46715700 -0.38497200

H 1.65693800 4.34225500 3.74052100

**TS-S2**

Cl -1.65235000 2.24437400 -2.05001700

C 1.36395300 -2.12667000 -0.83921600

C 1.44830700 -2.47837400 -2.20419700

C 0.18054700 -2.86487900 -0.31529700

C -0.07625800 -1.89798100 -1.45136600

H 1.28396400 -3.50977700 -2.52736300

H 1.92096200 -1.79329400 -2.90800700

H 0.13425700 -3.94803000 -0.49693600

H -0.18449000 -2.55661800 0.66658000

H -0.65152500 -2.11685600 -2.36888000

C 2.10122800 4.34178800 1.17660700

C 3.20330800 3.46670400 1.24251800

C 3.35124200 2.42871100 0.32770700

C 2.37845500 2.25696300 -0.66702100

C 1.28451700 3.11190600 -0.76937700

C 1.15668600 4.14778300 0.16069500

H 3.95343800 3.60127500 2.02684800

H 4.20264600 1.74936000 0.39840700

H 0.50834200 2.95605500 -1.52413700

H 0.28528100 4.80418800 0.09048100

C 1.93805300 5.43385200 2.20289500

H 2.87687100 5.99193600 2.34801900

H 1.15307500 6.14842000 1.91605200

H 1.66195300 5.00930400 3.18406800

S 2.60399900 0.95525200 -1.87237800

O 3.71180000 1.28440800 -2.76959600

O 1.29103500 0.63113700 -2.47854200

N 3.11556500 -0.35229300 -0.95094400

C 4.49856000 -0.83565500 -1.08422800

H 5.10782200 0.01483400 -1.42173900

H 4.85650800 -1.12288500 -0.08433100

C 4.66800400 -1.97150200 -2.05787300

H 4.39664300 -1.73713800 -3.09396000

C 5.13759000 -3.17977700 -1.73732900

H 5.41562900 -3.42611600 -0.70692000

H 5.26975400 -3.96299200 -2.48910500

C 2.12124300 -1.02737700 -0.10370800

H 1.35933800 -0.27759700 0.16651700

C 2.74004600 -1.51743700 1.19590000

C 2.90139400 -0.60506900 2.24837800

C 3.17433600 -2.83916300 1.36645700

C 3.49458200 -1.00505500 3.44758500

H 2.54310100 0.41939500 2.13039000

C 3.76541500 -3.24164700 2.56797200

H 3.05463200 -3.55828300 0.55338800

C 3.92947900 -2.32459800 3.61081700

H 3.60498500 -0.28558700 4.26290700

H 4.09912300 -4.27533800 2.68915000

H 4.39006900 -2.63930300 4.55064900

Rh -0.83760900 0.03951500 -1.58870700

P -2.34338100 -0.26534300 -0.04229400

C -1.46109200 0.21496000 1.51713400

C -3.88231600 0.75859700 -0.00728400

C -3.10280100 -1.92443100 0.30330100

C -1.06265000 1.56505900 1.61419100

C -1.04214300 -0.68553700 2.51032000

C -4.57062900 0.95155900 -1.21479400

C -4.42052800 1.27145600 1.18200800

C -3.14401500 -2.88462500 -0.71797700

C -3.74668700 -2.20754300 1.52118000

H -1.34604900 2.26362100 0.82191200

C -0.28537500 1.99700500 2.68798400

C -0.24855900 -0.24959700 3.57882000

H -1.33168600 -1.73665600 2.46175400

H -4.13120700 0.58583100 -2.14490900

C -5.78830000 1.63382100 -1.22772600

C -5.63633300 1.96285700 1.16471400

H -3.88836100 1.14395000 2.12751200

C -3.78232600 -4.11302200 -0.51975500

H -2.68898400 -2.65506300 -1.68279700

C -4.37855900 -3.43646500 1.72342800

H -3.76698600 -1.45831400 2.31532000

H 0.01531800 3.04526500 2.74431000

C 0.12638800 1.09137200 3.67511300

H 0.08123200 -0.96784200 4.33354700

H -6.31410600 1.78415700 -2.17410400

C -6.32381000 2.14171600 -0.03888600

H -6.04491300 2.36451300 2.09593800

C -4.39300000 -4.39584100 0.70479800

H -3.80762300 -4.84825300 -1.32839700

H -4.87097000 -3.64217900 2.67749800

H 0.74579700 1.43039200 4.50971700

H -7.27314700 2.68381000 -0.05243400

H -4.89093800 -5.35610400 0.86241400

**TS-S3**

C 6.79349600 1.12201200 0.51853500

C 5.85857100 0.77881500 1.51021500

C 4.49880100 1.03250500 1.34066000

C 4.06579700 1.64020000 0.15902400

C 4.97025100 2.00689600 -0.84045300

C 6.32771400 1.74081700 -0.65396100

H 6.20068100 0.29465900 2.42877900

H 3.77202300 0.75481300 2.10505100

H 4.61098200 2.50297100 -1.74401300

H 7.04079700 2.02473200 -1.43269200

C 8.26362900 0.86013800 0.72817700

H 8.81251700 0.82949600 -0.22482500

H 8.71659100 1.65592300 1.34479500

H 8.43138400 -0.09228300 1.25464900

S 2.31296800 1.85997800 -0.11863200

O 2.14110400 2.96555900 -1.06737200

O 1.66768700 1.87686300 1.20228500

N 1.78691600 0.47575100 -0.88318000

C 1.76430600 0.31927800 -2.33205800

H 2.10935700 1.25802500 -2.78973300

H 2.44214900 -0.49066800 -2.64876600

C 0.34747600 0.02275200 -2.77687200

C -0.66627900 0.99634900 -2.48877100

H -1.40389300 1.22355400 -3.26302100

H -0.38378200 1.84108400 -1.85386900

C 1.27162900 -0.66753700 -0.13743500

H 0.76872700 -0.28284800 0.76336500

C 2.36164600 -1.61922100 0.35216900

C 2.09872200 -2.42493700 1.47016200

C 3.62694900 -1.69591700 -0.24602300

C 3.06597100 -3.30452700 1.96305200

H 1.12899900 -2.35122800 1.96662900

C 4.59848300 -2.57118000 0.24668100

H 3.87203000 -1.04768900 -1.08781100

C 4.32046400 -3.38376500 1.34988700

H 2.84101100 -3.92231100 2.83634800

H 5.58161000 -2.60836400 -0.22922200

H 5.08081900 -4.06714300 1.73636900

C 0.13159100 -1.27309100 -0.97477400

C 0.15377300 -2.76555400 -1.29091300

H -0.83998000 -3.06741200 -1.67375600

H 0.30328400 -3.31225700 -0.34282600

C -1.69638100 -0.37870900 1.89686000

C -1.81153200 -1.74368300 2.22742300

H -2.43474000 -2.39976900 1.61331900

C -1.15370900 -2.26449900 3.34108700

H -1.26543900 -3.32279800 3.59067800

C -0.35027600 -1.43395400 4.13467100

H 0.17202900 -1.84212500 5.00354200

C -0.21495400 -0.08590900 3.80188500

H 0.41864600 0.57016300 4.40342900

C -0.88868000 0.44466200 2.69314700

H -0.76539100 1.50097700 2.45978600

C -2.63671500 2.00731700 0.39012100

C -3.84583600 2.71849500 0.32529300

H -4.79887700 2.18717400 0.33961000

C -3.83328600 4.11381700 0.23021200

H -4.78031400 4.65684400 0.17573800

C -2.62230600 4.81194200 0.19781900

H -2.62006400 5.90216700 0.11937900

C -1.41467000 4.10826700 0.25639200

H -0.45483800 4.62909000 0.21894000

C -1.42400400 2.71627000 0.34376700

H -0.47470400 2.18743700 0.37982800

C -4.31878400 -0.32469100 0.81023100

C -5.15181600 -0.93297900 -0.13954500

C -4.80518700 -0.08233100 2.10928500

C -6.45781900 -1.29670500 0.21241000

H -4.78579300 -1.09555100 -1.15702100

C -6.10770800 -0.44561900 2.45155800

H -4.16201100 0.38993700 2.85607900

C -6.93667000 -1.05686000 1.50202700

H -7.10196100 -1.76846300 -0.53402800

H -6.47752100 -0.25388800 3.46224400

H -7.95663000 -1.34387900 1.77166900

Cl -3.43877400 -0.47832900 -3.06970900

C 1.20495000 -3.18107400 -2.29481900

C 0.94549500 -3.41868000 -3.58394600

H 1.73665900 -3.70622300 -4.28225000

H -0.06989200 -3.33413700 -3.98724600

H 2.23056500 -3.26156700 -1.92092700

H 0.26227000 -0.58169500 -3.68563300

Rh -1.56358400 -0.60113400 -1.49292300

P -2.59477900 0.17079700 0.37173200

**TS-S4**

C -6.67628900 -0.77698400 0.36192200

C -5.71402800 -0.89208600 1.38023400

C -4.40838400 -1.28553100 1.09894300

C -4.05780100 -1.57072200 -0.22371600

C -4.99703100 -1.49499800 -1.25482200

C -6.30048800 -1.09475600 -0.95315700

H -5.98990000 -0.65525200 2.41112000

H -3.65559700 -1.35834300 1.88405700

H -4.70921900 -1.75836200 -2.27435900

H -7.04102300 -1.03173400 -1.75506400

C -8.07074800 -0.30585900 0.68823200

H -8.50425200 -0.88598600 1.51882500

H -8.05800900 0.75071100 1.00654700

H -8.74565600 -0.38898500 -0.17611700

S -2.34994300 -1.92288400 -0.61994000

O -2.33228200 -2.82636400 -1.77578800

O -1.66589200 -2.25058700 0.63616200

N -1.69805800 -0.48685700 -1.15250900

C -1.79164500 -0.05217000 -2.54487100

H -1.47604500 -0.86832000 -3.21084200

H -2.81959100 0.24611500 -2.82795400

C -0.85748700 1.14331000 -2.57861600

C 0.59790900 0.87648100 -2.31210000

H 1.28526400 1.53808600 -2.84699300

H 0.87202400 -0.17660800 -2.28405700

C -1.26009600 0.56356300 -0.22071600

H -0.58774900 0.11133600 0.51611500

C -2.38504500 1.25052300 0.53272700

C -2.28569800 1.39781000 1.92201400

C -3.52386400 1.74195000 -0.12505900

C -3.30881800 2.02019600 2.64523800

H -1.40278900 1.00976500 2.43670100

C -4.54816200 2.35680300 0.59523600

H -3.62114500 1.62668200 -1.20610800

C -4.44343600 2.49809300 1.98427700

H -3.21988700 2.12608700 3.72937700

H -5.43611400 2.72130800 0.07270700

H -5.24644200 2.97895300 2.54863800

C -0.50056300 1.53825600 -1.16824500

C -0.34712300 3.02357700 -0.80930600

H 0.60726100 3.24910900 -0.23327200

H -1.12074900 3.28095400 -0.07103100

C 1.87462800 -1.07929400 1.76091400

C 1.35965700 -0.22333800 2.75162000

H 1.41683900 0.85798500 2.61062400

C 0.79191200 -0.75194000 3.91370600

H 0.39748600 -0.07432700 4.67570200

C 0.72624700 -2.13577400 4.10276200

H 0.26932800 -2.54693900 5.00672600

C 1.24887800 -2.99175500 3.13009800

H 1.20308400 -4.07483200 3.26862000

C 1.82400000 -2.46859200 1.96910000

H 2.21641900 -3.15374000 1.21569200

C 2.40041400 -1.65893000 -1.04958200

C 3.38713300 -1.84909000 -2.03104100

H 4.33406800 -1.30842900 -1.96841600

C 3.16146400 -2.72853000 -3.09480600

H 3.93884000 -2.87124000 -3.85008800

C 1.94895200 -3.41872000 -3.19715900

H 1.77405400 -4.09801200 -4.03559600

C 0.96085300 -3.23517600 -2.22386800

H -0.00295300 -3.74472000 -2.28990300

C 1.18905100 -2.36489500 -1.15533700

H 0.41339500 -2.23644000 -0.40166600

C 4.40117400 -0.27608200 0.49187100

C 5.14904700 0.74151300 -0.11979200

C 5.06262500 -1.26531500 1.23618800

C 6.54147300 0.75913400 -0.00066800

H 4.63121800 1.52923500 -0.67017400

C 6.45406100 -1.24028400 1.35994700

H 4.49355000 -2.05593800 1.72937500

C 7.19618600 -0.23028700 0.73880900

H 7.11492900 1.55822000 -0.47731500

H 6.95972500 -2.01161700 1.94667600

H 8.28470300 -0.21070300 0.83829800

Cl 2.80595800 2.71612800 1.34369400

C -0.38991000 3.97759700 -1.97180300

C 0.69033600 4.52435900 -2.53570900

H 0.60904500 5.20964900 -3.38374900

H 1.69472000 4.30579400 -2.15586600

H -1.38827200 4.20654700 -2.36361400

H -1.09181300 1.92673200 -3.30317500

Rh 1.55789800 1.59387400 -0.29436600

P 2.57794300 -0.33092200 0.22107100

**TS-S5**

C 0.97980000 -2.70844600 -2.13261400

C 0.06249700 -3.26754900 -0.18567800

C -0.38611600 -2.66362300 -1.49617400

H 1.45314000 -3.69612900 -2.25717400

H 1.21729200 -2.00586100 -2.94070300

H 0.49109900 -4.28220300 -0.22078800

H -0.54038800 -3.06645200 0.69989800

Rh -0.58012000 -0.30934800 -1.58063200

H -1.39468000 -2.51117800 -1.89416100

Cl -0.81982700 1.72569800 -2.67609600

C 0.89765700 -2.14728900 -0.73320600

C 2.97264900 3.90019500 0.53569400

C 4.02113300 3.04240100 0.92178800

C 4.26842400 1.85551600 0.23788000

C 3.45450200 1.51797900 -0.84945500

C 2.42087000 2.35402000 -1.27083800

C 2.19069400 3.54237600 -0.57162200

H 4.64866400 3.31076700 1.77614100

H 5.07904500 1.19108200 0.54403800

H 1.77829800 2.07190600 -2.10646100

H 1.37488500 4.19205400 -0.89878100

C 2.68472900 5.15297700 1.32370300

H 2.13778600 4.90841100 2.25093200

H 3.61252400 5.66675400 1.62020900

H 2.06714300 5.86075800 0.75118300

S 3.68094600 -0.07722900 -1.63793600

O 5.11027000 -0.33101200 -1.83676500

O 2.73619600 -0.13294100 -2.76129800

N 3.16323200 -1.15611000 -0.45184800

C 4.07937300 -2.12052900 0.14971800

H 5.09317000 -1.82990300 -0.16507800

H 4.03948000 -2.03395100 1.24853500

C 3.82250300 -3.53873200 -0.28554400

H 3.79264400 -3.68763800 -1.37101700

C 3.65790500 -4.57497800 0.54070400

H 3.68311000 -4.44747000 1.62843700

H 3.50562800 -5.59043200 0.16481400

C 1.74396000 -1.03607400 -0.08892800

H 1.42026500 -0.08397000 -0.53554900

C 1.54205000 -0.86156100 1.40555900

C 1.33254500 0.43210300 1.90609500

C 1.58130800 -1.93697100 2.30582100

C 1.13730200 0.64768300 3.27205500

H 1.30508100 1.27777600 1.21823700

C 1.40264400 -1.72219200 3.67573500

H 1.74097500 -2.94905300 1.93256000

C 1.17065100 -0.43104200 4.16201400

H 0.94233000 1.66002200 3.63308700

H 1.43496600 -2.56981500 4.36525200

H 1.01464000 -0.26682400 5.23127900

P -2.16384300 0.08805500 -0.11867300

C -1.88279200 1.49378300 1.06152000

C -3.81353100 0.47249300 -0.86982700

C -2.55281600 -1.34308500 0.97887100

C -1.05530400 2.55807000 0.66826900

C -2.48403500 1.53927300 2.33302900

C -3.99883200 0.33827600 -2.25367400

C -4.90079700 0.85698100 -0.06626300

C -1.85340300 -1.55521200 2.17959400

C -3.46261600 -2.32022800 0.53900900

H -0.59078900 2.54171500 -0.31812900

C -0.82627200 3.63329300 1.53064500

C -2.24794400 2.61328700 3.19507300

H -3.12989100 0.72715800 2.67027900

H -3.15090300 0.05871200 -2.88156700

C -5.25345900 0.57998700 -2.82337800

C -6.15122400 1.09827700 -0.63772600

H -4.77715500 0.97302300 1.01181600

C -2.07730100 -2.70986300 2.93379500

H -1.12008600 -0.82745800 2.52603300

C -3.67407100 -3.47962300 1.28871300

H -4.01040600 -2.17328700 -0.39494400

H -0.17588700 4.44913300 1.20838000

C -1.41451800 3.66335200 2.79819000

H -2.71740400 2.62609700 4.18210100

H -5.38342700 0.47725700 -3.90365100

C -6.33050700 0.95916500 -2.01894200

H -6.98856300 1.39869000 -0.00252900

C -2.98510700 -3.67629200 2.49063300

H -1.52233600 -2.85556300 3.86347400

H -4.38335900 -4.23158500 0.93375700

H -1.22786800 4.50264100 3.47339800

H -7.30946400 1.15111300 -2.46598500

H -3.15422900 -4.58243800 3.07785100

**TS-S6**

C 2.67960900 4.21901700 1.28151800

C 2.01276500 4.22917200 0.04541300

C 2.08934400 3.14735400 -0.83105900

C 2.83987900 2.03099300 -0.45377000

C 3.51117800 1.98471000 0.77145100

C 3.42488000 3.07978300 1.63101500

H 1.39979200 5.09020400 -0.23332000

H 1.52524100 3.14485400 -1.76362000

H 4.07166900 1.09416000 1.06104900

H 3.93651300 3.04399900 2.59637900

C 2.58492800 5.40546000 2.20744800

H 3.17432500 6.25299200 1.81800100

H 1.54423400 5.75520200 2.30131500

H 2.96201400 5.17103300 3.21389400

S 2.99537400 0.67285400 -1.60748500

O 4.28509900 0.73276900 -2.29523100

O 1.75869900 0.67230800 -2.43332100

N 3.01745200 -0.68551100 -0.62583600

C 4.19883600 -1.55825200 -0.63259500

H 5.07334200 -0.90921600 -0.78899300

H 4.29249700 -1.99556600 0.37311200

C 4.19576200 -2.63169900 -1.68691700

H 4.06710600 -2.26994000 -2.71287700

C 4.36989700 -3.93296900 -1.44375700

H 4.50332900 -4.31039000 -0.42404300

H 4.39595800 -4.66896000 -2.25205900

C 1.70566200 -1.10062500 -0.10184100

H 1.17174200 -0.15676000 0.09459600

C 1.79714600 -1.77572900 1.25380700

C 1.58623100 -0.99780200 2.40315000

C 2.04553200 -3.14855600 1.39983800

C 1.61430600 -1.58021500 3.67323600

H 1.37082700 0.06893700 2.29977500

C 2.07779400 -3.73179800 2.66935000

H 2.20447200 -3.76380300 0.51315200

C 1.85831500 -2.95055500 3.80937100

H 1.43220300 -0.96175300 4.55566900

H 2.26908800 -4.80339600 2.76872200

H 1.87504500 -3.41009700 4.80096500

Rh -0.42631000 0.12137100 -1.52736100

P -2.08966400 0.07045100 -0.06634900

C -2.58980600 -1.56916300 0.63753100

C -1.89779400 -2.12727100 1.72862000

H -1.10381700 -1.56547700 2.22221800

C -2.19258200 -3.41737200 2.17463700

H -1.63663000 -3.83283800 3.01877000

C -3.17560900 -4.17565000 1.53047500

H -3.40313100 -5.18789600 1.87433500

C -3.86502800 -3.63196500 0.44181600

H -4.63527400 -4.21763000 -0.06662600

C -3.57714900 -2.33753500 -0.00058400

H -4.12505000 -1.92419600 -0.85037400

C -1.71355400 1.02880800 1.48628700

C -0.66641300 1.96573400 1.49329200

H -0.11378700 2.16352700 0.57545600

C -0.33527400 2.65320600 2.66522100

H 0.48773900 3.36945400 2.64574500

C -1.04372400 2.42189000 3.84701700

H -0.77957800 2.95775800 4.76260100

C -2.09424900 1.49910400 3.85049300

H -2.65729500 1.30873700 4.76796200

C -2.42429100 0.80826200 2.68191000

H -3.22867000 0.07145800 2.71594300

C 0.88829800 -1.91418500 -1.14016000

C 1.14213100 -2.23332900 -2.60310300

C -0.08221900 -3.05500900 -0.96901500

C -0.29857000 -2.16066900 -2.16436000

H 1.54730900 -3.23323500 -2.82599000

H 1.52699700 -1.43975200 -3.24659400

H 0.29604700 -4.07141300 -1.17136200

H -0.78770000 -3.00850700 -0.14413800

H -1.21541800 -1.86595900 -2.68308400

C -3.71331000 0.72189700 -0.67622800

C -4.72273700 1.18123700 0.18365300

C -3.95639100 0.69192500 -2.05847900

C -5.95606500 1.59182800 -0.32957000

H -4.55257100 1.23478100 1.25943500

C -5.19362600 1.09311800 -2.56915900

H -3.15701900 0.37467900 -2.73159400

C -6.19681000 1.54356100 -1.70621000

H -6.73105800 1.95437300 0.35088500

H -5.36712200 1.06749300 -3.64798500

H -7.16189500 1.86597100 -2.10591600

Cl -0.99440300 2.36945500 -1.89908200

**TS-S7**

C 1.08258200 3.80160300 0.55021500

C 2.45061600 3.51581900 0.39469100

C 3.04453900 2.43484500 1.04213100

C 2.25531000 1.63222400 1.87146200

C 0.91335500 1.94356000 2.11090800

C 0.33419400 3.02428600 1.44948600

H 3.05328800 4.12828100 -0.28075800

H 4.09496100 2.18884700 0.88231800

H 0.33937200 1.33590800 2.80926800

H -0.72374700 3.24369400 1.60627200

C 0.41911800 4.86225600 -0.28292700

H -0.44558400 5.30533100 0.23214000

H 0.05326800 4.38568400 -1.20964700

H 1.11913900 5.66394300 -0.56315100

S 2.89298000 0.06567000 2.43194100

O 2.00719100 -0.43740800 3.48666400

O 4.34639000 0.14840600 2.60891900

N 2.66081300 -0.95120600 1.09738300

C 1.28352800 -1.34441000 0.80072300

H 0.90411900 -2.05629700 1.55147100

H 0.64426100 -0.45360700 0.86009900

C 1.10065800 -1.91717800 -0.59307600

H 1.67011500 -1.39574800 -1.37097500

C -0.27922300 -2.22839600 -1.02084500

H -0.85569500 -2.76567600 -0.25397900

H -0.30766200 -2.79087200 -1.97204600

C 3.77791100 -1.33847300 0.25833500

H 4.66778000 -1.37548000 0.91451600

C 4.12382900 -0.42396900 -0.91617400

C 5.38649100 -0.55305000 -1.51643500

C 3.22773900 0.52530800 -1.41940300

C 5.73661400 0.23942000 -2.61165800

H 6.10483500 -1.27841200 -1.12108800

C 3.57181000 1.31526400 -2.52075300

H 2.24862700 0.67839000 -0.96565400

C 4.82566200 1.17414700 -3.11962800

H 6.72414800 0.13040600 -3.06734600

H 2.83108300 2.02500500 -2.89582400

H 5.09710500 1.79178600 -3.97950500

C 3.57294300 -2.78070500 -0.12905700

C 3.46903100 -3.39494500 -1.45942600

C 3.00366200 -3.84587400 0.70203000

C 2.18996800 -3.48981800 -0.58456400

H 3.99579500 -4.34545000 -1.62710800

H 3.44401700 -2.72543100 -2.32558000

H 3.49203100 -4.83130100 0.67752900

H 2.58230900 -3.56634600 1.67237600

H 1.34511600 -4.11327700 -0.88397100

C -3.95838200 -0.76908600 -1.04082200

C -3.86622600 -1.55323500 -2.20097300

H -2.87648300 -1.78640500 -2.60225200

C -5.02355500 -2.01392000 -2.83695000

H -4.93990600 -2.62102600 -3.74214900

C -6.28258200 -1.69049900 -2.32288800

H -7.18756800 -2.04505100 -2.82335500

C -6.38193300 -0.90420600 -1.16937700

H -7.36424200 -0.64342700 -0.76660800

C -5.22718200 -0.44578500 -0.53088700

H -5.31588000 0.17041600 0.36676000

C -2.39371600 -1.30701100 1.31623800

C -1.54394100 -0.97524500 2.38449700

H -1.00565100 -0.02904400 2.36568000

C -1.36226400 -1.84209100 3.46232300

H -0.67067000 -1.56444900 4.26140600

C -2.04390900 -3.06435400 3.49278200

H -1.90470800 -3.75065100 4.33210900

C -2.90359400 -3.40236800 2.44320700

H -3.44306400 -4.35313300 2.46241700

C -3.07744400 -2.53285100 1.36018700

H -3.74367000 -2.81661600 0.54299700

C -2.87800700 1.44098000 0.47318400

C -2.75118600 2.54637700 -0.38674600

C -3.37583200 1.64510400 1.77182800

C -3.11306000 3.82373500 0.04584000

H -2.33152000 2.41424600 -1.38584700

C -3.71989100 2.92981100 2.20730500

H -3.49756900 0.80333400 2.45529700

C -3.59064800 4.02284100 1.34586100

H -3.00429500 4.67124600 -0.63540400

H -4.09510800 3.07257800 3.22413700

H -3.85995700 5.02595700 1.68675400

Cl -0.01233200 1.84405200 -2.48552900

Rh -0.62959700 -0.22473200 -1.45755400

P -2.40138900 -0.22694700 -0.19124300

**TS-S8**

C -0.14320300 -1.10408600 3.26233700

C -0.22847400 -2.61181000 1.70107600

C 0.86392200 -1.80118000 2.36624400

H -0.71132900 -1.76070200 3.94659700

H 0.15114200 -0.15094300 3.70758700

H -0.80726700 -3.29451300 2.35016600

H -0.07747500 -3.02382000 0.69912700

Rh 1.29652500 -0.18498200 0.82102500

H 1.93493100 -1.99660900 2.50748700

Cl 1.63278600 1.45162300 2.57952600

P 2.11070700 1.39027600 -0.67671400

P 1.81307800 -1.68251400 -0.79761700

C 2.15636400 0.59278400 -2.35846100

C 3.88290100 1.84845600 -0.42891000

C 1.29340100 3.01528900 -0.91793600

C 2.74509600 -0.80687600 -2.17882300

C 3.03195900 -2.87629700 -0.08437600

C 0.66069800 -2.72779700 -1.80910700

H 1.11437900 0.54208800 -2.70788100

H 2.74258800 1.19290800 -3.07139000

C 4.61484900 1.27467400 0.62183400

C 4.52365400 2.73902300 -1.30732400

C 1.19592500 3.88687700 0.18050800

C 0.76284500 3.41003800 -2.15600700

H 3.80678900 -0.74200300 -1.89759200

H 2.67958400 -1.40843900 -3.09728100

C 4.34830700 -2.44423400 0.16371800

C 2.64086000 -4.13687700 0.40175400

C 1.03577600 -3.95423900 -2.38617700

C -0.61787300 -2.21508600 -2.07977200

H 4.10749500 0.61519300 1.32904400

C 5.97398100 1.57019000 0.77592000

C 5.87993200 3.03222100 -1.15153600

H 3.95525600 3.21699800 -2.11029500

H 1.58429500 3.57504900 1.15182600

C 0.57728400 5.12943500 0.03612900

C 0.14944700 4.65706100 -2.29380500

H 0.79939800 2.74404700 -3.01803300

H 4.67485000 -1.45277100 -0.15532100

C 5.25096800 -3.25718000 0.85205000

C 3.54433500 -4.94759500 1.09499400

H 1.62265400 -4.49557600 0.24597100

H 2.03571600 -4.35937700 -2.21816100

C 0.13404500 -4.66864200 -3.18026700

C -1.51820500 -2.93118400 -2.87464900

H -0.88910900 -1.23209800 -1.69417400

H 6.53397900 1.12150200 1.60053300

C 6.60889200 2.44377200 -0.11092400

H 6.36915600 3.72675000 -1.83958500

H 0.49904600 5.79457200 0.90018700

C 0.05240800 5.51846500 -1.19959900

H -0.27009400 4.94772600 -3.26033000

H 6.26832900 -2.90004200 1.03096800

C 4.85337300 -4.51362400 1.31997700

H 3.21845500 -5.92373200 1.46341300

H 0.43691700 -5.62449400 -3.61581700

C -1.14906100 -4.16474400 -3.41811700

H -2.51276500 -2.52104700 -3.06715000

H 7.67002100 2.67602500 0.01268600

H -0.44028300 6.48822900 -1.30731100

H 5.55849400 -5.14794300 1.86293100

H -1.85472400 -4.72933900 -4.03300400

C -6.13401400 -0.07073000 -1.78011200

C -5.00460000 -0.39291700 -2.54662500

C -3.74494800 0.12278900 -2.22806100

C -3.61458800 0.96818500 -1.12675200

C -4.72896600 1.33573900 -0.36633400

C -5.97553100 0.81212000 -0.69589000

H -5.10838600 -1.06154000 -3.40584100

H -2.86393300 -0.12841900 -2.81953900

H -4.61101800 2.00963600 0.48363700

H -6.84617800 1.08298500 -0.09244300

C -7.48591900 -0.66520300 -2.08290500

H -7.49662400 -1.18956600 -3.04973000

H -7.77221300 -1.39135300 -1.30262100

H -8.26891300 0.10972700 -2.10342100

S -1.98689700 1.56462800 -0.64482100

O -2.02265600 3.02092400 -0.52456500

O -1.04808300 0.91869300 -1.58416000

N -1.75666900 0.95756100 0.89411100

C -1.66014800 1.90930000 2.01687600

H -1.18594400 1.35495200 2.83533200

H -0.94038000 2.69268400 1.75155000

C -2.96722000 2.51282300 2.45991400

H -3.69848300 1.83123700 2.90819900

C -3.26243400 3.80907300 2.33057500

H -2.56254600 4.50024300 1.85123200

H -4.21463600 4.21859800 2.68126700

C -1.73862100 -0.50240700 1.03802600

H -1.64797400 -0.89603000 0.02213500

C -3.04056100 -1.06893300 1.60448400

C -3.43036900 -0.84668400 2.93459300

C -3.87377100 -1.83958400 0.78143600

C -4.64089100 -1.35129800 3.41607900

H -2.78349500 -0.27561900 3.60162500

C -5.08514500 -2.34638500 1.25938600

H -3.57966800 -2.02907400 -0.25338000

C -5.47612100 -2.09849100 2.57802900

H -4.93156100 -1.16360800 4.45300800

H -5.72570300 -2.93355600 0.59602800

H -6.42365800 -2.49152400 2.95547600

C -0.56788000 -1.13441900 1.80276000

**TS-S9**

C -7.84742000 -0.16525000 0.19782400

C -7.19677200 1.08340600 0.16223400

C -5.95693000 1.23280100 -0.45073100

C -5.34944800 0.11780400 -1.03859400

C -5.97498100 -1.12760400 -1.03503400

C -7.22107700 -1.26089500 -0.41319000

H -7.67070400 1.94921800 0.63291600

H -5.45166400 2.20041400 -0.46240200

H -5.48053400 -1.97741300 -1.50821200

H -7.71236400 -2.23759600 -0.40243500

C -9.18853700 -0.30087000 0.87378000

H -9.14928700 0.07250000 1.91023200

H -9.95575100 0.29202400 0.34778200

H -9.52992200 -1.34595500 0.90012500

S -3.70552900 0.29765800 -1.73833200

O -3.68918100 1.47988300 -2.60108400

O -3.32971300 -1.02606800 -2.26336700

N -2.78273600 0.65835900 -0.39046300

C -2.00906900 1.88804700 -0.31715900

H -2.51963100 2.63621300 -0.94422700

H -2.05322600 2.25630600 0.72034600

C -0.58259100 1.74113000 -0.78216400

H -0.09672100 2.72025100 -0.78112800

C -0.19746000 0.80595500 -1.80180400

H -0.92929200 0.08304500 -2.17746500

H 0.48519800 1.14537700 -2.59035500

C -2.55794500 -0.41668100 0.57643700

H -3.27325800 -1.19570700 0.27566400

C -3.00476000 0.01430900 1.96842000

C -4.33313200 -0.25018500 2.34617900

C -2.17361600 0.70948700 2.85563200

C -4.82159800 0.17206200 3.58467500

H -4.99779700 -0.78282900 1.66104500

C -2.66395600 1.12897700 4.09730500

H -1.12946100 0.89762500 2.59778200

C -3.98594800 0.86596800 4.46645700

H -5.85732900 -0.04390900 3.86039300

H -1.99487500 1.66184400 4.77806900

H -4.36407400 1.19530700 5.43808100

C -1.12763300 -1.01813200 0.39370500

C -1.20755500 -2.40329900 -0.21873500

C -0.43021900 -2.11885700 2.07425800

C -1.41152500 -2.59099400 1.23692800

H -0.28969500 -2.78864800 -0.65487700

H -2.08513300 -2.59885300 -0.84341100

H 0.61211700 -2.08827100 1.77950300

H -0.65344300 -1.85122300 3.10911200

H -2.42479800 -2.70315700 1.63043600

Rh 0.70098700 0.22940200 0.02436100

Cl 1.17201100 0.57006700 2.52098400

P 2.32724800 -1.54678000 -0.54327100

P 2.53812500 1.55436900 -0.55385000

C 3.70932000 -0.71513100 -1.54769700

C 1.72399100 -2.79024100 -1.77299700

C 3.37074000 -2.56071200 0.60703700

C 3.32783400 0.69304600 -2.00599000

C 2.22324800 3.27370600 -1.11076400

C 4.04706400 1.77514100 0.49383100

H 4.58361500 -0.66959400 -0.88127000

H 3.97304700 -1.35995600 -2.39873600

C 1.16471500 -2.29322900 -2.96600800

C 1.68153900 -4.17486200 -1.53693400

C 3.22009500 -2.37935100 1.99143400

C 4.36985300 -3.43706300 0.14175100

H 2.59276100 0.65863800 -2.82290600

H 4.20863700 1.25428900 -2.35550700

C 2.15511000 3.64703800 -2.46029600

C 1.96038500 4.23597700 -0.11776600

C 5.02699300 2.70822900 0.10346800

C 4.30392400 0.93642100 1.58603500

H 1.14126500 -1.21711200 -3.14582400

C 0.59487900 -3.15680200 -3.90198200

C 1.10409100 -5.03842900 -2.47429700

H 2.08526500 -4.58625800 -0.61021400

H 2.48747100 -1.66191800 2.37176800

C 4.03775100 -3.07422800 2.89079400

C 5.18562900 -4.12729200 1.03998100

H 4.50842800 -3.58509500 -0.93236300

H 2.34747900 2.91805700 -3.24957100

C 1.83523900 4.96351400 -2.81327400

C 1.64566000 5.54731100 -0.47253900

H 1.99474700 3.94606400 0.93618000

H 4.84525600 3.37536300 -0.74270800

C 6.23771500 2.79404500 0.79316100

C 5.52118800 1.01957600 2.27007100

H 3.53379600 0.24822900 1.92311800

H 0.15897000 -2.74956400 -4.81761500

C 0.56149200 -4.53478200 -3.65909200

H 1.07623800 -6.11240200 -2.27164800

H 3.90919800 -2.91868100 3.96512200

C 5.01834800 -3.94950700 2.41901800

H 5.95672500 -4.80507000 0.66400800

H 1.78342600 5.24282900 -3.86863700

C 1.58150100 5.91448200 -1.82280200

H 1.44404900 6.28631000 0.30701200

H 6.98770500 3.52585800 0.48158000

C 6.48989100 1.94536200 1.87756700

H 5.70313500 0.35494000 3.11838900

H 0.10620500 -5.21058000 -4.38735000

H 5.65787700 -4.48992100 3.12193500

H 1.33059600 6.94137400 -2.10040600

H 7.43917500 2.01163400 2.41586300

**TS-S10**

C -7.95773900 0.23979900 0.03126300

C -7.09327800 1.34291200 -0.10260000

C -5.82728300 1.20377500 -0.66368000

C -5.40724900 -0.06047300 -1.09321700

C -6.24801600 -1.16850700 -0.99094100

C -7.51854800 -1.01031000 -0.42829000

H -7.42070900 2.32668300 0.24495900

H -5.16447600 2.06531300 -0.76151900

H -5.89797900 -2.14003100 -1.34367400

H -8.17798200 -1.87821300 -0.34273000

C -9.32135400 0.41728800 0.65015400

H -9.85945300 -0.53796600 0.73580900

H -9.24326300 0.85678500 1.65829200

H -9.94153100 1.10261100 0.04835000

S -3.72752400 -0.26353600 -1.70067600

O -3.44056100 0.82384200 -2.63658200

O -3.59905400 -1.67158800 -2.10164600

N -2.76553800 -0.01643300 -0.35164800

C -2.07205500 1.26445100 -0.19121500

H -2.66879700 2.03709500 -0.70459100

H -2.08806400 1.53107800 0.87290100

C -0.66281000 1.34124400 -0.73456800

H -0.35256600 2.39138400 -0.74870800

C -0.15243600 0.50410500 -1.77160500

H -0.74174400 -0.35824100 -2.09785300

H 0.40737700 0.96134100 -2.59656300

C -2.91313600 -0.97441700 0.75273400

H -3.71629400 -1.66167300 0.44545900

C -3.38735000 -0.32876900 2.04892200

C -4.75683000 -0.37040300 2.35767600

C -2.51918200 0.35226100 2.91808600

C -5.25102900 0.25063300 3.50754700

H -5.44901500 -0.88509200 1.68730600

C -3.01510800 0.97080500 4.06991300

H -1.44740900 0.41670000 2.70582300

C -4.37976900 0.92360800 4.36946800

H -6.32085100 0.20657100 3.72832100

H -2.31975200 1.49352200 4.73164400

H -4.76342500 1.40887700 5.27093800

Rh 0.97399200 0.12633600 -0.00740000

Cl 1.01129500 0.49292900 2.52318700

C -1.71512400 -1.90407100 0.85364800

C -0.97160800 -2.33547000 1.98683100

C -1.08635800 -2.65144800 -0.29099000

C -0.24704000 -1.69129800 0.49591800

H -0.65982700 -3.37989400 2.08210100

H -0.87924000 -1.69911500 2.86808900

H -0.94601100 -3.73544100 -0.17880900

H -1.38203000 -2.33082800 -1.29126800

H 0.70788000 -1.88184700 1.00285000

P 2.73316200 -1.35673100 -0.57539100

P 2.51340400 1.67566900 -0.65240600

C 3.97453700 -0.43996200 -1.66079300

C 2.10473500 -2.72409600 -1.63748200

C 3.82235500 -2.23175500 0.62949400

C 3.41255100 0.91560500 -2.10322100

C 1.94732000 3.32075800 -1.22992400

C 3.94295000 2.09696200 0.43487800

H 4.87310000 -0.29525100 -1.04155600

H 4.25575700 -1.06488300 -2.52151700

C 1.49270800 -2.38791700 -2.85902300

C 2.06558300 -4.06006100 -1.20711600

C 3.51534900 -2.12536200 1.99560400

C 4.94977000 -2.97338900 0.23226800

H 2.67718600 0.78908400 -2.91130100

H 4.20792300 1.58731200 -2.46254100

C 1.77343800 3.64234900 -2.58393900

C 1.57772000 4.25899900 -0.24847400

C 4.88656700 3.04689400 -0.00012500

C 4.15918200 1.41259000 1.63828300

H 1.47355200 -1.34736200 -3.18854500

C 0.87174600 -3.36533800 -3.63718100

C 1.43337200 -5.03698900 -1.98396100

H 2.52419400 -4.34291400 -0.25743000

H 2.65905400 -1.52388700 2.31848900

C 4.32333100 -2.75807700 2.94821400

C 5.75679500 -3.59700800 1.18552500

H 5.19336300 -3.07161600 -0.82929700

H 2.04166200 2.92831200 -3.36471600

C 1.24899100 4.88660200 -2.95212100

C 1.05785300 5.49911100 -0.61880000

H 1.69150500 4.00710500 0.80956900

H 4.72105600 3.59982800 -0.92888400

C 6.03450700 3.29628700 0.75303500

C 5.31373700 1.66433900 2.38829800

H 3.40509900 0.71665500 2.00526500

H 0.39748700 -3.08539500 -4.58114700

C 0.83578200 -4.69458900 -3.19952800

H 1.40792400 -6.07220600 -1.63318300

H 4.07743600 -2.66649700 4.00934000

C 5.44274100 -3.49109100 2.54659600

H 6.63226100 -4.16978700 0.86806100

H 1.11751800 5.12610400 -4.01031700

C 0.89210400 5.81607300 -1.97279400

H 0.77541400 6.22051200 0.15208400

H 6.76081300 4.03687300 0.40795000

C 6.25272000 2.59946900 1.94834900

H 5.47070600 1.12419300 3.32529300

H 0.33798600 -5.45789300 -3.80274600

H 6.07519300 -3.98024700 3.29223300

H 0.48096700 6.78645900 -2.26235700

H 7.15309500 2.79375900 2.53734000

**TS-S11**

C 7.93400000 -0.78893300 2.22981200

C 6.85761000 -0.54394500 3.09614200

C 5.56675700 -0.98233900 2.79054000

C 5.34853500 -1.66779600 1.59390500

C 6.40620100 -1.94866200 0.72131500

C 7.68716300 -1.50575300 1.04443100

H 7.03167800 -0.00805500 4.03300100

H 4.73679200 -0.81684100 3.47945700

H 6.22040900 -2.52251400 -0.18811500

H 8.51713800 -1.72737100 0.36757800

C 9.32496400 -0.30109200 2.54755000

H 9.39724500 0.08881600 3.57327300

H 10.06624900 -1.10848500 2.43440200

H 9.62243000 0.50818200 1.85896000

S 3.68623900 -2.17136300 1.15238700

O 2.92068200 -2.26743200 2.39702900

O 3.80754400 -3.31734700 0.23627400

N 3.07424500 -0.89826400 0.25274300

C 2.59449900 0.29330000 0.94589600

H 3.01891700 0.28850500 1.96341400

H 3.01291700 1.17694200 0.43674800

C 1.09132400 0.41630800 1.04187500

H 0.81530300 1.36014300 1.52545900

C 0.21303900 -0.68309400 1.16771600

H 0.62427900 -1.69616300 1.16147700

H -0.70196100 -0.57933600 1.75885000

C 2.90718500 -1.02035600 -1.20348900

H 3.53629500 -1.87515700 -1.48358200

C 3.49198000 0.20182700 -1.91043200

C 4.85107900 0.48504100 -1.68857000

C 2.75684700 1.05328100 -2.74399300

C 5.46048100 1.59200700 -2.27917300

H 5.43260700 -0.16120900 -1.02697400

C 3.36913200 2.16469800 -3.33878800

H 1.69797400 0.87730300 -2.92693700

C 4.71781900 2.44082900 -3.10932300

H 6.51776200 1.79495300 -2.08897700

H 2.77194500 2.81794000 -3.98049600

H 5.19090900 3.31127500 -3.57144900

C 1.41022500 -1.32420900 -1.44549500

C 0.97458000 -2.77059100 -1.23087300

C 0.88386800 -1.74281900 -3.42720700

C 1.45895300 -2.69640800 -2.62031200

H -0.10165900 -2.88726600 -1.06407800

H 1.59651000 -3.38860900 -0.57600800

H -0.15120800 -1.41567900 -3.33250900

H 1.43145500 -1.35530100 -4.29134900

H 2.46236400 -3.06659600 -2.84541800

Rh 0.00395700 0.18353000 -0.76577200

Cl -0.86720500 0.83201700 -2.96118200

P -1.57286900 1.74249300 -0.07992300

C -3.29237400 1.31818300 -0.67406400

C -1.81979500 1.99845100 1.73495300

C -1.28247800 3.46292100 -0.66365400

C -3.45129900 -0.19561200 -0.86062500

H -4.01698500 1.73458100 0.04301800

H -3.42454000 1.83526100 -1.63464500

C -2.69928300 1.18095200 2.46663900

C -1.05968600 2.95804000 2.42770600

C -2.24820500 4.46614000 -0.47822600

C -0.06632700 3.78511200 -1.28433400

H -2.96822800 -0.75690600 -0.04439800

H -2.93288600 -0.47695300 -1.78800800

P -5.22943800 -0.75525800 -0.99958600

H -3.31055900 0.42721000 1.96884900

C -2.81933800 1.32499400 3.85165100

C -1.17835000 3.09777600 3.81297300

H -0.37923600 3.61295200 1.87794300

H -3.19463600 4.23116700 0.01622900

C -2.00287200 5.77050600 -0.91307500

C 0.18114100 5.09362100 -1.71026100

H 0.67874800 3.00281100 -1.44322300

C -4.99289000 -2.58775000 -1.13280400

C -5.68655100 -0.58927800 0.79617300

H -3.51877100 0.68436600 4.39322400

C -2.05839700 2.28109300 4.52982600

H -0.58316300 3.85298800 4.33305900

H -2.76177000 6.54398900 -0.76885000

C -0.78603400 6.08637200 -1.52870400

H 1.13245700 5.33322000 -2.19239600

C -6.13211000 -3.40394200 -0.99165400

C -3.76317100 -3.20577400 -1.42135300

C -6.41689600 0.54626600 1.18493500

C -5.26509700 -1.49165000 1.78979800

H -2.15313600 2.39257700 5.61302200

H -0.59391500 7.10803600 -1.86706800

H -7.10123700 -2.94501600 -0.77343900

C -6.04327900 -4.79105300 -1.11385700

C -3.67695900 -4.59672500 -1.55530000

H -2.85564000 -2.61216200 -1.54399200

H -6.76339900 1.25013000 0.42248600

C -6.70252900 0.78806500 2.53325100

C -5.56106900 -1.25789400 3.13504900

H -4.69836000 -2.38281500 1.50873000

H -6.93960100 -5.40499100 -0.99144600

C -4.81240100 -5.39427200 -1.39853600

H -2.71064900 -5.05686400 -1.77946600

H -7.26377900 1.68155000 2.81883400

C -6.27684700 -0.11482800 3.51091000

H -5.22676200 -1.96924000 3.89512900

H -4.74152800 -6.48027700 -1.49955600

H -6.50396300 0.06906700 4.56422800

**TS-S12**

C 8.31085700 0.01306900 0.63598100

C 7.43797500 0.07036300 1.73350000

C 6.23033600 -0.63182900 1.73667700

C 5.88550600 -1.39561700 0.61917400

C 6.74857700 -1.49393400 -0.47817900

C 7.95020600 -0.78855600 -0.46255700

H 7.71107100 0.66675800 2.60814600

H 5.56826600 -0.61300100 2.60398500

H 6.48554800 -2.13959800 -1.31791600

H 8.63012800 -0.86934800 -1.31531900

C 9.60112500 0.79311900 0.62107200

H 9.88717100 1.12832100 1.62883800

H 10.42822700 0.19335000 0.20973500

H 9.50564800 1.69119000 -0.01360500

S 4.31053600 -2.25213200 0.57509400

O 3.86333300 -2.40830700 1.96003500

O 4.46167700 -3.40489500 -0.32192200

N 3.23663500 -1.20062600 -0.18019300

C 2.83061000 -0.00930000 0.56519800

H 3.58227500 0.16435700 1.35416600

H 2.91216800 0.86074300 -0.10475700

C 1.46978500 -0.01198200 1.22642500

H 1.34885500 0.92358700 1.78674000

C 0.75722400 -1.13863400 1.68784100

H 1.16575800 -2.13795200 1.52802500

H 0.13284500 -1.04429400 2.58155100

C 2.96016300 -1.37427600 -1.61536100

H 3.74810200 -2.04442300 -1.99401200

C 3.09219700 -0.07174700 -2.39164800

C 4.38065600 0.32537900 -2.78706500

C 2.01417100 0.77998900 -2.66545900

C 4.58764800 1.54022400 -3.44287900

H 5.23463800 -0.32033700 -2.56815700

C 2.21787600 1.99407000 -3.33072600

H 1.00412800 0.52214800 -2.34621300

C 3.50264300 2.37980300 -3.72017200

H 5.59826400 1.83053900 -3.74133800

H 1.35764800 2.63709700 -3.53160400

H 3.66070400 3.32936700 -4.23818200

Rh -0.19866400 -0.33935900 -0.03573400

Cl -1.52294100 0.22591900 -2.01418200

C 1.72374500 -2.22825100 -1.84127300

C 0.76693600 -2.22142200 -2.88297300

C 1.27482300 -3.31993200 -0.93112100

C 0.32435800 -2.15934200 -1.14198000

H 0.37081100 -3.16310500 -3.27048100

H 0.57381200 -1.32090200 -3.46658300

H 1.04199400 -4.28777700 -1.39473600

H 1.77079800 -3.39492300 0.03631000

H -0.74007400 -2.26875500 -1.37648300

P -1.29703800 1.39694400 0.97470300

C -3.15740900 1.27305400 0.85631700

C -1.00879200 1.64992400 2.78377500

C -0.93286900 3.06409800 0.28124200

C -3.68330400 -0.16440400 0.84472300

H -3.59655900 1.85614500 1.68152400

H -3.41650900 1.77480500 -0.08722400

C -1.57035800 0.74721700 3.70520000

C -0.17301300 2.67093400 3.26749700

C -1.75835600 4.17320500 0.53141900

C 0.21487800 3.23121000 -0.50726800

H -3.34265900 -0.73042000 1.72582300

H -3.29221800 -0.70162200 -0.03149000

P -5.55441400 -0.27448400 0.91605200

H -2.20825900 -0.06700500 3.35622100

C -1.31820300 0.87369200 5.07301400

C 0.08516900 2.79130300 4.63671300

H 0.27518600 3.38369400 2.57204800

H -2.65326200 4.06696700 1.14927300

C -1.44029400 5.42438900 -0.00268000

C 0.53723200 4.48480200 -1.03475200

H 0.84885000 2.36832200 -0.71559900

C -6.06524200 0.34015300 -0.75612100

C -5.69508900 -2.11588400 0.69344700

H -1.76887900 0.16599100 5.77344600

C -0.48786700 1.89608600 5.54357700

H 0.73578800 3.59351200 4.99475000

H -2.09222800 6.27961700 0.19331900

C -0.29099700 5.58296000 -0.78557100

H 1.43510200 4.59613700 -1.64807500

C -7.42402900 0.66937700 -0.91702700

C -5.19970000 0.50539900 -1.85215300

C -6.12731400 -2.88168200 1.78822600

C -5.35171200 -2.77624900 -0.49959600

H -0.28739600 1.99246600 6.61361500

H -0.04423200 6.56304000 -1.20221200

H -8.10812400 0.56179300 -0.06964500

C -7.91086900 1.13113000 -2.14219500

C -5.68578800 0.98249300 -3.07433100

H -4.13642600 0.26949800 -1.77693000

H -6.40706000 -2.37925500 2.71896400

C -6.20612300 -4.27612100 1.69999100

C -5.43429300 -4.16758000 -0.59022900

H -5.02243400 -2.19825000 -1.36673300

H -8.97141800 1.37481900 -2.24912700

C -7.04018700 1.29207700 -3.22558500

H -4.99253700 1.10901400 -3.91019300

H -6.54373300 -4.85809100 2.56185800

C -5.86024400 -4.92141200 0.50994800

H -5.16662600 -4.66720300 -1.52534200

H -7.41693200 1.66281400 -4.18266100

H -5.92550400 -6.01026300 0.43668900
